# Genomic analysis of hybrid rice varieties reveals numerous superior alleles that contribute to heterosis

**DOI:** 10.1038/ncomms7258

**Published:** 2015-02-05

**Authors:** Xuehui Huang, Shihua Yang, Junyi Gong, Yan Zhao, Qi Feng, Hao Gong, Wenjun Li, Qilin Zhan, Benyi Cheng, Junhui Xia, Neng Chen, Zhongna Hao, Kunyan Liu, Chuanrang Zhu, Tao Huang, Qiang Zhao, Lei Zhang, Danlin Fan, Congcong Zhou, Yiqi Lu, Qijun Weng, Zi-Xuan Wang, Jiayang Li, Bin Han

**Affiliations:** 1 National Center for Gene Research, Collaborative Innovation Center for Genetics and Development, Institute of Plant Physiology and Ecology, Shanghai Institutes for Biological Sciences, Chinese Academy of Sciences, Shanghai 200233, China; 2 State Key Laboratory of Rice Biology, China National Rice Research Institute, Chinese Academy of Agricultural Sciences, Hangzhou 310006, China; 3 Institute of Plant Protection and Microbiology, Zhejiang Academy of Agricultural Sciences, Hangzhou 310021, China; 4 National Center for Plant Gene Research, State Key Laboratory of Plant Genomics, Institute of Genetics and Developmental Biology, Chinese Academy of Sciences, Beijing 100101, China

## Abstract

Exploitation of heterosis is one of the most important applications of genetics in agriculture. However, the genetic mechanisms of heterosis are only partly understood, and a global view of heterosis from a representative number of hybrid combinations is lacking. Here we develop an integrated genomic approach to construct a genome map for 1,495 elite hybrid rice varieties and their inbred parental lines. We investigate 38 agronomic traits and identify 130 associated loci. In-depth analyses of the effects of heterozygous genotypes reveal that there are only a few loci with strong overdominance effects in hybrids, but a strong correlation is observed between the yield and the number of superior alleles. While most parental inbred lines have only a small number of superior alleles, high-yielding hybrid varieties have several. We conclude that the accumulation of numerous rare superior alleles with positive dominance is an important contributor to the heterotic phenomena.

## Abstract (web summary)

The principle of heterosis, whereby a hybrid variety outperforms both parents, is commonly exploited in agriculture to maximize crop yield. Here Huang *et al.* show that the accumulation of multiple superior alleles in hybrid varieties contributes to hybrid vigour and increased yield in rice.

## Graphical abstract

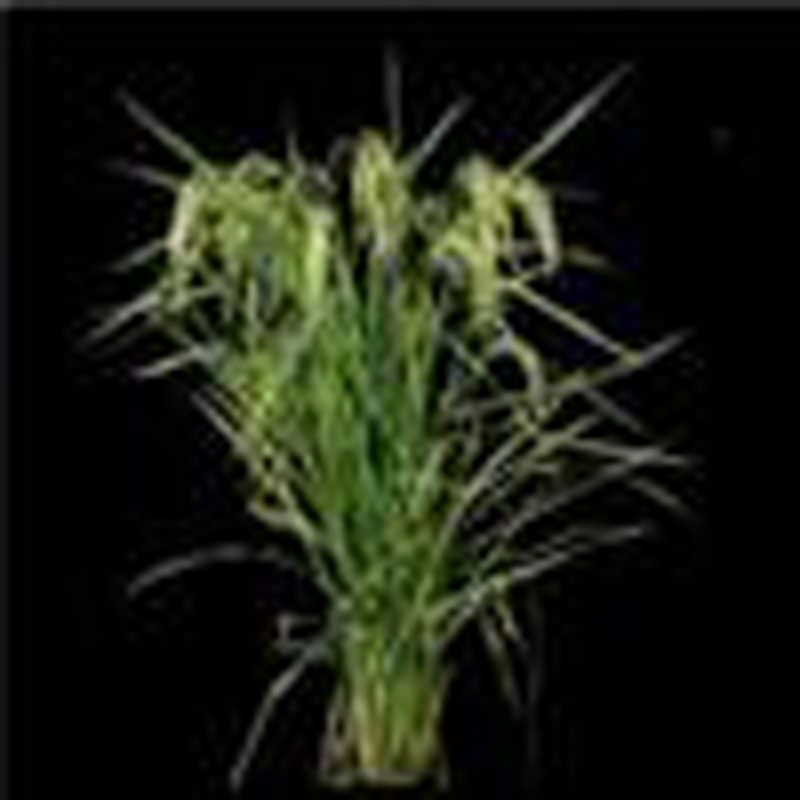

The phenomenon that the heterozygous first filial (F_1_) generation often has better performance than its homozygous parents is known as heterosis or hybrid vigour^1^,^2^,^3^. The development of heterotic crops, especially those for hybrid rice and maize, is one of the most important applications of genetics in agriculture, and now over half of the rice and maize production worldwide is from hybrid seeds that lead to tremendous increases in yield^4^. Although rice is a self-pollinated organism and nearly all traditional rice cultivars are inbred lines, a hybrid seed production mechanism has been developed using systems based on cytoplasmic (three-line hybrid system) and environmentally sensitive (two-line hybrid system) genetic male sterility since the 1970s^5^,^6^. The genetic mechanism of heterosis has been explained by three non-mutually exclusive hypotheses, including dominance (complementation)^7^,^8^, overdominance^9^,^10^ and epistasis^11^,^12^. Further molecular genetic and genomic approaches have been used to investigate the heterotic performances in plants^13^,^14^,^15^,^16^,^17^,^18^,^19^. Single-locus overdominance of heterozygous alleles has been shown to result in heterosis straightway in *Arabidopsis*^13^, tomato^14^ and maize^15^. In rice, quantitative trait locus analysis in an *indica*–*japonica* hybrid suggested that dominance complementation was the major cause of heterosis^16^. Recently, the genetic dissection of yield traits using an ‘immortalized F_2_’ population from an *indica*–*indica* rice hybrid cross enabled the assessment of genetic composition of yield heterosis^17^, which showed that the relative contributions of the genetic components varied with different yield traits^18^. Moreover, a genomic and metabolic approach has been applied to predict complex heterotic traits in hybrid maize^19^.

To elucidate the genetic basis of rice heterosis, we developed an integrated genomic framework that exploited population-scale genomic landscapes from a representative number of hybrid rice varieties and parental lines to map the heterotic loci at fine scales. We collected and sequenced 1,495 diverse varieties of hybrid rice (F_1_), which are grown on >15 million hectares per annum and contribute greatly to the agricultural production^4^. The hybrid rice varieties were extensively phenotyped for grain yield, grain quality and disease-resistance traits. This approach enabled us to analyse the genomic structures of the rice hybrids and to identify the heterotic loci and the genetic effects of both the homozygous and heterozygous genotypes. This research provides new insights into the principles of hybrid vigour and has implications for rice breeding.

## Results

### Genomic architecture and heterozygosity of rice hybrids

In an attempt to investigate as many rice hybrid combinations as possible, we sampled a total of 1,495 diverse varieties of hybrid rice (Supplementary Data 1), many with publicly available agricultural production statistics and pedigrees. Nearly all elite rice hybrids that are widely cultivated during recent years were included in the collection. The hybrids were sequenced with twofold genome coverage (an average of 2.2x), and a total of 1.2 Tb of genome sequence was generated (Supplementary Fig. 1). After sequence alignment of all the paired-end reads against the rice reference genome sequence, we called genotypes of the hybrid rice at 1,654,030 single-nucleotide polymorphism (SNP) sites (>4 SNPs per kb on average). Using the software Beagle^20^, a fine-scale genome map for all the hybrid rice varieties was therefore generated. To estimate the accuracy of the inferred genotypes, four hybrid rice varieties were sequenced independently at a high coverage (~40x genome coverage for each), and genotype calls from the deep-sequencing data were consistent with the imputed genotypes at a specificity of over 97.8% (Supplementary Table 1). We also sequenced 90 inbred lines (an average of 14.5x genome coverage) that were commonly used as the parents of hybrid rice (Supplementary Table 2). To evaluate the data sets from a number of hybrid combinations, we further analysed the genomes of 35 parents–child trios, in which the F_1_ hybrids and both their parents were sampled and sequenced in this study. In the 35 trios, the experimentally determined genotypes from both the parents are highly concordant (97.5%) with the imputed genotypes of the corresponding F_1_ hybrids (Supplementary Fig. 2). Hence, the independent data sources confirmed the accuracy of the genotypes of the rice hybrids sequenced.

**Figure 1:**
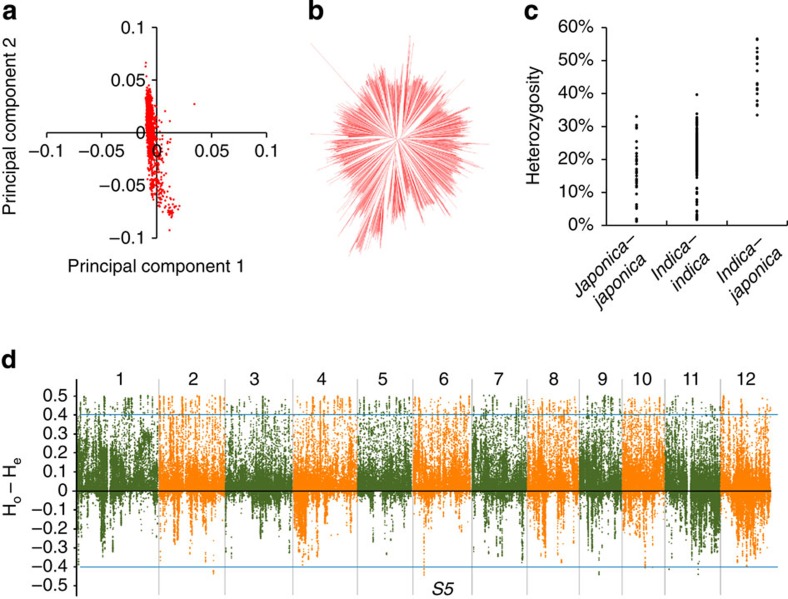
Genetic structure and heterozygosity of rice hybrid varieties. (**a**) Plots of the first two principal components of 1,439 rice hybrid varieties. (**b**) NJ tree of 1,439 *indica* hybrids constructed from simple matching distances of whole-genome SNPs. (**c**) Distribution of whole-genome heterozygosity of all the hybrids. (**d**) Heterozygosity plots of whole-genome SNPs in *indica* hybrids. The H_o_ and the H_e_ ( by the Hardy–Weinberg equation) were calculated for each SNP in the rice genome. The thresholds for highly heterozygous SNPs (H_o_−H_e_>0.4) and extremely low-heterozygous SNPs (H_o_−H_e_<−0.4) are indicated by horizontal lines. *S5* (Hybrid sterility-5) locus is indicated.

We used the SNP data to investigate the population structure of the rice hybrids. *Indica* and *japonica* are two major subspecies in cultivated rice (*Oryza sativa* L.). Of the 1,495 hybrid rice varieties, the majority (1,439 varieties) was from *indica*–*indica* crosses (Fig. 1a and Supplementary Fig. 3). The rest belonged to either *indica*–*japonica* (*n*=18) or *japonica*–*japonica* crosses (*n*=38). *Indica*–*japonica* and *japonica*–*japonica* hybrids have not been fully applied in agriculture up to now, probably due to the partial reproductive isolation in *indica*–*japonica* crosses and the limited diversity of *japonica* subspecies for *japonica*–*japonica* hybrids. According to the neighbour-joining (NJ) tree generated from whole-genome SNPs, there were no obvious population differentiations within the *indica* hybrids (Fig. 1b). We observed that the relationships depicted in the NJ tree agreed well with our pedigree-based expectations—the hybrid varieties derived from the same maternal lines (male sterile lines) tended to be clustered in the same clades in the NJ trees (Supplementary Fig. 4).

We further analysed the heterozygosity of the hybrid rice varieties. For each hybrid rice variety, the level of heterozygosity was measured as the frequency of the heterozygous genotype at the 1.65 million SNP sites. As expected, the group of *indica*–*japonica* crosses has the highest level of heterozygosity (45.1% on average, with the top one reached 56.6%), followed by *indica*–*indica* crosses (21.8% on average) and *japonica*–*japonica* crosses (15.8% on average; Fig. 1c). We detected the genomic regions with unusual heterozygosity based on the Hardy–Weinberg equilibrium (HWE). HWE is an ideal situation, while selection or nonrandom mating can disequilibrate HWE at least for some local genomic loci. In the population of 1,439 *indica* hybrids, we compared the observed heterozygosity (H_o_) with the expected heterozygosity (H_e_, calculated by the HWE) at each SNP site and observed that many SNPs tended to have higher heterozygosity (Supplementary Fig. 5). We used ‘H_o_−H_e_’ to estimate the excess of homozygotes above the Hardy–Weinberg expectations in the population, and detected 3,520 highly heterozygous SNPs (H_o_−H_e_>0.4, 0.2% of the total SNPs) that were distributed randomly throughout the whole genome and eight genomic loci with extremely low-heterozygous SNPs (H_o_−H_e_<−0.4, Fig. 1d). The eight loci with the loss of heterozygosity may be related to hybrid incompatibility that exists within the rice species (Supplementary Table 3). For example, the one with extremely low heterozygosity in chromosome 6 was found to be located near Hybrid sterility-5 (*S5*) locus that was characterized to control reproductive isolation and compatibility in rice^21^.

### Analysis of the parental lines

**Figure 2:**
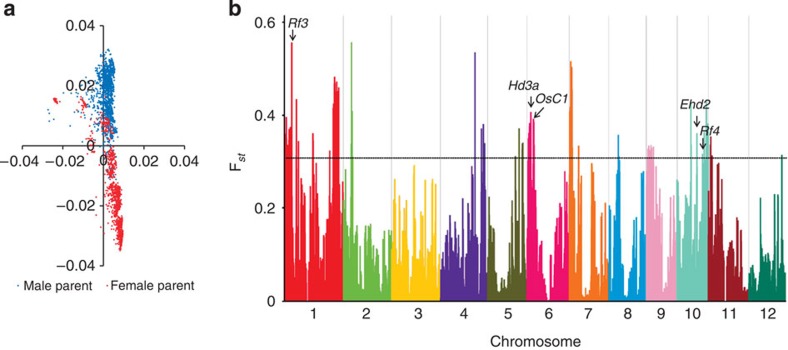
Analysis of the hybrids’ parental lines. (**a**) Principal-component analysis of the parental lines of *indica* hybrids. The male sterile lines (female parents) and the restorer lines (male parents) are coloured in red and blue, respectively. (**b**) Genomic screening of highly differentiated loci between the maternal and paternal lines. The level of population differentiation (*F*_st_) was computed in each 100-kb window of the rice genome. Whole-genome SNP data of the parental lines of *indica* hybrids were used in the calculation. The threshold (*F*_st_>0.3) is indicated by horizontal dash-dot lines. The candidate genes or quantitative trait locus that may involve in the differentiation between maternal and paternal lines are indicated.

We then tried to infer the original genotypes of the hybrids’ parental lines. Since the seeds of parental lines for the hybrid varieties are largely not available, the genomic information of most hybrid parents cannot be obtained directly. We therefore developed a computational pipeline (see Methods for details) that successfully delineated the maternal and paternal copies for 1,361 hybrids (91% of the collection), resulting in 288 non-redundant male sterile lines (female parent) and 621 non-redundant restorer lines (male parent) in the hybrid breeding. We investigated the population structure of the parental lines. On the basis of the NJ tree, a genetic distinction was observed between the maternal and paternal lines (Fig. 2a and Supplementary Fig. 6). The average amount of population differentiation between all the *indica* maternal lines and all the *indica* paternal lines was calculated to be modest (*F*_st_=0.08; Supplementary Fig. 7). We scanned the whole genome and found 31 highly differentiated loci between the maternal and paternal lines (Fig. 2b and Supplementary Table 4). These loci were probably involved in the heterotic phenomenon (for example, *Hd3a*) or fertility restoration (for example, *Rf3* and *Rf4* (refs 22, 23)).

In the search for the genomic impact of prior selections for agronomic traits during modern breeding, the genome data from diverse rice landraces reported previously^24^,^25^,^26^ was compared with the data of modern varieties sequenced here. Through genome-wide screening, we identified 20 loci of very strong selection under recent breeding (Supplementary Fig. 8 and Supplementary Tables 5 and 6). We found that there are no overlaps between the genetic-improvement sweeps in *indica* and those in *japonica*, suggesting that few introgression events between the two subspecies occurred for desired alleles during modern hybrid breeding. Of the 20 genetic-improvement sweeps, six sweeps were overlapped with domestication loci as well. The overlaps indicated that a subset of domestication alleles that were not completely fixed in landrace populations had continually been selected in recent breeding.

### Large-scale GWAS on agronomic traits

The hybrid varieties were planted together for phenotyping at two agro-ecologically diverse locations—Sanya (short-day condition, tropics region) and Hangzhou (long-day condition, subtropics region) in China (Supplementary Data 2 and 3). The agronomically important traits were evaluated comprehensively, including grain yield (components and relevant factors), grain quality (appearance and cooking characteristics) and disease resistance (blast and bacterial blight). The traits phenotyped here were highly related to current genetic improvements of hybrid rice. Moreover, four yield traits measured among regional tests were also used for our analysis. Taken together, a total of 38 sets of phenotypes were finally collected. The traits showed wide variation in the diverse rice hybrid panel. We found that the *indica* hybrids generally had better yield performance and stronger resistance to rice blast disease than *japonica* varieties (Supplementary Table 7). However, most *indica* hybrid varieties showed disadvantages in grain quality (for example, chalky grain rate) as compared with *japonica* (Supplementary Table 8). Of all the traits, panicle number and grain number showed the highest correlation with grain yield in the trials (Supplementary Fig. 9).

**Figure 3:**
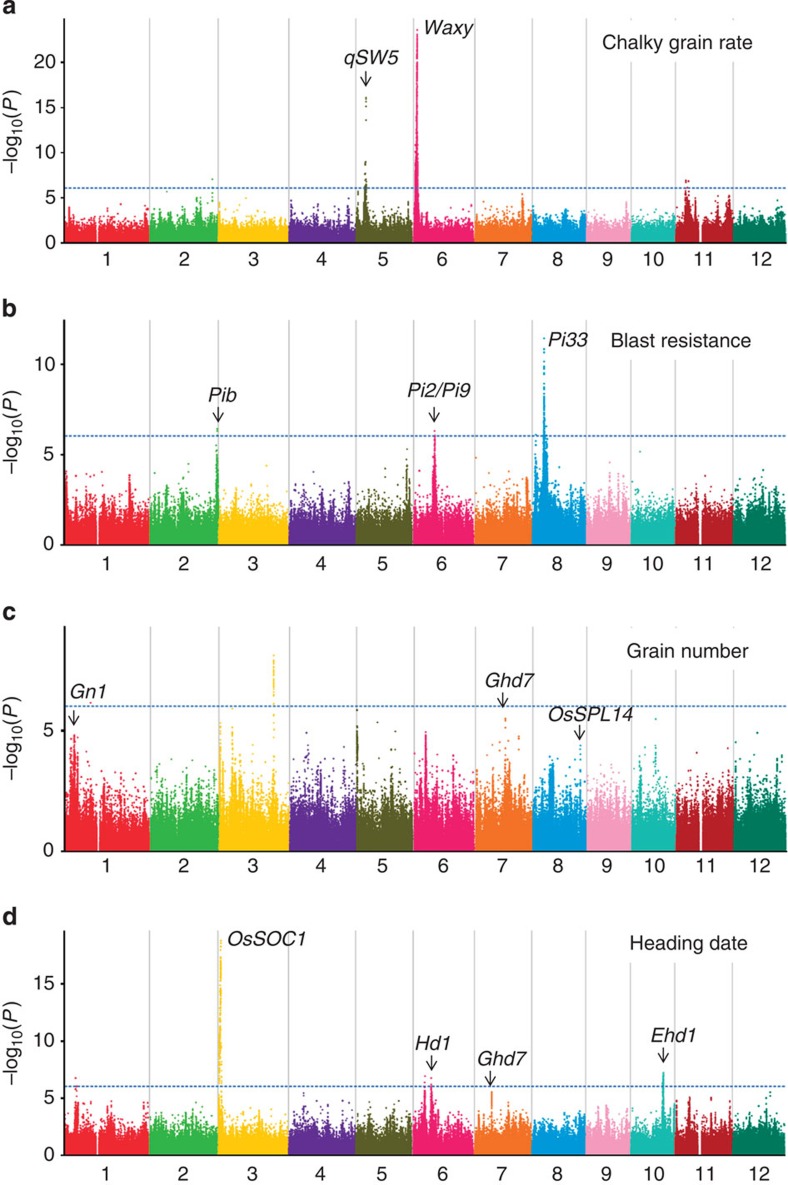
GWAS of heading date, gain number, blast resistance and chalky grain rate in Sanya. Negative log_10_ *P* values the from linear mixed model (*y* axis) are plotted against SNP positions (*x* axis) on each of the 12 rice chromosomes. This Manhattan plot is the result of the additive model for the traits chalky grain rate (**a**) blast resistance (**b**) gain number (**c**) and heading date (**d**). The genome-wide significant *P* value threshold (10^−6^, from the linear mixed model) is indicated by a horizontal dash-dot line. The loci with well-characterized genes are indicated near the association peaks.

Large-scale genome-wide association study (GWAS) analyses for the complex agronomic traits were carried out using Efficient Mixed-Model Association eXpedited (EMMAX) that can correct for cryptic relatedness and pedigree structure^27^ (Fig. 3 and Supplementary Figs 10–18). To avoid strong population structure, both the *indica*–*japonica* and *japonica*–*japonica* crosses (both with small sample number) were not used in further genetic analyses. The inheritance models (additive, dominant and recessive) were all tested to look for the strongest associations at each SNP site. Totally 130 association signals were identified using 10^−6^ as genome-wide significant threshold (false discovery rate (FDR)<0.01 according to permutation tests). The associations here included 53 loci with well-characterized genes and 77 novel associations firstly identified here (Supplementary Tables 9–11 and Supplementary Fig. 19). Moreover, we recoded SNP genotypes—both the homozygous states were coded as one type and the heterozygous genotype was coded as the other type and looked for associations for the four yield-related traits. Among the 13 association signals identified by this approach, 10 were also significant in the additive model (Supplementary Table 12 and Supplementary Figs 20 and 21).

For grain quality traits, 27 associated loci were identified. The GWAS loci *Waxy*, *ALK* and *qSW5* showed very strong effects on grain quality^28^,^29^,^30^ (Fig. 3a), while other loci had ‘fine tuning’ effects. Since the three major loci are not closely linked on rice chromosomes, the allelic combination of the loci could create a large number of types that may result in a varied palatability of cooked rice (3^3^=27 combinations, in principle). By screening all the *indica* hybrids in our collection, there were 26 combinations with various amylose contents, chalky grain rates and alkali digestion values. Interestingly, half of the *indica* hybrids belong to two common combination types, while the type with high grain quality only occupied a small proportion (~5.9%, Supplementary Fig. 22).

Totally 10 associated loci were identified for resistance to the blast fungus *Magnaporthe grisea* and leaf-blight bacteria *Xanthomonas oryzae*. The associations for rice blast resistance were mostly located around NBS-LRR gene clusters, including *Pib*, *Pi2/Pi9* and *Pi33* genes^31^,^32^,^33^ (Fig. 3b). We investigated the frequency of resistance alleles in the rice hybrids and found that most of them have a modest frequency. The only two exceptions were *Pi2/Pi9* cluster and an association for leaf-blight on chromosome 6, both with rare frequency of resistance alleles (3.7 and 2.6%, respectively) in hybrid rice. Interestingly, both the loci were located near or within selective sweeps, which implied that the susceptibility alleles or their nearby haplotypes might have some positive effects on the output yield.

We found less strong association signals for yield component traits panicle number and grain number per panicle^34^,^35^,^36^,^37^,^38^ (Fig. 3c). It is probably because the key components of grain yield were affected by numerous alleles with minor effects, and the situation is in high contrast to those of disease resistance, grain quality, grain weight and heading date. Totally 98 clear association signals were identified for grain number, panicle number, yield per plant above the suggestive *P* value of 10^−4^ (from the linear mixed model), and many genes that have been well-characterized to control the traits were just located within the associated loci (Supplementary Table 13 and Supplementary Fig. 23). Hence, the associations, despite only with modest significant *P* value (FDR<0.2 according to permutation tests), would greatly contribute to a deep mining of superior alleles.

Among GWAS peaks underlying the traits in Hangzhou and Sanya, we detected five significant associations (*P*<10^−6^, from the linear mixed model) with extremely high heterozygosity (H_o_>0.6), which resulted from allele differentiation between the maternal and paternal lines (Supplementary Table 14). The associations include two loci underlying plant height (*Hd3a* and *Hd1*)^39^,^40^, two loci underlying heading date (*Hd1* and *Ehd1*, Fig. 3d)^40^,^41^ and *OsC1* controlling the green or purple leaf sheath^42^ that help to distinguish the maternal and paternal lines in the field production.

### Trait–trait dynamics and genotype–environment interaction

We investigated the genetic effects of the superior alleles of yield-related traits on yield per plant, and found that only a small fraction of the associated loci for the yield-related traits had direct influences on yield per plant (Supplementary Fig. 24). Among the associations with effects on yield, the superior alleles of heading date, plant height and grain number generally had positive effects on yield per plant, while the superior alleles of grain weight generally had negative effects on yield per plant. The flowering time genes (for example, *OsSOC1*, *Ghd8* and *Ghd7*)^35^,^43^,^44^ have the largest effects on grain yield (longer growth stage for higher yield), followed with the genes regulating spikelets (for example, *OsSPL14* and *Gn1*)^34^,^36^,^37^. For the genes underlying grain size and weight^30^,^45^,^46^, the alleles of *GS3* and *GW2* that increase grain weight represented significantly lower grain yield, while the alleles of *qSW5* that increase both the grain weight and yield brought higher degree of chalkiness in rice grains. We further analysed the effects of grain quality-associated loci on the yield per plant. As expected, the superior alleles of grain quality generally had negative effects on the yield per plant. For the GWAS loci underlying chalky grain rate, lower chalky grain rate was generally accompanied with lower grain yield (Supplementary Table 15). Therefore, it needs to make a tradeoff between grain yield and other factors (for example, grain quality, growth duration and disease resistance) in breeding.

Grain yield is a function of various components and factors under different complex agro-ecosystems. We found phenotypic changes between the traits in Sanya and those in Hangzhou (Supplementary Table 16). Among them, plant height and grain number fell over 20% in Sanya as compared with those in Hangzhou. In the hybrid rice panel, we observed that many associations (even with very strong signals) in one location showed weak or no associations in the other location. GWAS for heading date can serve as a typical example (Fig. 3a and Supplementary Fig. 16a). Through comparison of GWAS results between Sanya and Hangzhou, *OsSOC1* and *Ghd7* was significantly associated with heading date variation in both Sanya and Hangzhou. The two loci *Ehd1* and *Hd1* that were identified in Sanya showed relatively weak association signals in Hangzhou, while *Hd3a* and *Ghd8* (controlling plant height as well) with weak associations in Sanya had strong associations in Hangzhou. Similar phenomenon was observed for plant height and grain number. The results suggest that these genes and loci were under the strong regulation of photoperiod and temperature conditions. GWAS result of grain weight was an exception to those findings, for which the major loci (for example, *GS3*, *GW2* and *qSW5*) played their roles in both Sanya and Hangzhou consistently.

### Genomic landscapes of heterosis in hybrid rice population

The exploitation of hybrid vigour in rice has been focusing on increasing grain yield. This might be the reason why most of the available rice hybrids do not possess better grain quality and disease resistance than the inbred lines. It should be noted that, to collect all the parental varieties of the 1,495 hybrid varieties is impractical, and many maternal lines of the hybrid varieties are of low fertility, which can obstruct phenotyping works. Nevertheless, there were parental homozygous genotypes for most loci in the hybrids themselves, which enabled the analyses of the heterotic loci and the dominance/overdominance effects. We evaluated the effect of heterozygous loci for the GWAS peaks above the suggestive *P* value (*P*<10^−4^, from the linear mixed model) underlying yield traits (yield per plant, panicle number, grain number per panicle and plant height) in Sanya and Hangzhou. We found that most loci showed incomplete dominance effects. There were 13 sites with strong overdominance effects (10 with positive effect and 3 with negative effect; Supplementary Table 17). For example, the hybrid rice varieties with heterozygous alleles at a GWAS loci on chromosome 6 (*Hd3a* is located within the local region) represented better performance than both the homozygous genotypes in plant height, flag leaf length and yield per plant in Hangzhou (but not in Sanya). The locus is also one of the highly differentiated loci between the parental lines (Fig. 2). These overdominance loci should play an important role in increasing the yield of rice hybrids, which, however, can only explain a part of heterosis. The overdominance loci are much fewer than those with incomplete dominance in number (Supplementary Fig. 25), which implies that dominance is an important contributor to heterosis in hybrid breeding.

We then analysed the three traits in a genome wide manner—grain number, plant height and heading date in Sanya. For each trait, we focused on the peak SNPs at the top 100 associated loci (ranked in GWAS *P* value). We compared the effects of heterozygous and homozygous genotypes at the associated loci. We found that, of most loci for grain number, the average effects of the heterozygous genotypes exceeded the average effects of homozygous genotypes in hybrids, suggesting more positive dominance effects overall. The case of heading date was the opposite. The degree of plant height fell in between those of grain number and heading date (Supplementary Fig. 26). The overall effects of the heterozygous genotypes in hybrids are consistent with the corresponding heterosis degree in the parent–hybrid trios (Supplementary Figs 27 and 28). According to the phenotypic data of the parent–hybrid trios in Sanya, grain number generally showed a high level of positive heterosis in most hybrids; plant height had a modest level; and heading date represented a high level of negative heterosis. Similar phenomenon was observed for yield per plant, for which the overall heterosis in Sanya was stronger than that in Hangzhou (Supplementary Figs 29–31). The result indicates that the occurrence of heterosis is dependent on the overall net effects of the positive dominance versus the negative dominance of numerous loci throughout the whole genome.

We investigated the frequency of superior alleles of the associated loci in both the hybrids and the parental lines. Surprisingly, the superior gene alleles of grain number were found to be mostly of low frequency at the population scale (Supplementary Fig. 32). For example, the superior allele frequency of *NAL1* and *OsSPL14* (both controlling grain number) was 5 and 2%, respectively. For the sites with rare alleles, the heterozygous genotypes in the hybrid rice varieties would be approximately twofold of homozygous superior genotypes in the inbred lines according to the HWE (2*(1−*q*)*q/*q*≈2 when *q* is very small; *q*: minor allele frequency), if considering that nearly all the gene alleles of the F_1_ offspring are inherited from their parental inbred lines (Supplementary Fig. 33). Since many heterozygous alleles would have positive dominance effects, hybrid breeding results in a rapid increase in the superior genotypes, which is probably the reason why hybrid breeding is more effective than the breeding of inbred rice varieties.

**Figure 4:**
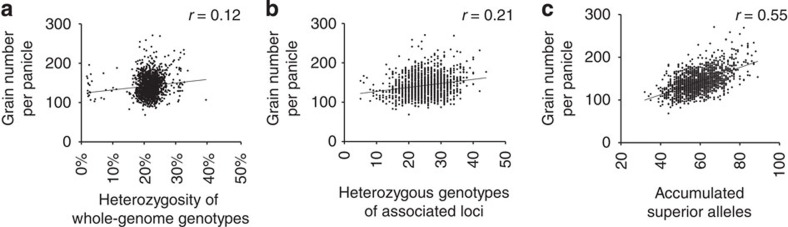
The relationships between superior alleles and phenotypic variation of the yield performance. We used the peak SNPs at the top 100 associated loci (ranked in GWAS *P* value for grain number in Sanya) to calculate heterozygous genotypes and accumulated superior alleles of each *indica* hybrid. (**a**) The correlation between heterozygosity of whole-genome genotypes in each hybrid variety and the phenotypic performances of grain number; (**b**) the correlation between the numbers of heterozygous superior genotypes and grain number; (**c**) the correlation between the numbers of the totally accumulated superior gene alleles and grain number.

We investigated the relationships between heterozygosity and phenotypic variation and found very weak correlations between the heterozygosity of whole-genome genotypes and the yield traits, which was consistent with the observations in the linkage mapping populations^16^,^17^, suggesting that the overall heterozygosity made little contribution to heterosis. We further turned to the assessment of the cumulative effects of superior genotypes (Fig. 4 and Supplementary Figs 34 and 35). There were only weak or modest correlations between the number of heterozygous superior genotypes in each hybrid and its phenotypic performances. In contrast, there were high correlations between the phenotypic variations of the traits and the number of accumulated superior gene alleles. We noticed that nearly all inbred parental lines and hybrid rice varieties contained less than half of total superior gene alleles for grain number and plant height (Supplementary Fig. 36), indicating that mining and utilization of more superior alleles have the potential to contribute to the further yield enhancement. It should be effective to pyramid multiple superior alleles into a single inbred line with ideal genotypes, but it would be still of enormous difficulties, which would rely on either using very large recombinant population or undergoing many cross-selection cycles according to the computational simulations (Supplementary Fig. 37). Generations of numerous F_1_ crosses and selections from the hybrid combinations with strong heterosis are still one of the most rapid and efficient ways in breeding.

## Discussion

In this study, we generated the largest-scale genomic and phenomic data sets up to now for hybrid rice and parental lines that represent the world’s most elite and superior rice varieties^4^. We have analysed the highly heterozygous genomes and the genetic effects of heterozygous alleles on heterosis, and found that effective pyramiding of rare superior alleles with positive dominance in hybrids could result in the heterotic phenomenon. This genomic investigation, using a large number of rice hybrids, together with previous analyses using experimental populations from *indica*–*japonica* hybrid^16^ or *indica*–*indica* hybrid^17^,^18^, implies the leading role of dominance complementation in heterosis.

At the genome scale, the overall effect of heterozygous genotypes may still be inferior to homozygous superior alleles, suggesting that it would be possible to generate higher-yielding inbred lines without the need to construct hybrids by crossing the inbred parental lines. However, considering the complex nature of yield, it would not be certain that the superior alleles all give additive benefit when combined together because of the likelihood of epistatic interactions. Genomic prediction for yield performance would greatly await better understanding of genetic interaction network, based on which the epistasis effects could be well incorporated into computation modelling^19^,^47^. Our work provides the information on numerous superior alleles for yield traits and could be a start for future in-depth studies on epistatic and genomic prediction modelling in rice. Moreover, we worked with elite hybrids directly in this study, although the frequency of different parental lines used in the hybrid combinations may complicate allele frequencies across the genome. There has been limited research into genetic analyses of highly heterozygous genomes in plants. The study on hybrid rice opens an area or research that deserves more attention in future, including the generation of more suitable genetic populations and the development of more powerful statistical analysis frameworks.

Grain yield is a very complicated trait and in this study its major components were analysed. There are too many genetic and non-genetic factors for grain yield and genetic dissection and heterosis analysis directly on grain yield (for example, yield per unit) is challenging for such a large number of hybrid crosses. Follow-up works that focus on a subset of crosses may increase the power of genetic mapping for yield. Construction and detailed phenotyping of populations from some specific crosses, along with applications of large-scale variant discovery and genotyping, which now become more and more practical and economical^24^,^25^,^26^,^48^,^49^, might facilitate cloning of quantitative trait locus underlying grain yield^50^.

The hybrids from *indica*–*japonica* cross represented outstanding performances^16^ in grain number in both Sanya and Hangzhou conditions, which may benefit from the introduction of superior alleles between subspecies (for example, *NAL1*, fixed in *japonica* but rare in *indica*). Increasing diversity by *indica*–*japonica* or cultivated wild crosses, which could introduce more low-frequency superior alleles promptly, would be major concerns for hybrid rice breeding in the future.

## Methods

### Sampling and sequencing

Both the hybrid varieties and the inbred parental lines were from the collections of rice accessions preserved at the China National Rice Research Institute in Hangzhou, China. The hybrid rice varieties were sent to the National Crop Variety Approval Committee for extensive evaluations to ensure that the varieties are genetically unique and suitable for wide agricultural applications. The rice varieties are also publically available in seed markets. No protections of genotyping, sequencing and genetic analyses were involved for the rice varieties. The hybrid cultivar panel consisted of 1,495 lines. In total, 1,170 lines were generated from the three-line system (cytoplasmic male sterility), and 325 lines were bred from the two-line system (photoperiod- and thermo-sensitive genic male sterility). Among the hybrids, 1,247 lines were the major cultivated hybrids in the 1990s and 2000s, and the rest were developed recently and entered the national hybrid variety application tests. The inbred parent panel consisted of 90 lines, consisting of 38 female parental lines (male sterile lines or maintainer lines) and 52 male parental lines (restorer lines). The data set of 1,495 hybrid rice varieties for GWAS is available at the Rice Haplotype Map Project database ( http://www.ncgr.ac.cn/RiceHap4).

For each accession, its genomic DNA was prepared from a single plant for sequencing, and the sequencing library was constructed with an insert size of 200–400 bp for the paired-end reads. The genomes of 1,495 hybrid varieties were sequenced on the Illumina HiSeq2000 at twofold genome coverage, generating 96-bp paired-end reads. Four hybrid rice varieties out of the 1,495 hybrids were sequenced individually at a deep-genome coverage ranging from 38x to 42x, generating 97-bp paired-end reads. The amplification-free method for library preparation^51^ was used in deep sequencing of the four hybrid rice varieties, which reduced the incidence of duplicate sequences thus facilitating heterozygous genotype identification. The 90 inbred parental lines were sequenced on Illumina Genome Analyzer IIx at ~15x genome coverage, generating 76-bp paired-end reads. Library construction and sequencing of these rice accessions were performed according to the manufacturer’s protocol of Illumina.

The detailed information of the rice varieties is listed in Supplementary Data 1 and Supplementary Table 2.

### Read alignment and imputation

The paired-end reads of all the hybrid rice accessions were aligned against the rice reference genome^52^ (IRGSP 4.0) using the software Smalt (version 0.5.7, http://www.sanger.ac.uk/resources/software/smalt/) with the parameters of ‘-i 700 -j 50’ and ‘-m 50’. Only the reads mapped uniquely to the rice genome sequence were retained for further analysis. Read alignment results were sorted according to their mapping positions on the chromosomes and the raw genotype information was listed using the Ssaha Pileup package (version 0.8) for each covered nucleotide in each of the 1,495 hybrid rice varieties. Genotypes and their corresponding frequencies were further called and recorded at all the SNP sites from the Ssaha Pileup outputs. In total, 1,654,030 SNPs were called after elimination of all the rare SNPs and the SNPs in the highly polymorphic regions.

The frequencies of the reference allele and the alternative allele were calculated at each SNP site (for example, the two alleles ‘T’ and ‘C’ at one SNP site) for each hybrid rice variety, and the binomial model was used to calculate the genotype likelihood of the three kinds of genotype (that is, ‘TT’, ‘TC’ and ‘CC’). The data was converted into the BEAGLE (version 3.3.2) input file to impute the genotype. After the first round of the data imputation, the outputs were used as the reference panel to carry out the second round of data imputation. After the phasing iteration, the resulting genotype data set was used in the followed analyses. The specificity of the genotype data was checked using high-coverage sequencing data of both the hybrid rice varieties and inbred parents. In total, four deeply sequenced hybrids and 35 parent–hybrid trios were used to assess the accuracy of the imputed genotype data sets, and the results are listed in Supplementary Table 1 and Supplementary Fig. 2. We used 1,253,232 SNPs (minor allele frequency >0.03 in 1,439 *indica* hybrids) for GWAS and heterosis analysis.

### Analysis of parental line haplotypes

We designed a computational method to carry out whole-genome haplotype construction for the hybrids, with the aid of the genome sequences of 90 commonly used inbred parents. We proposed that the pedigree relationship of the hybrids can facilitate the inference of the parental lines’ haplotypes. For example, the hybrid Teyou86 (one rice hybrid variety in our collection) was from a cross between Minghui86 (the male parental line, one of the sequenced 90 inbred lines) and LongtefuA (the female parental line, neither the seed nor its DNA were sampled in our collection). Since the genome data of both the F_1_ (Teyou86) and the male parent (Minghui86) was already available, the haplotype of LongtefuA could be inferred directly and accurately. Furthermore, LongtefuA was the female parental line of 51 hybrids in our hybrid rice panel. Hence, the male parental lines of the remaining 50 hybrids could be inferred subsequently. Considering that, we developed a multiple-iteration approach for whole-genome haplotype reconstruction.

We calculated the kinship value between the full-imputed genotypes of each hybrid and the genotypes of each inbred parental line (*n*=90 in the initial running round). For each hybrid, the inbred line with the highest kinship value was selected as ‘the candidate parent’, unless none of the inbred parental lines in the collection were found to show a close kinship with the hybrid’s genotypes. In haplotype reconstruction, the phased genotype of the hybrid and the haplotype of ‘the candidate parent’ were compared to infer the haplotype of the other parent. For the SNP sites where the genotype of ‘the candidate parent’ was missing (or it was contradictory with that in the hybrid genome), we used the phasing information of the nearest SNPs for local haplotyping. After the first running round, the haplotypes of all the inferred parents were collected together to generate a new panel of inbred parental line. The second running round was continued by comparing the 1,495 hybrids with the new inbred parental line panel (*n*=225 in the second running round). A total of four iterations were carried out and finally the parent’s information of 1,361 hybrids (91% of our collection) was retrieved. The deduced parental genotypes were estimated to be in agreement with the real genotypes at >98.0% of the total SNP sites. The computational analysis for the haplotype reconstruction was only based on genome data, where no pedigree records were used.

### Population genetics analysis

The individual ancestries were estimated from whole-genome SNPs using the software ADMIXTURE (version 1.23)^53^. The matrix of pair-wise genetic distance derived from simple SNP matching coefficients was used to construct phylogenetic trees using the software PHYLIP51 (version 3.66)^54^. The software MEGA5 was used for visualizing the phylogenetic trees^55^. Principal-component analysis of the SNPs was performed using the software EIGENSOFT (version 5.0.1)^56^. The sequence diversity statistics (*π*) were computed in each 100-kb window of the rice genome. In the analysis of selections in modern breeding, the value of *π* was calculated for modern hybrids (*π*_hybrid_) and traditional landraces (*π*_landrace_) in *indica* and *japonica*, respectively, and the ratio of *π* in the population of landraces to that in the population of hybrids (*π*_landrace_/*π*_hybrid_) was used to detect the genetic-improvement sweeps. The genomic regions where both the hybrids and the landraces showed a low level of genetic diversity or the regions that had too many missing data and repetitive sequences were excluded for further analysis.

The frequencies of ‘the homozygous genotypes of both the reference alleles’ (rr), ‘the homozygous genotypes of both the alternative alleles’ (aa) and the ‘heterozygous genotypes’ (ra) in the 1,439 *indica* hybrids were counted at each SNP site, from which we computed the values of the H_o_ and minor allele frequency. The H_e_ was calculated by the HWE at each SNP site. HWE tests were performed by the ‘genetics’ package in R language. The heterozygosity of each hybrid rice variety was measured as the proportion of the heterozygous genotypes at all the SNP sites.

### Phenotyping and GWAS

Approximately 18 seeds for each variety from the collection of hybrids were germinated and planted in the experimental fields in Hangzhou, China (at N 30.32°, E 120.12°) in summers of 2012 and 2013, and in Sanya, China (at N 18.65°, E 109.80°) in winter of 2012. All the 1,495 accessions were grown in the consecutive farmland with well-distributed soil status and uniform condition. Lands tilling and raking were conducted as even as possible to make equal growing conditions for each accession. The phenotyping for this work involved a wide range of agronomic traits for grain yield, grain quality and disease resistance. The field traits including heading date, plant height, flag leaf length, flag leaf width, panicle number and panicle length, were measured directly in the field. Heading date was recorded daily as the number of days from sowing to the observation of first inflorescences that emerged above the flag leaf sheath. In total, 75 hybrid rice varieties showed extremely late maturing in Hangzhou, which were removed in GWAS for heading date in Hangzhou. Plant height, flag leaf length, flag leaf width and panicle length were measured for at least three samples of each accession. The grain-related traits, including grain number per panicle, grain length, grain width and grain weight per 1,000 grains, were measured in the laboratory following harvest. Grain weight was initially obtained by weighing ~600 fully filled grains, which was then converted to 1,000-grain weight value. For measuring grain quality traits, the fully filled grains from the hybrids planted in Sanya (from winter of 2012 to spring of 2013) were used. Harvested rice grains were air-dried and stored at room temperature for at least 3 months until grain moisture content fell to be <13.5%. The grain quality traits were then phenotyped according to the Chinese national standard (NY/T 2334-2013 ‘Determination of head rice yield, grain shape, chalky rice percentage, degree of chalkness and translucency—an image analysis method’). Amylose content and alkali digestion value of the grains were phenotyped and scored using milled rice grains^57^,^58^. To evaluate the disease-resistance traits of the hybrids, plants were germinated in Hangzhou in the summer of 2013, and incubate by the spraying method in low light and at room temperature to insure sporulation and subsequent reinfection of susceptible plants. The spraying method was carried out for the disease-resistance assay^59^. In brief, rice seedlings were inoculated by spraying fresh preparation of conidial suspension. The inoculated rice varieties were then planted in air-conditioned greenhouses, followed by the phenotyping of disease-resistance traits. The disease reactions were measured about 7 days after inoculation and evaluated and scored by the disease leaf area.

Association analysis was conducted using the EMMAX software package. The matrix of pair-wise genetic distance derived from simple matching coefficients of SNPs was used to model the variance–covariance matrix of the random effect. Permutation tests were used to help define the genome-wide significant *P* value threshold^60^. We picked 10 traits, reshuffled the original phenotype data, and then performed association analysis using EMMAX with the same parameters. There ought to be no real associations between the SNPs and the ‘simulated’ phenotypes, so all the SNPs passing the threshold should be false positives. A total of 100 permutation analyses were performed, which detected three ‘association signals’ passing the whole-genome significant cutoff 10^−6^. GWAS on 38 real phenotypes identified a total of 130 association signals passing the threshold 10^−6^, which suggested a feasible FDR level of <0.01. Moreover, FDR value was calculated to be 0.2 for the suggestive *P* value of 10^−4^ according to the permutation tests. Additive, dominant and recessive models were also tested for the traits. The EMMAX software package simply follows the encoding scheme of the genotype data in the additive model by default. For dominant and recessive models, heterozygous genotypes (ra) were changed to be ‘homozygous genotypes of both the reference alleles’ (rr) or ‘homozygous genotypes of both the alternative alleles’ (aa) for all the SNPs. The newly made genotype data was then inputted into the EMMAX software package for GWAS.

### Heterosis analysis in rice hybrids

For the parent–child trios, phenotypic performances (heading date, plant height, grain number per panicle and yield per plant) of both the F_1_ hybrids and their parental lines were used to evaluate the amount of heterosis (middle parent heterosis and over parent heterosis). In each parent–child trio, the middle parent heterosis index was calculated using the phenotypic measurements of the F_1_ hybrid and both the parents. Moreover, the parent–child trios could be divided into three types: positive over parent heterosis, negative over parent heterosis and F_1_ ranging between the parents. The proportions of the three types were counted as well to evaluate the amount of heterosis in the hybrid rice population.

The degree of dominance ‘*d*/*a*’ was calculated using the peak SNPs of the associated loci, where ‘*d*’ and ‘*a*’ referred to the dominant effect and the additive effect, respectively. The effects of heterozygous alleles were analysed for the GWAS peaks above the suggestive *P* value (*P*<10^−4^, from the linear mixed model) underlying the yield per plant, panicle number, grain number and plant height. The SNP sites in which heterozygous genotypes or homozygous genotypes of both the minor alleles had a frequency of ≤15 in number were excluded in the calculation of ‘*d*/*a*’. The effects of heterozygous and homozygous genotypes were calculated for each peak SNP of the associated locus, where the average phenotypic measurements of the heterozygous genotypes and homozygous genotypes were calculated, respectively.

Associated loci were screened in each 500-kb window of the rice genome, for the traits heading date, plant height and grain number per panicle in Sanya. The lowest GWAS *P* value (from the linear mixed model) of the SNPs in each 500-kb genomic region was recorded as the association signal of the loci. The peak SNPs at the top 100 associated loci (ranked in association signals) were used in the analysis. For each associated locus, the allele with better yield performance (for example, more grain numbers, higher plant and longer heading date) was defined as the superior gene allele.

The computational simulations under the two scenarios were performed using the sim.map and sim.cross functions in the R/Bioconductor package, with recombination under the real genetic map in rice. In the first scenario, we generated *in silico* genotype data of 5,000 recombinant inbred lines. In the second scenario, we generated *in silico* genotype data of 500 BC_5_F_3_ lines (Supplementary Fig. 37).

## Author contributions

B.H. conceived the project and its components. X.H. and B.H. designed the studies and contributed to the original concept of the project. S.Y. and J.G. contributed to the collection of hybrid rice. J.G., Qilin Z., B.C., J.X., N.C., Z.H. and S.Y. contributed in the phenotyping of the hybrid rice. W.L., Y.L., C.Z., D.F., Q.W. and Q.F. performed the genome sequencing. X.H., Y.Z., H.G. K.L., C.Z., T.H., L.Z. and Q.Z. performed the genome data analysis. X.H., Y.Z. and H.G performed GWAS, population genetics and statistical analyses. J.L. and Z.-X.W. contributed to the functional analyses. X.H. and B.H. analysed the whole data and wrote the paper.

## Additional information

**Accession codes:** DNA sequencing data are deposited in the European Nucleotide Archive ( http://www.ebi.ac.uk/ena/) under accession numbers ERP005527.

**How to cite this article:** Huang, X. *et al.* Genomic analysis of hybrid rice varieties reveals numerous superior alleles that contribute to heterosis. *Nat. Commun.* 6:6258 doi: 10.1038/ncomms7258 (2015).

## Supplementary Material

Supplementary Information Supplementary Figures 1-37 and Supplementary Tables 1-17.

Supplementary Dataset 1 The list of 1495 hybrid rice varieties sampled in the study

Supplementary Dataset 2 Phenotype data measured in Sanya

Supplementary Dataset 3 Phenotype data measured in Hangzhou
