# Supplementary material for: Genomic analysis of hybrid rice varieties reveals numerous superior alleles that contribute to heterosis
Source: Nat Commun. 2015 Feb 5;6:6258. doi: 10.1038/ncomms7258 (PMC4327311; doi:10.1038/ncomms7258)
Supplement: Supplementary Information — Supplementary Figures 1-37 and Supplementary Tables 1-17. [file ncomms7258-s1.pdf]

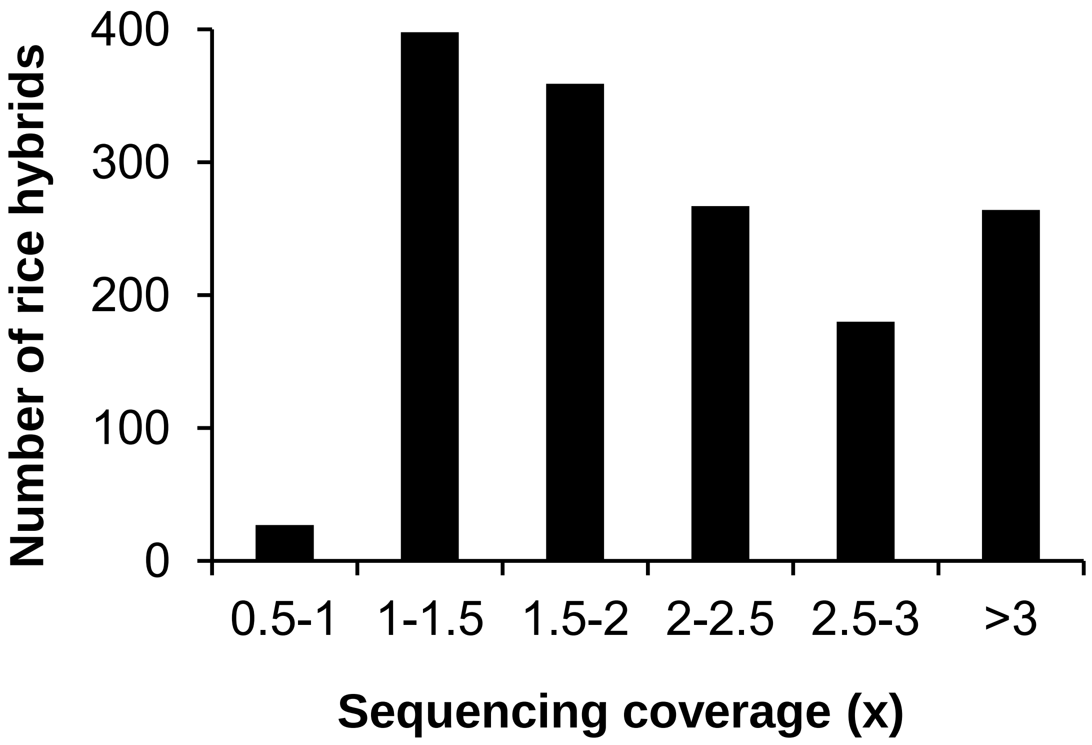

Supplementary Figure 1 Sequencing coverage of 1495 rice hybrids. The genomes of 1495 hybrid varieties were sequenced on the Illumina HiSeq2000 platform. An average of two-fold genome coverage of 2x96bp Illumina reads was generated for each hybrid sample.

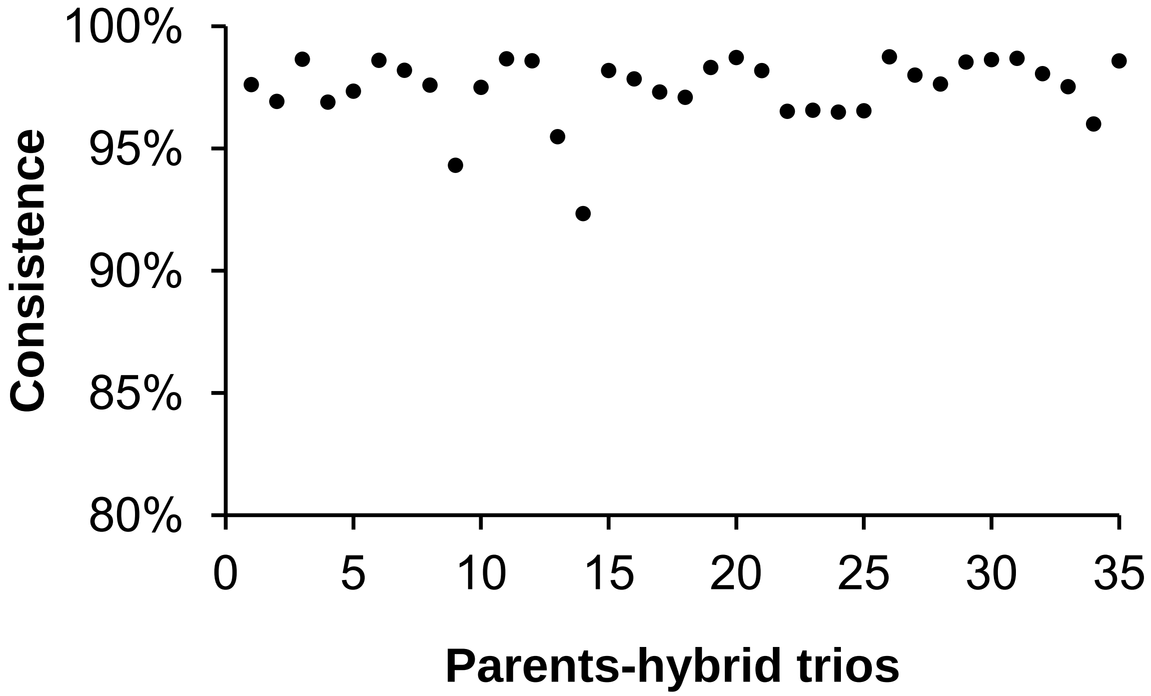

Supplementary Figure 2 Specificities of the genotype dataset of the rice hybrids estimated by parents-hybrid trios. There are totally 35 parents-child trios, in which the F_1 hybrids and their both parents were sampled and sequenced. The experimentally determined haplotypes (genotype calls directly from sequence data) from both parents were compared with the imputed genotypes of the corresponding F_1 hybrids (genotypes derived from the imputation pipeline Beagle), by which the consistence was evaluated for each parents-hybrid trio.

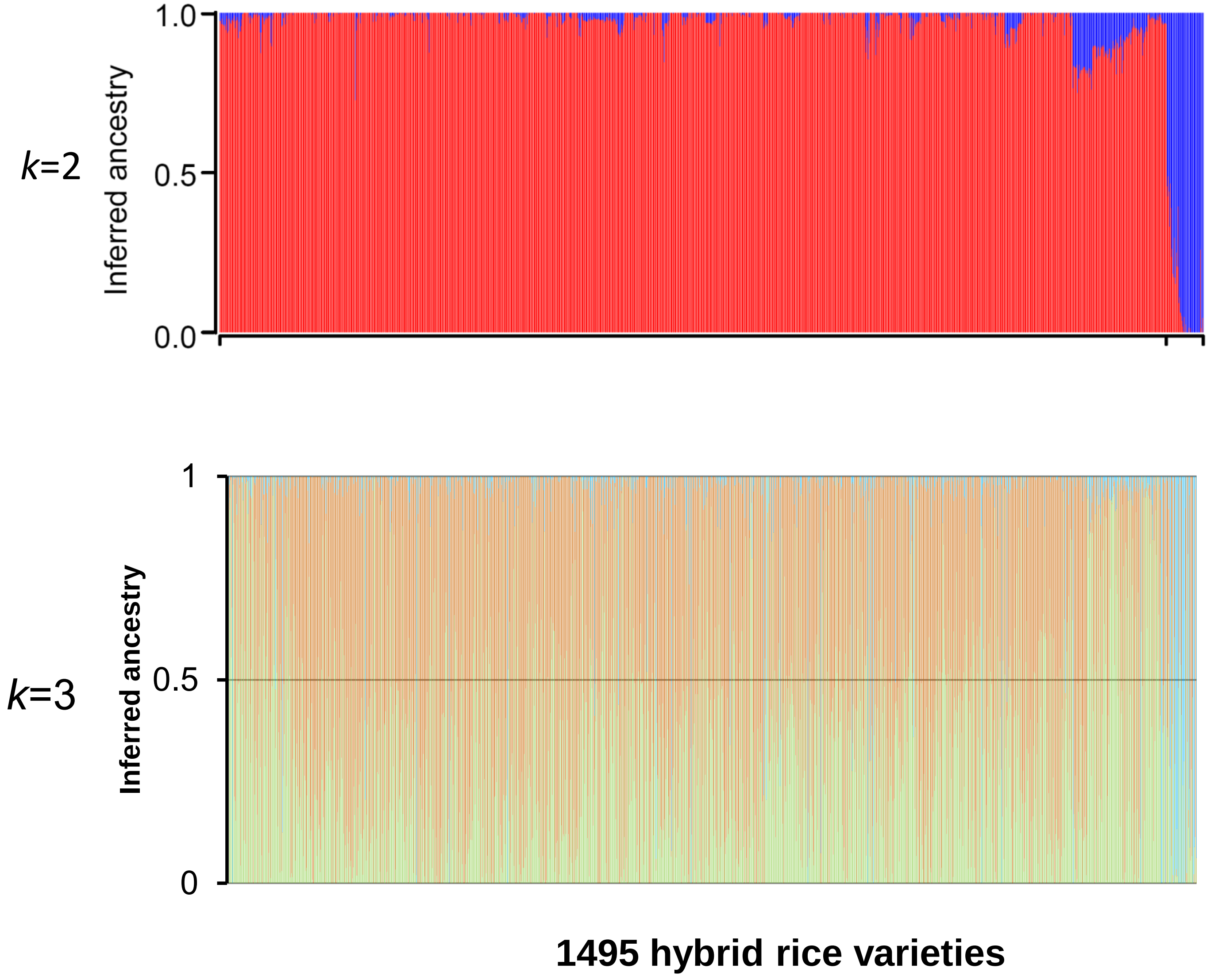

Supplementary Figure 3 Global admixture analysis of 1495 rice hybrids at K=2 and K=3. The majority of rice hybrid varieties were from *indica-indica* crosses, with a small number of hybrids from *indica-japonica* or *japonica-japonica* crosses. The individual ancestries were inferred from whole-genome SNP data using the software ADMIXTURE (version 1.23). In K=2, the genetic background of *indica* subspecies was colored in red, while that of *japonica* subspecies was indicated in blue.

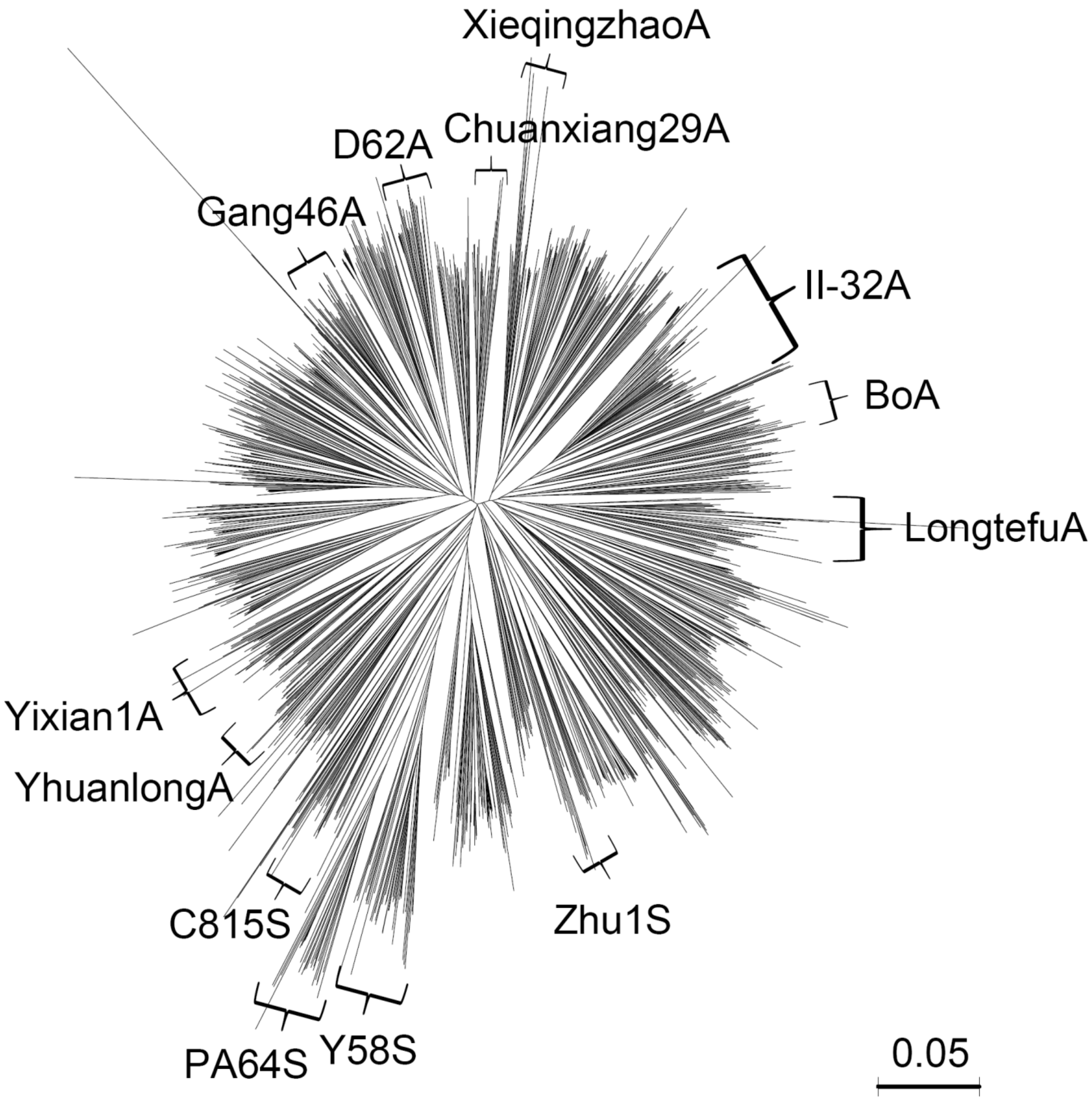

Supplementary Figure 4 Neighbor-joining tree of 1439 *indica* hybrids. The bar indicates the simple matching distance of all SNPs. The hybrid varieties derived from the same maternal lines are clustered in the same clades, and the name of the male sterile lines were labeled (A: cytoplasmic male sterility used in three-lines system; S: environmentally sensitive genetic male sterility used in two-lines system).

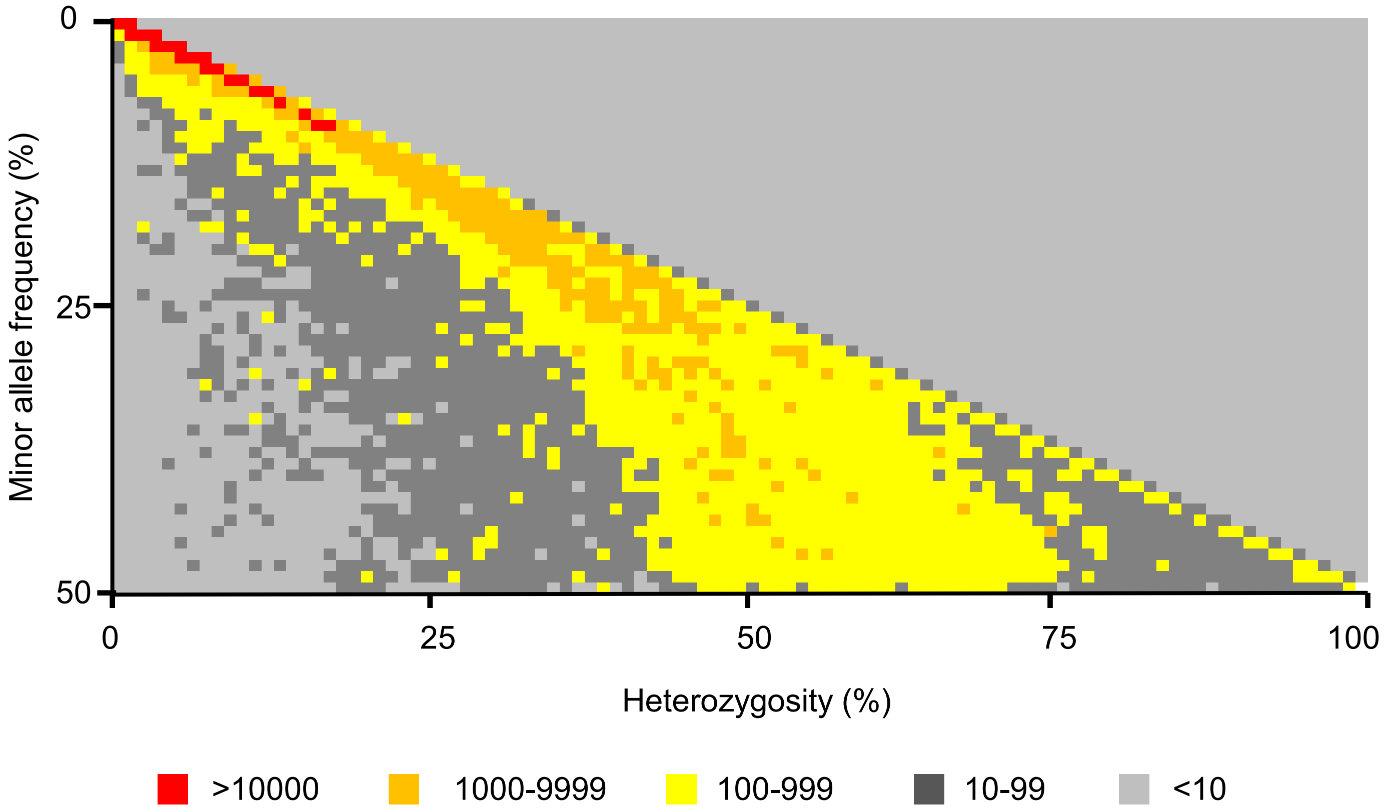

Supplementary Figure 5 The distribution of allele frequency and heterozygosity in the population of 1439 *indica* hybrids. For each SNP, minor allele frequency (MAF) and the frequency of heterozygous genotypes in the population were calculated. All SNPs were divided into 5,000 sets, where each set has a given level of minor allele frequency (50 classes: 0%-1%, 1%-2% to 49%-50%) and heterozygosity (100 classes: 0%-1%, 1%-2% to 99%-100%). Color index indicates the number of SNPs within each set of SNPs.

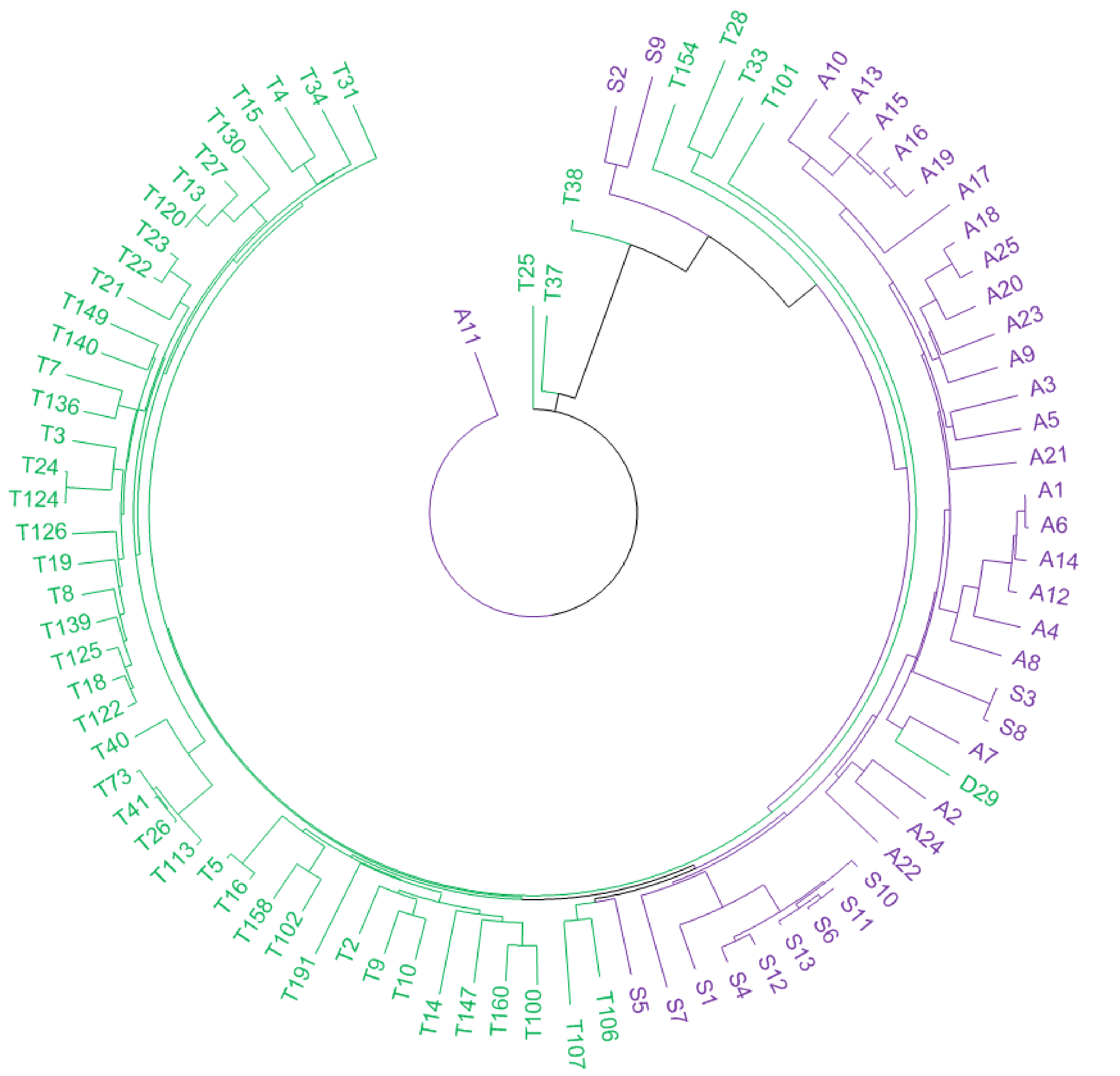

Supplementary Figure 6 Phylogenetic tree of 90 sequenced inbred lines. The male sterile lines and the restorer lines are colored in purple and green, respectively. For the male sterile lines, “A” indicates cytoplasmic male sterility used in three-lines system, while “S” indicates environmentally sensitive genetic male sterility used in two-lines system.

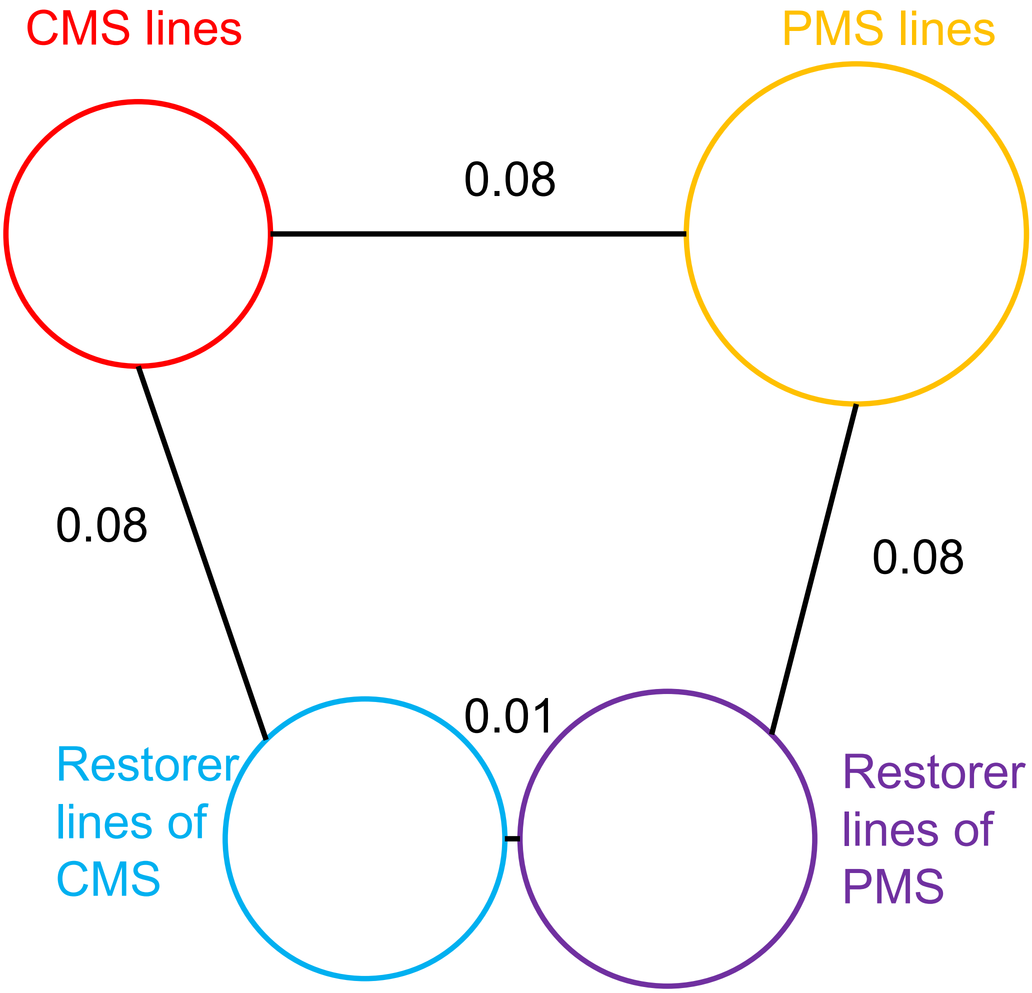

Supplementary Figure 7 Illustration of genetic diversity and population differentiation for the male sterile lines and the restorer lines. The sizes of the circle represent the level of genetic diversity (π) of the groups, and the values of genetic differentiation (F_{st}) between the groups are indicated. CMS lines indicate cytoplasmic male sterility used in three-lines system, while PMS lines indicate environmentally sensitive genetic male sterility used in two-lines system.

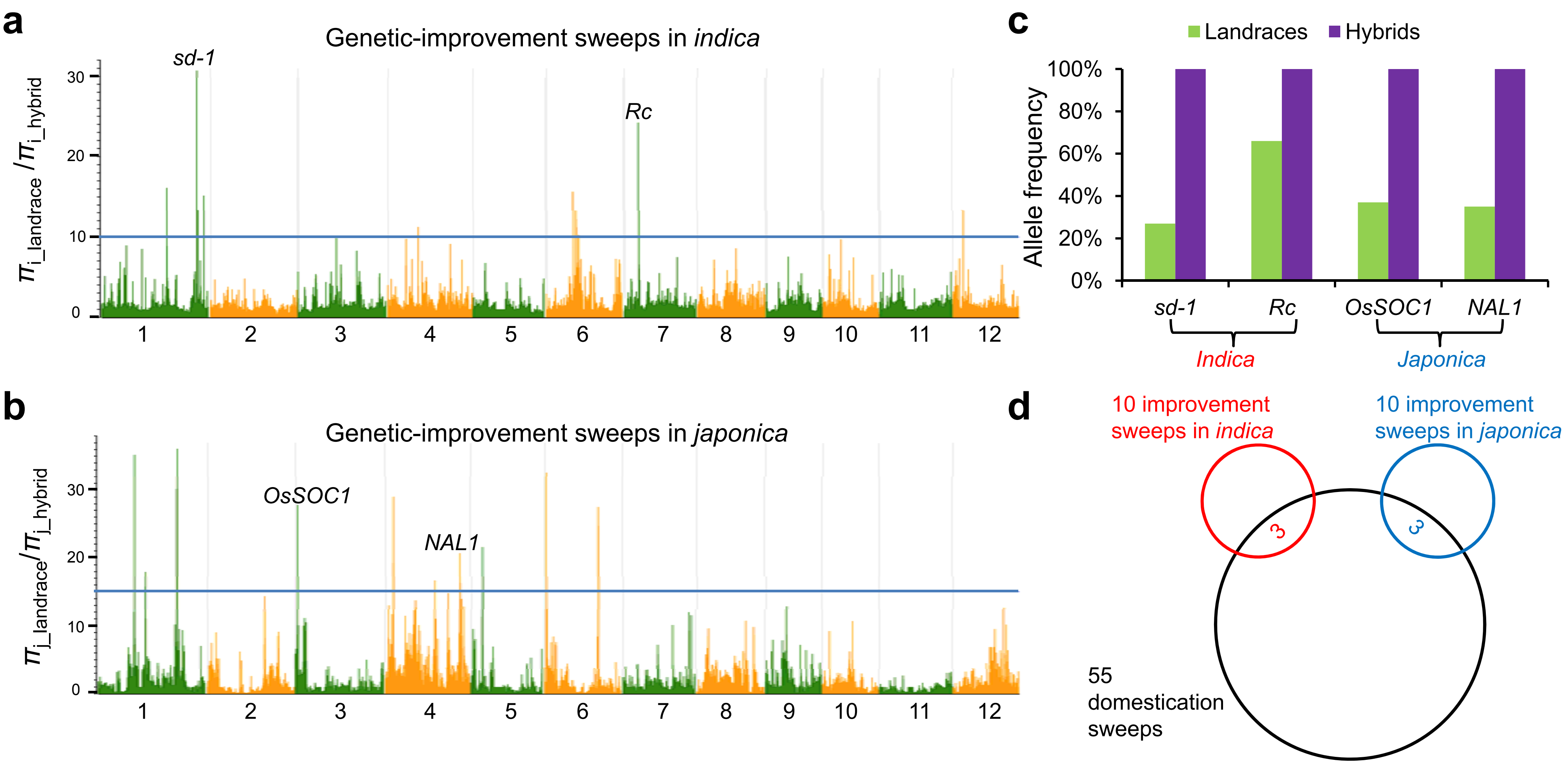

Supplementary Figure 8 Whole-genome screening of genetic-improvement sweep in modern breeding. (**a**) Genome-wide selection signals in the population of *indica* hybrid varieties. The values of $\pi_{i\_landrace}/\pi_{i\_hybrid}$ are plotted against the position on each chromosome. The horizontal line indicates the genome-wide threshold of selection signals ($\pi_{i\_landrace}/\pi_{i\_hybrid} > 10$). (**b**) Genome-wide selection signals in the population of *japonica* hybrid varieties. The horizontal line indicates the threshold of selection signals ($\pi_{j\_landrace}/\pi_{j\_hybrid} > 15$). (**c**) The spectrum of the frequency of desired alleles at *sd-1*, *Rc*, *OsSOC1* and *NAL1* in traditional landraces and modern hybrids. (**d**) The Venn diagram of selective sweeps in domestication and modern breeding. The 55 domestication sweeps were identified in a previous study. The numbers of overlaps between the selective sweeps are indicated.

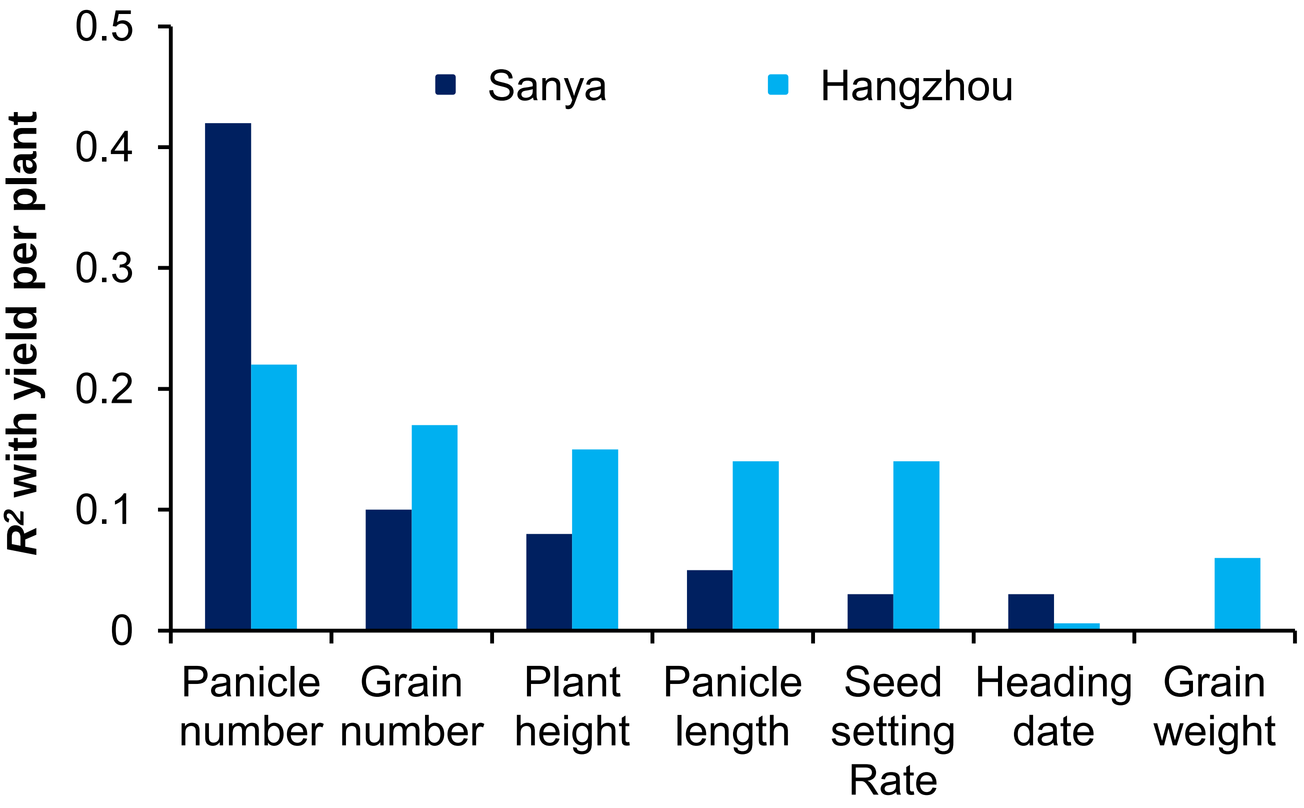

Supplementary Figure 9 Contributions of different yield-related traits to yield per plant in Sanya and Hangzhou. Pearson correlation coefficient (R^2) was calculated between each yield component or related trait (X) and yield per plant (Y) in Sanya and in Hangzhou, respectively.

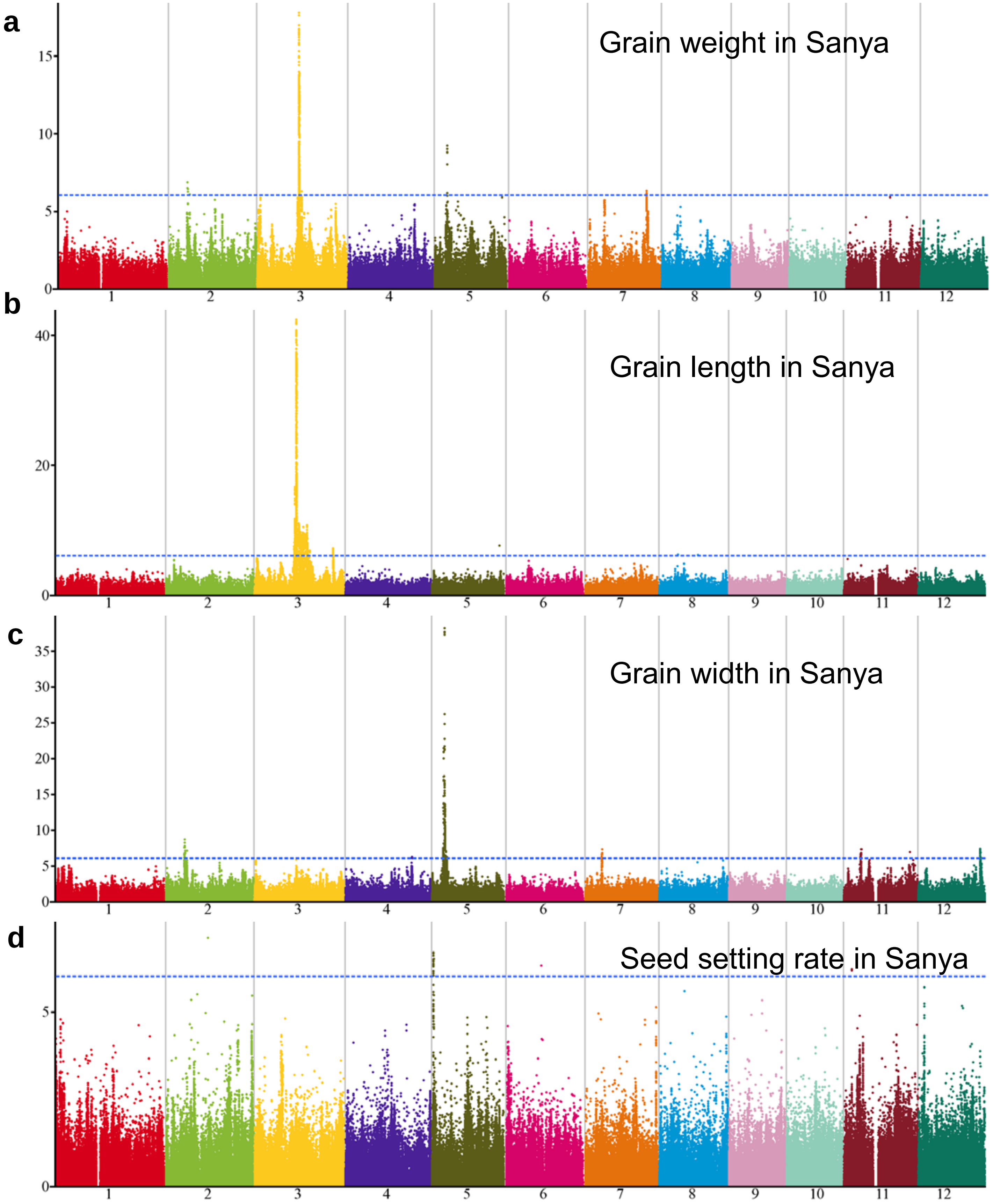

Supplementary Figure 10 GWAS of grain weight, grain length, grain width and seed setting rate in Sanya. Negative $\log_{10}$ *P*-values from linear mixed model (Y-axis) are plotted against SNP positions (X-axis) on each of 12 chromosomes. This Manhattan plot is the result from additive model.

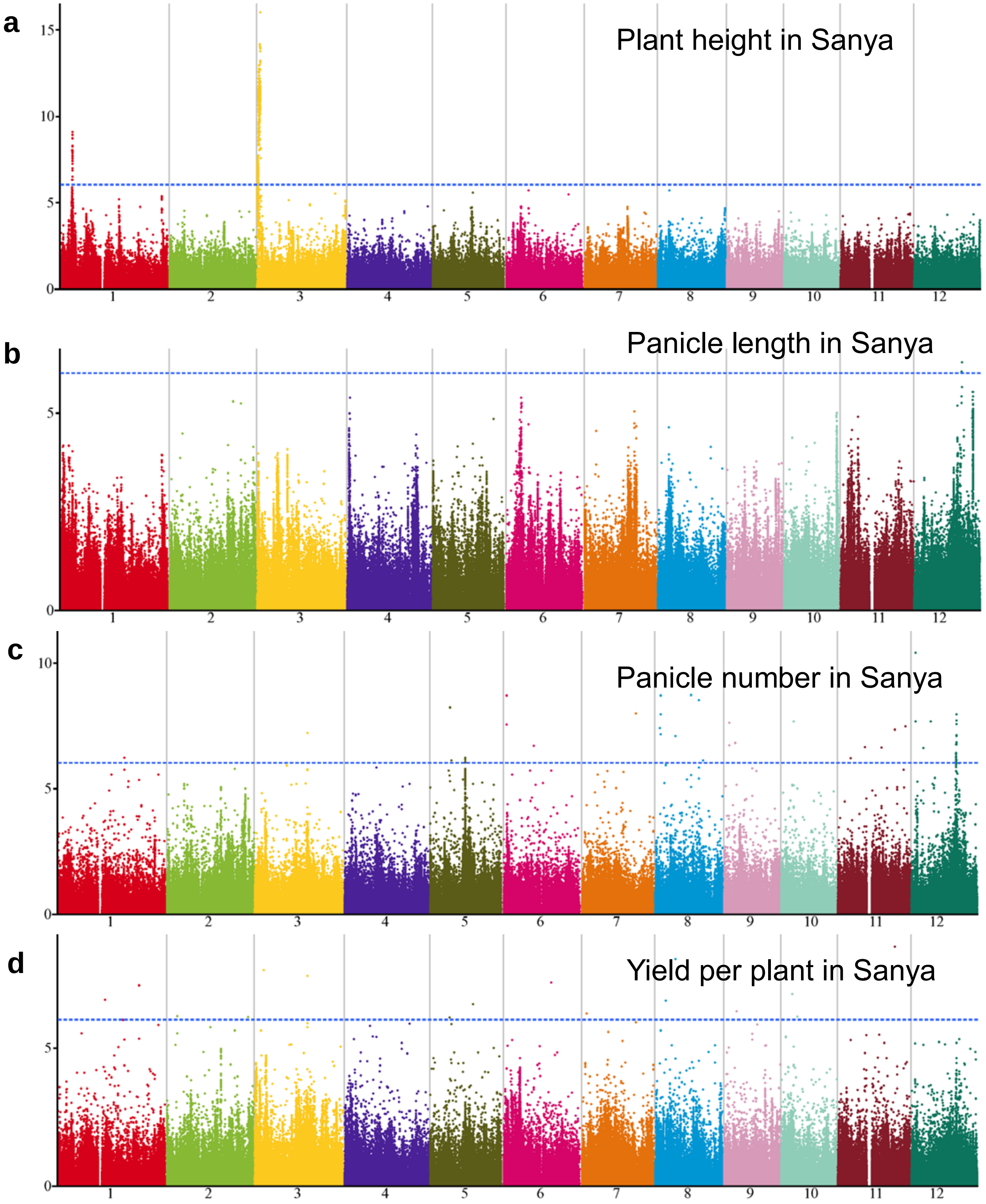

Supplementary Figure 11 GWAS of plant height, panicle length, panicle number and yield per plant in Sanya. Negative $\log_{10}$ *P*-values from linear mixed model (Y-axis) are plotted against SNP positions (X-axis) on each of 12 chromosomes. This Manhattan plot is the result from additive model.

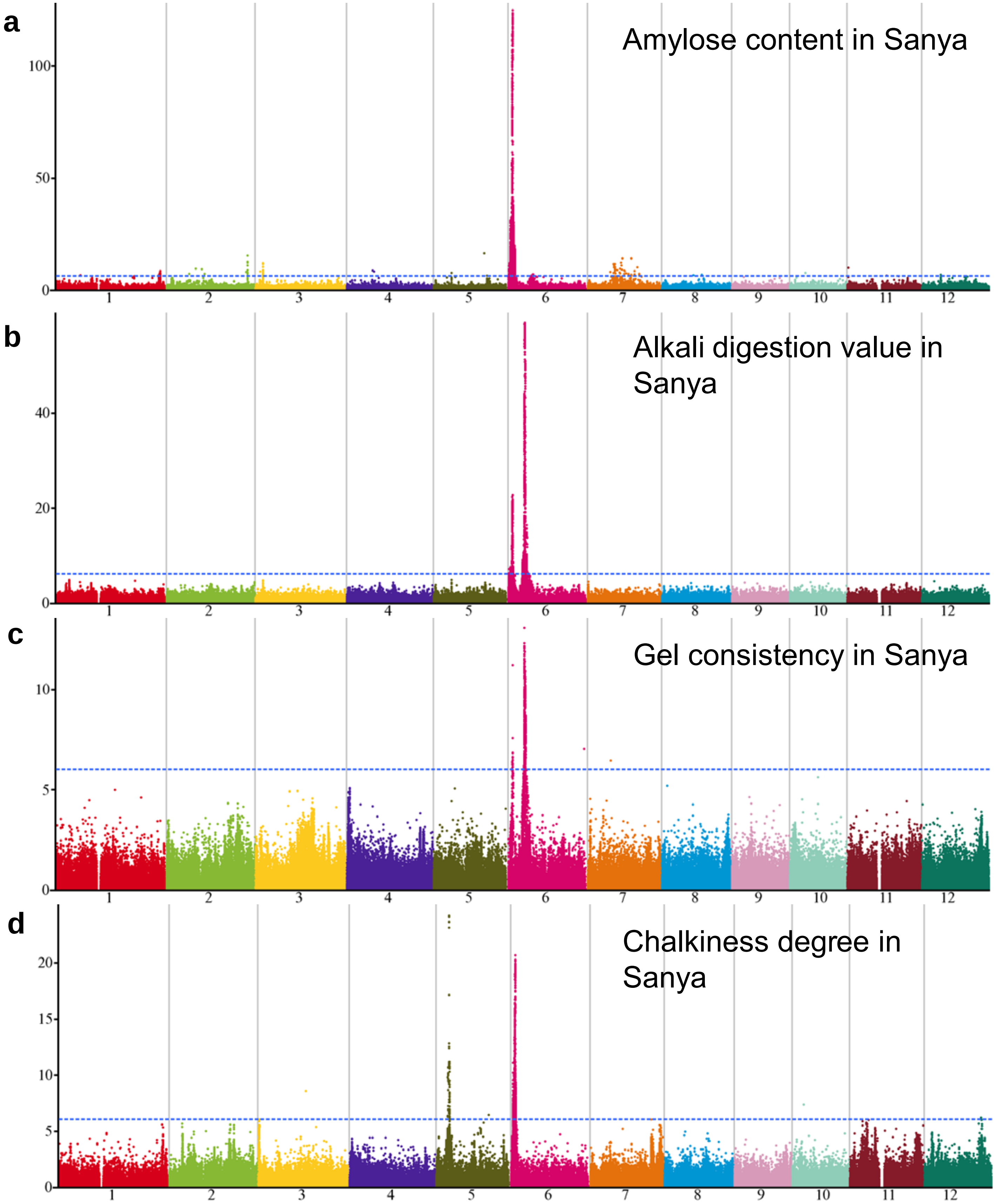

Supplementary Figure 12 GWAS of amylose content, alkali digestion, gel consistency and chalkiness degree in Sanya. Negative $\log_{10}$ *P*-values from linear mixed model (Y-axis) are plotted against SNP positions (X-axis) on each of 12 chromosomes. This Manhattan plot is the result from additive model.

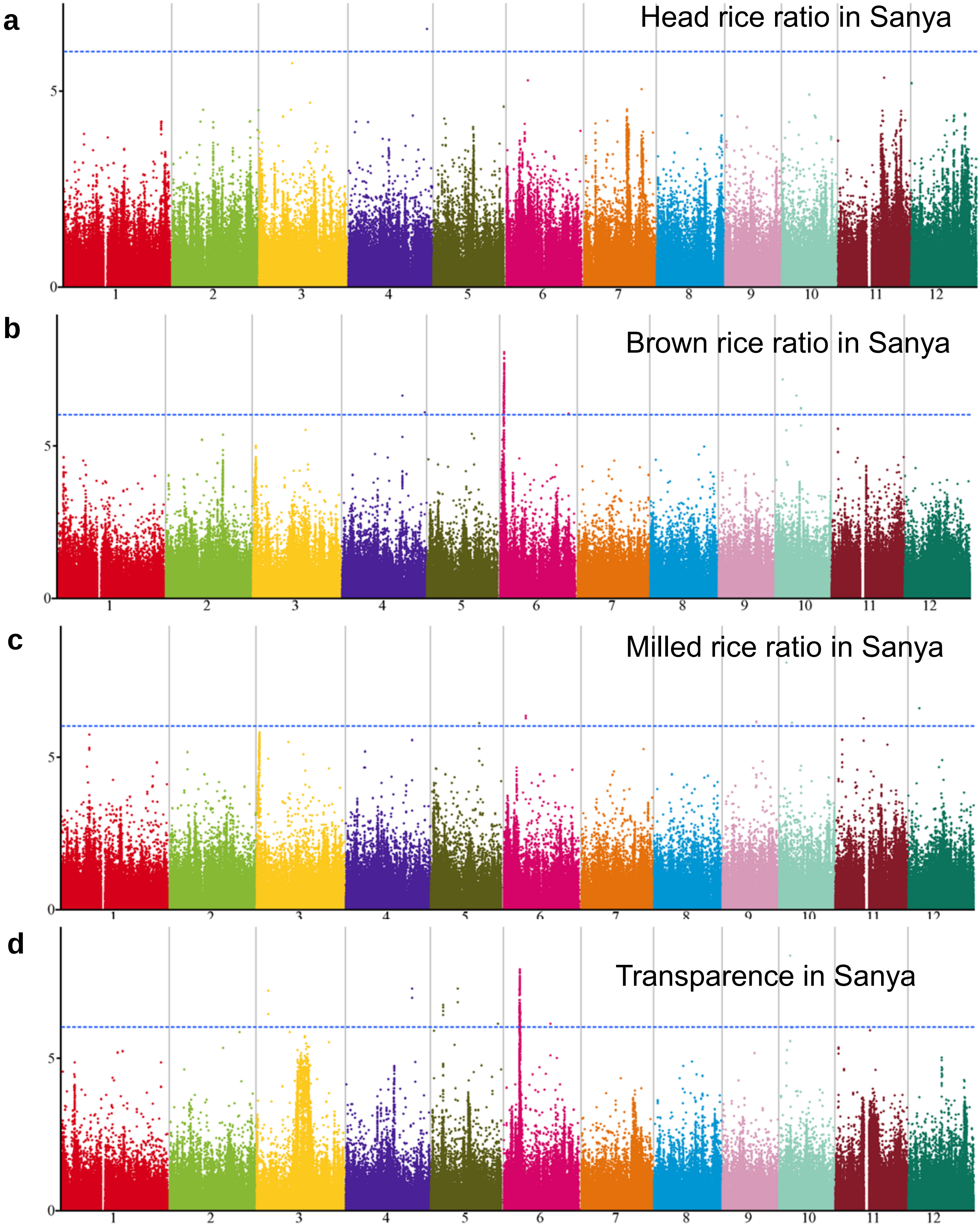

Supplementary Figure 13 GWAS of head rice ratio, brown rice ratio, milled rice ratio and transparence in Sanya. Negative $\log_{10}$ *P*-values from linear mixed model (Y-axis) are plotted against SNP positions (X-axis) on each of 12 chromosomes. This Manhattan plot is the result from additive model.

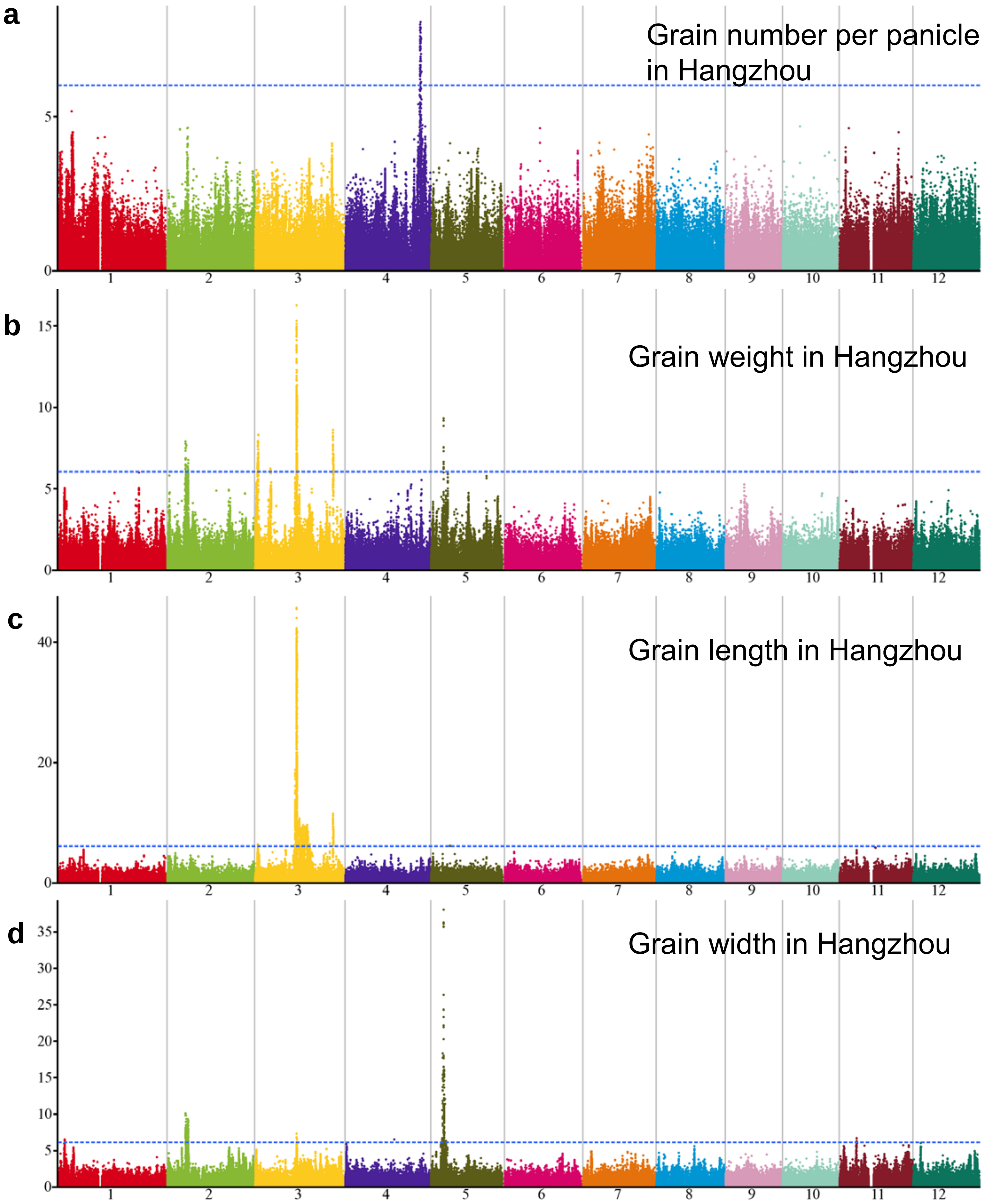

Supplementary Figure 14 GWAS of grain number, grain weight, grain length, grain width in Hangzhou. Negative $\log_{10}$ *P*-values from linear mixed model (Y-axis) are plotted against SNP positions (X-axis) on each of 12 chromosomes. This Manhattan plot is the result from additive model.

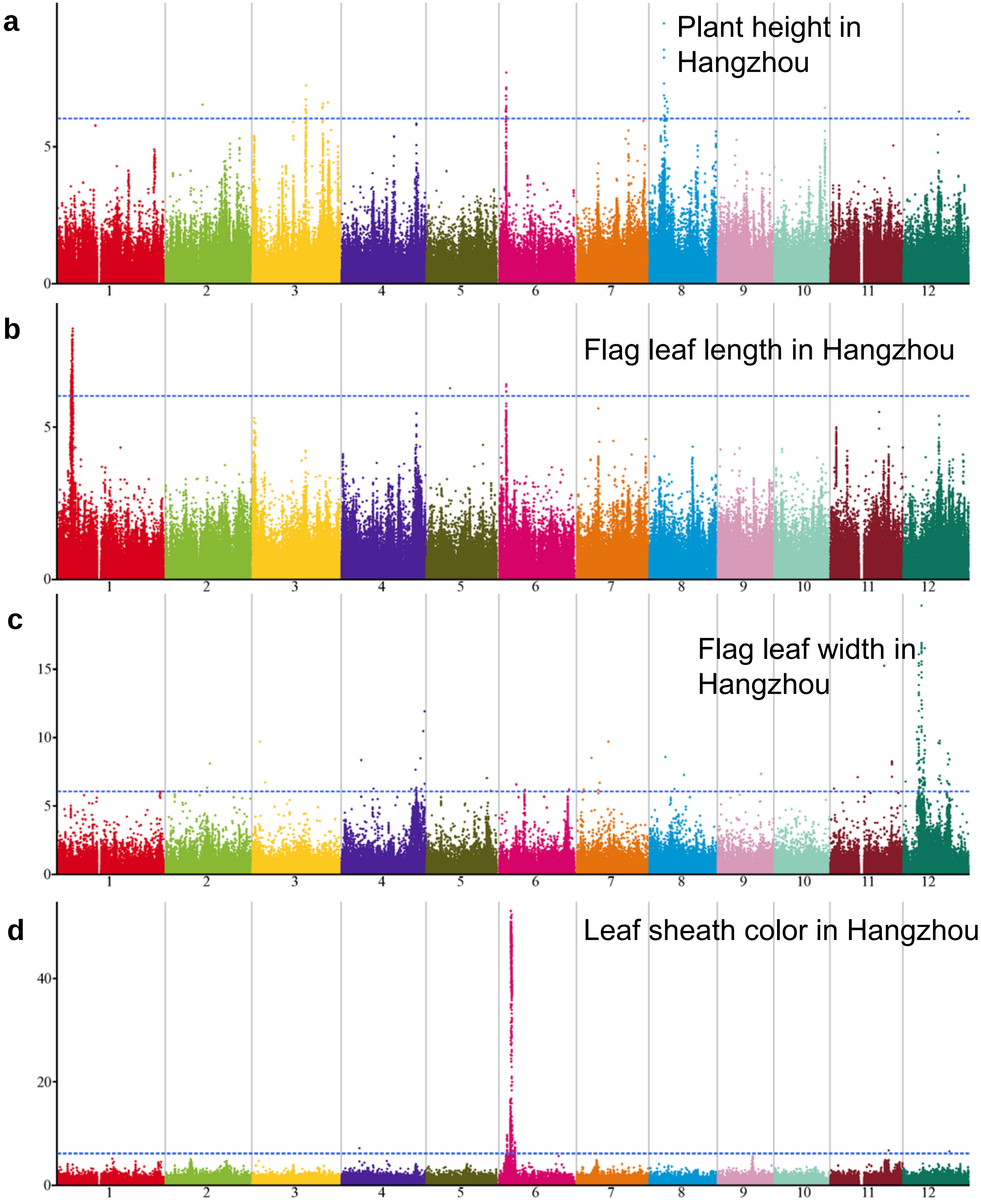

Supplementary Figure 15 GWAS of plant height, flag leaf length, flag leaf width and leaf sheath color in Hangzhou. Negative $\log_{10}$ *P*-values from linear mixed model (Y-axis) are plotted against SNP positions (X-axis) on each of 12 chromosomes. This Manhattan plot is the result from additive model.

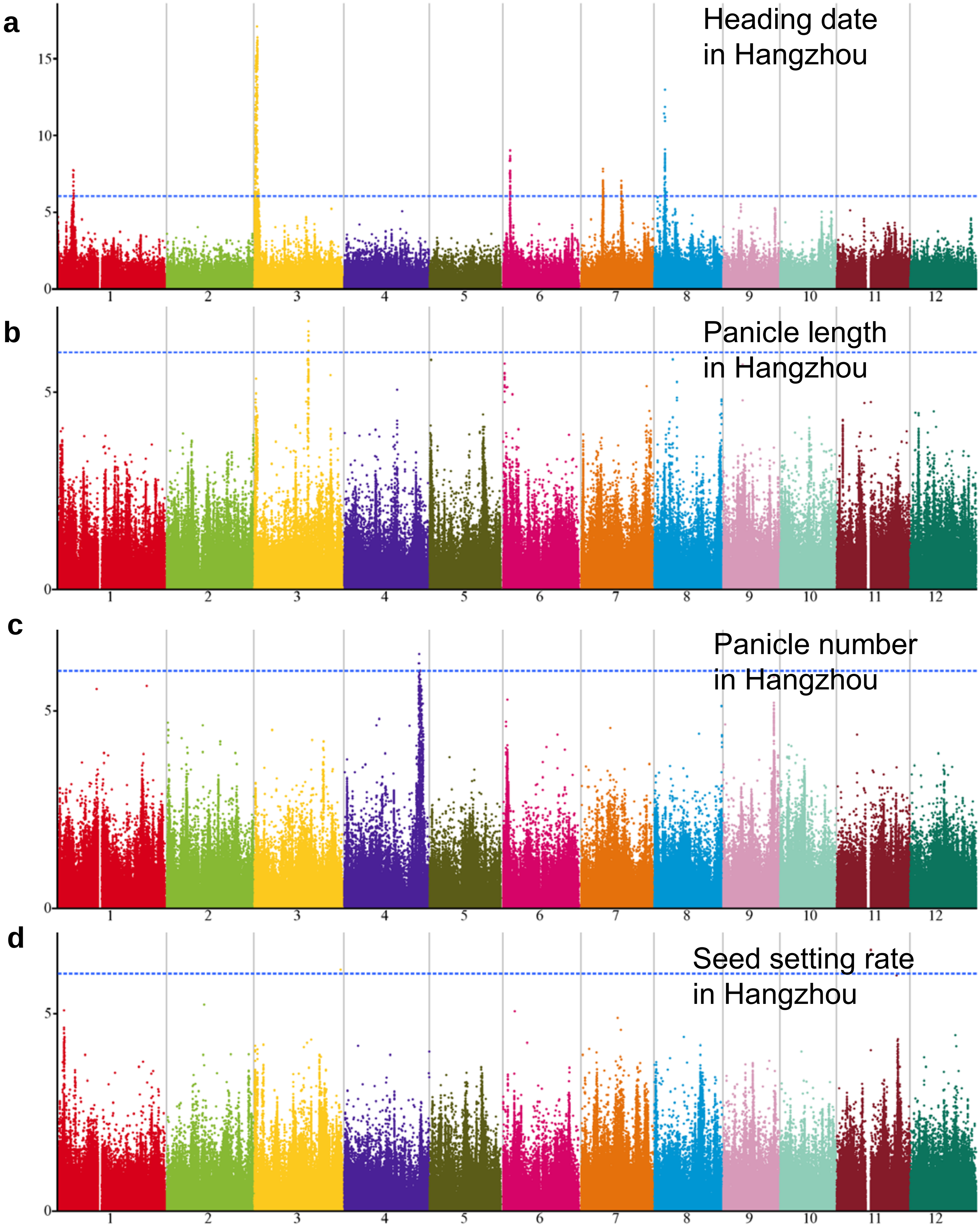

Supplementary Figure 16 GWAS of heading date, panicle length, panicle number and seed setting rate in Hangzhou. Negative $\log_{10}$ *P*-values from linear mixed model (Y-axis) are plotted against SNP positions (X-axis) on each of 12 chromosomes. This Manhattan plot is the result from additive model.

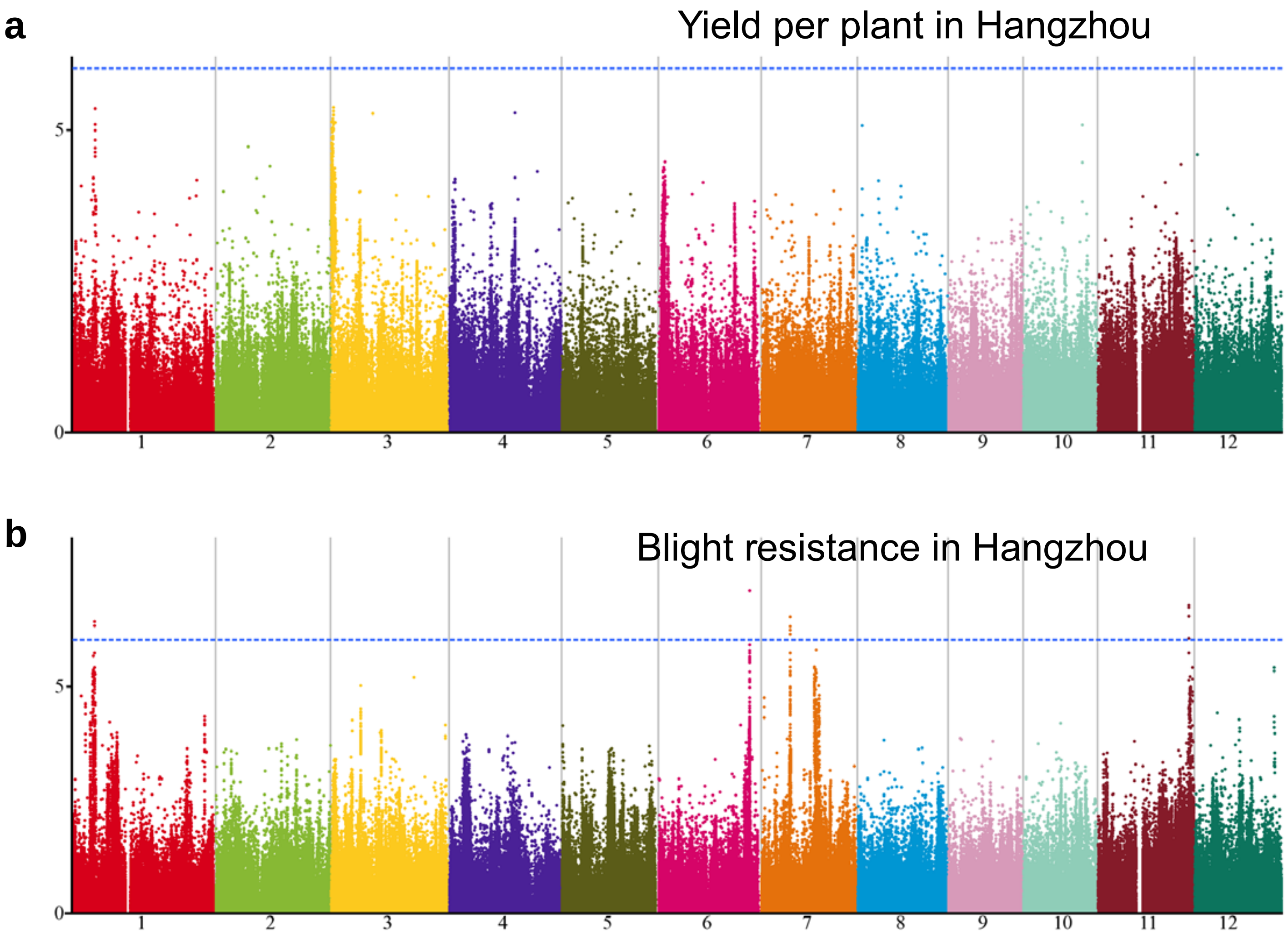

Supplementary Figure 17 GWAS of yield per plant and blight resistance in Hangzhou. Negative $\log_{10}$ *P*-values from linear mixed model (Y-axis) are plotted against SNP positions (X-axis) on each of 12 chromosomes. This Manhattan plot is the result from additive model.

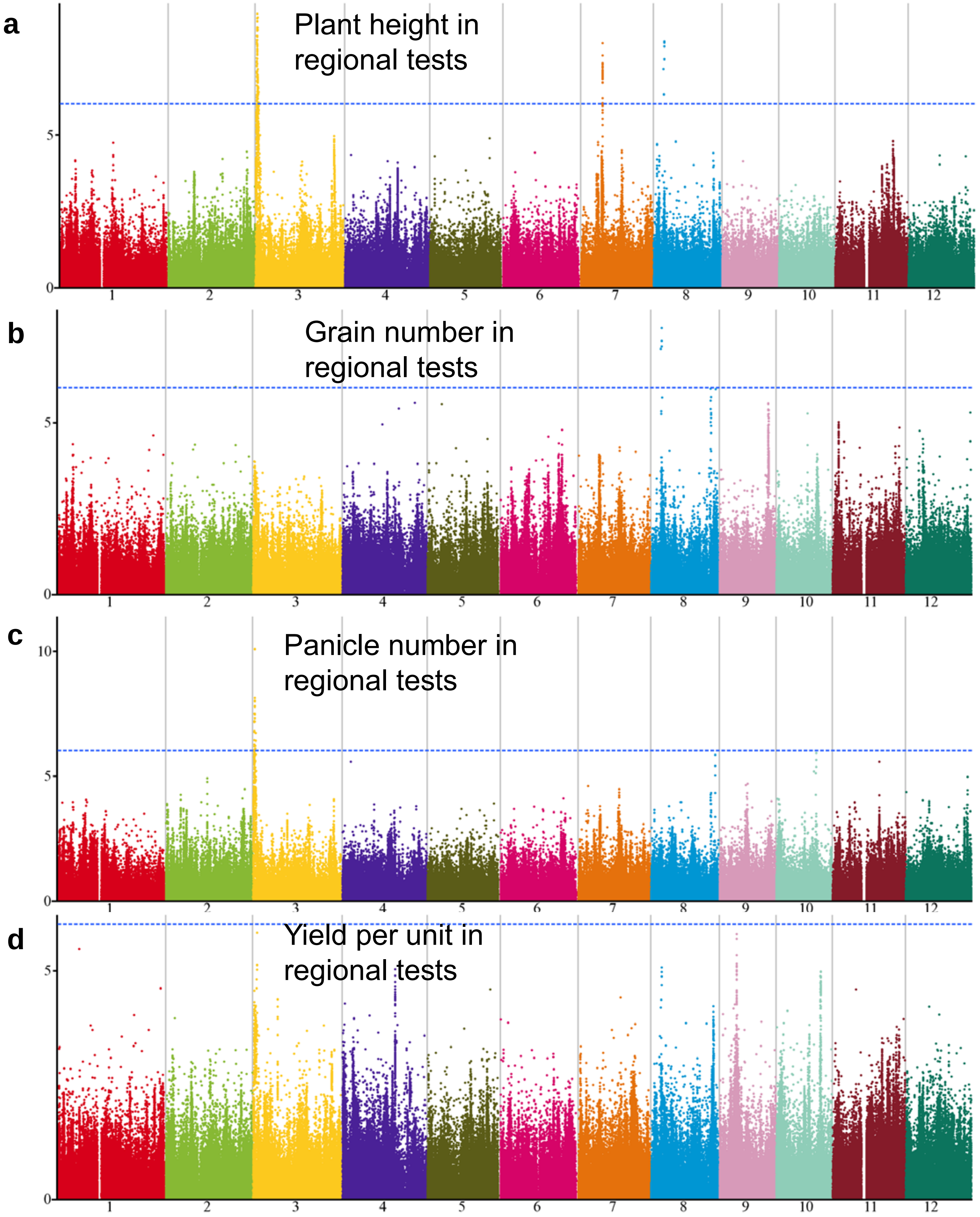

Supplementary Figure 18 GWAS of plant height, grain number, panicle number and yield per unit in regional trials. Negative $\log_{10}$ *P*-values from linear mixed model (Y-axis) are plotted against SNP positions (X-axis). This Manhattan plot is the result from additive model.

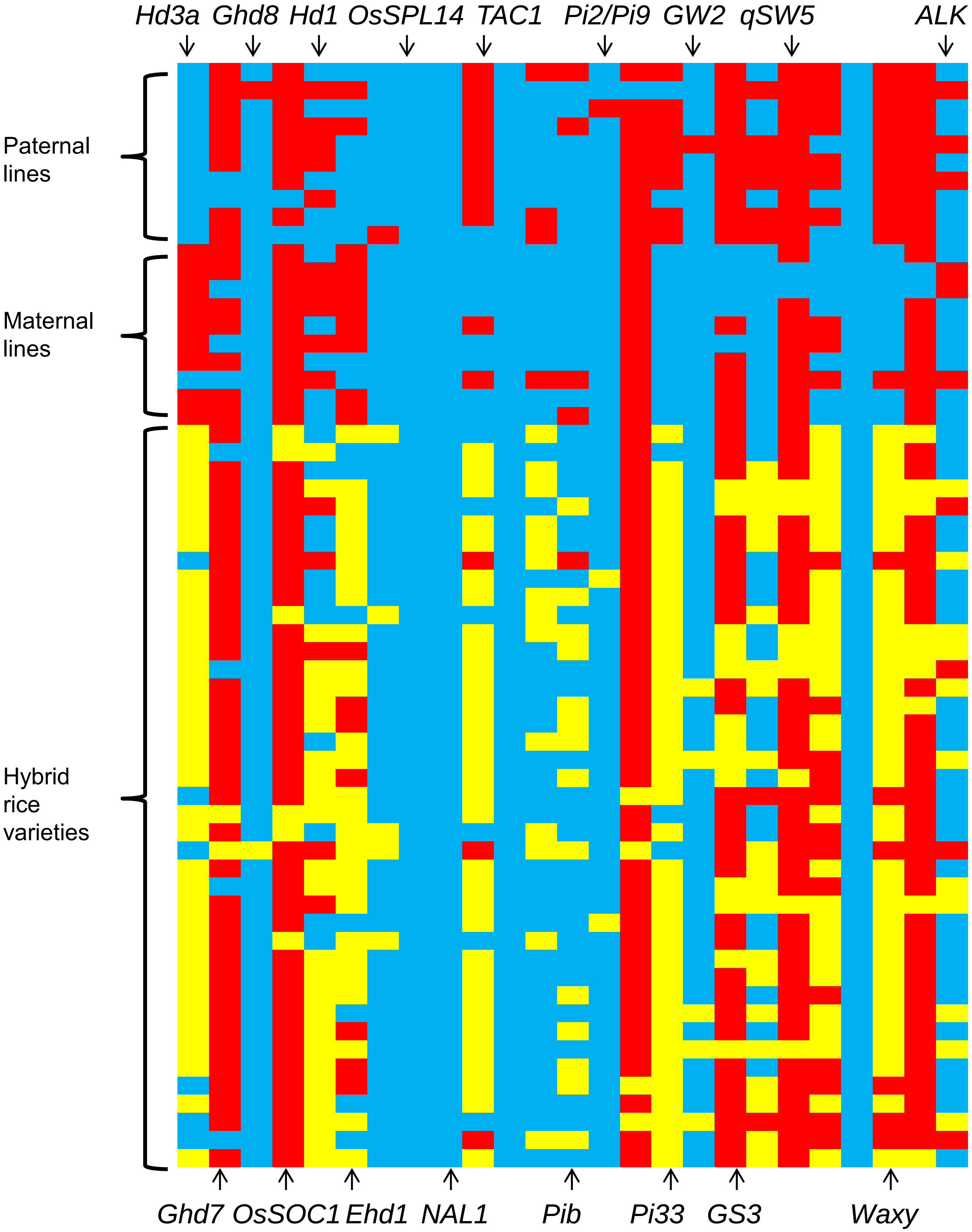

Supplementary Figure 19 Genotype visualization of hybrids and parental lines at GWAS loci. Heterozygous and both homozygous genotypes are indicated in yellow, red and blue, respectively. The lines are the top 10 maternal lines, the top 10 paternal lines and 41 hybrid combinations. The GWAS loci involve multiple important traits, with the candidate causal genes labeled.

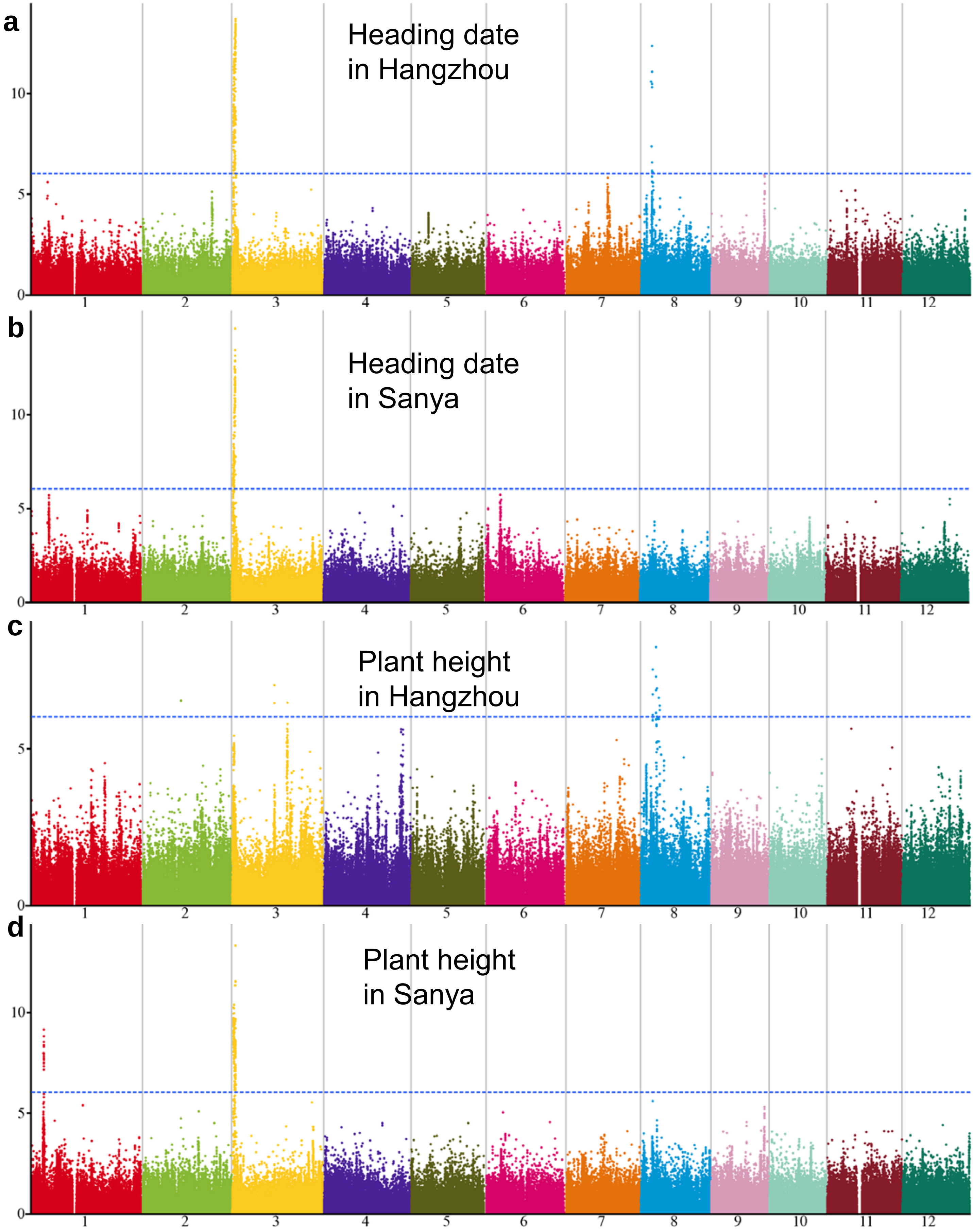

Supplementary Figure 20 GWAS of heading date and plant height using recoded SNPs. Both homozygous states were coded as one type and heterozygous genotype was coded as the other type.

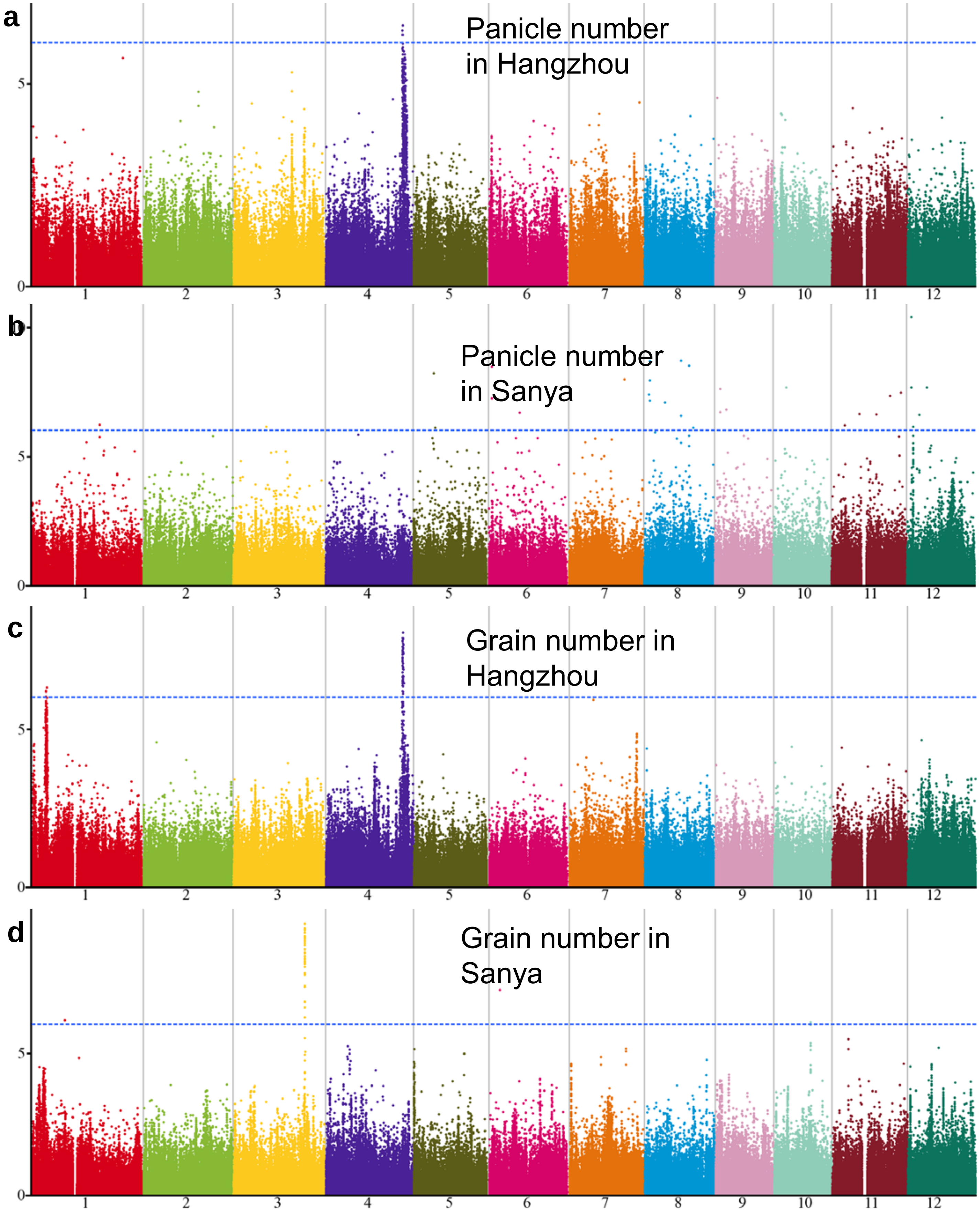

Supplementary Figure 21 GWAS of panicle number and grain number using recoded SNPs. Both homozygous states were coded as one type and heterozygous genotype was coded as the other type.

a

Chr.	Pos.	Geno0	Geno1	Geno2	-log(*P*)	Gene	Note
5	5340811	CC	TC	TT	17.8	***qSW5***	Geno0(CC), low chalky grain rate
6	1750840	AA	CA	CC	124.5	***Waxy***	Geno0(AA),low amylose content
6	6929482	TT	TA	AA	58.8	***ALK***	Geno0(TT), low gelatinization temperature

b

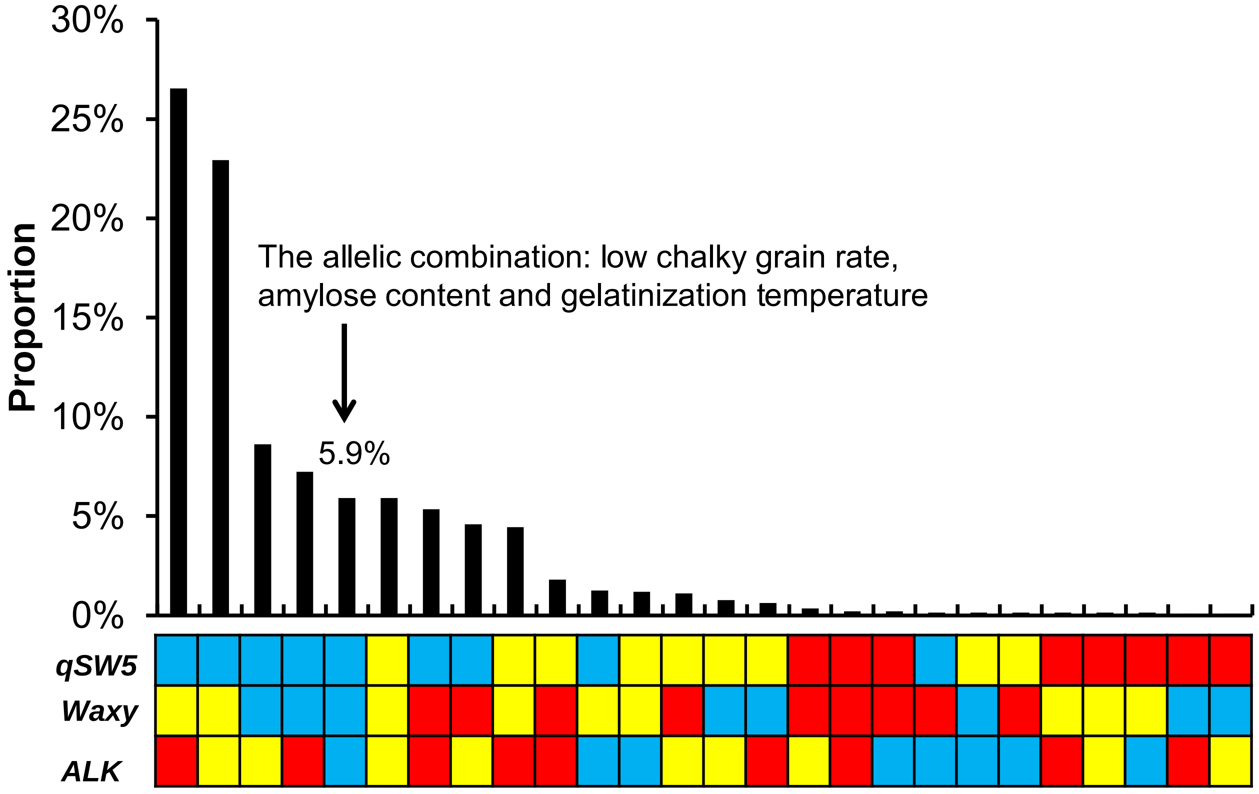

Supplementary Figure 22 The allelic combinations of three major loci for rice grain quality. (**a**) The information on the GWAS peaks of three major loci (*qSW5*, *Waxy* and *ALK*). (**b**) The proportion of 26 allelic combinations in the population of *indica* hybrids. The horizontal axis represents different allelic combinations. The first column, for example, indicates the proportion of the combination of Geno0 (in blue) in *qSW5*, Geno1 (in yellow) in *Waxy* and Geno2 (in red) in *ALK* in the *indica* hybrids.

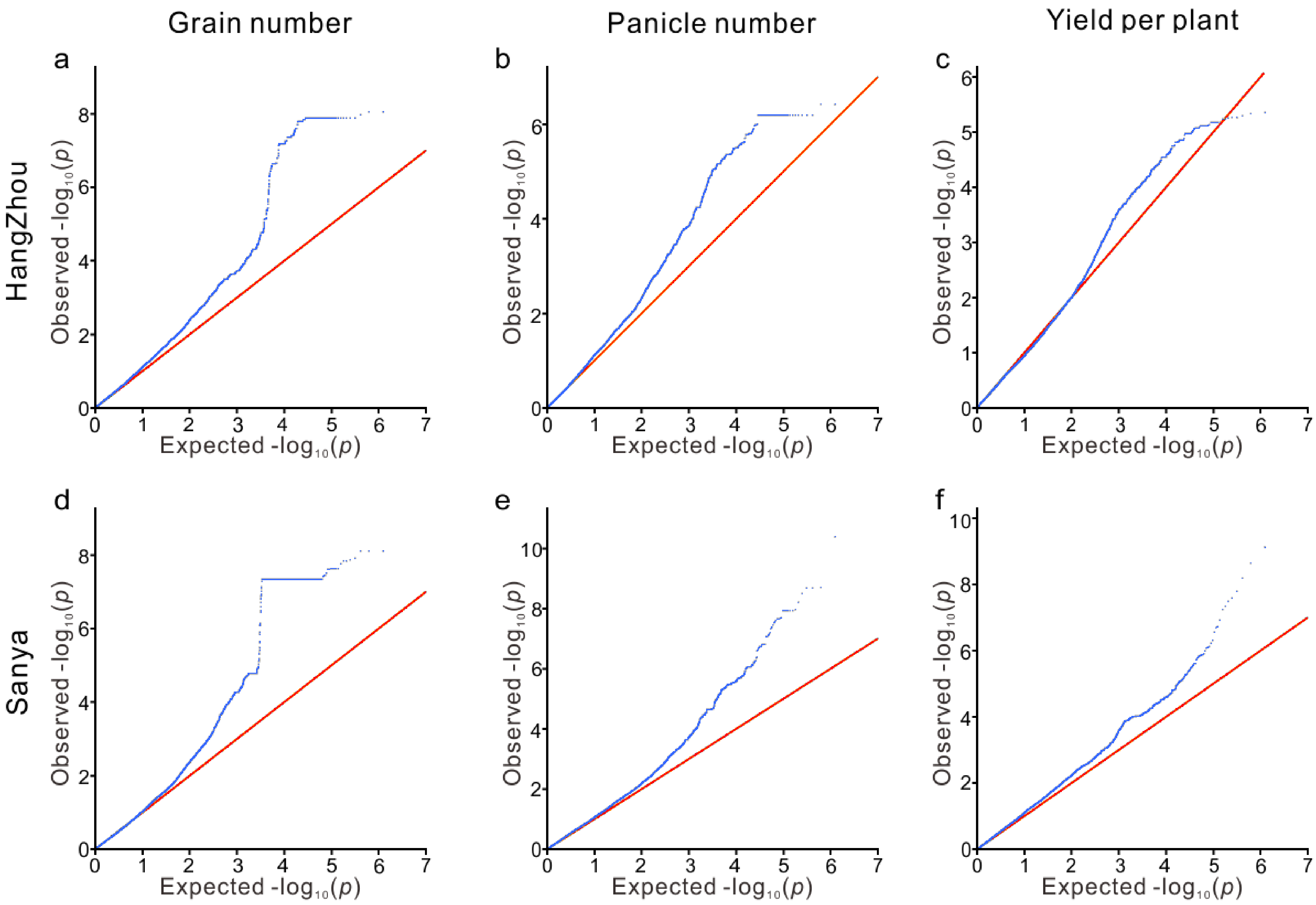

Supplementary Figure 23 Quantile-quantile plots of GWAS for three yield traits in Hangzhou and Sanya.

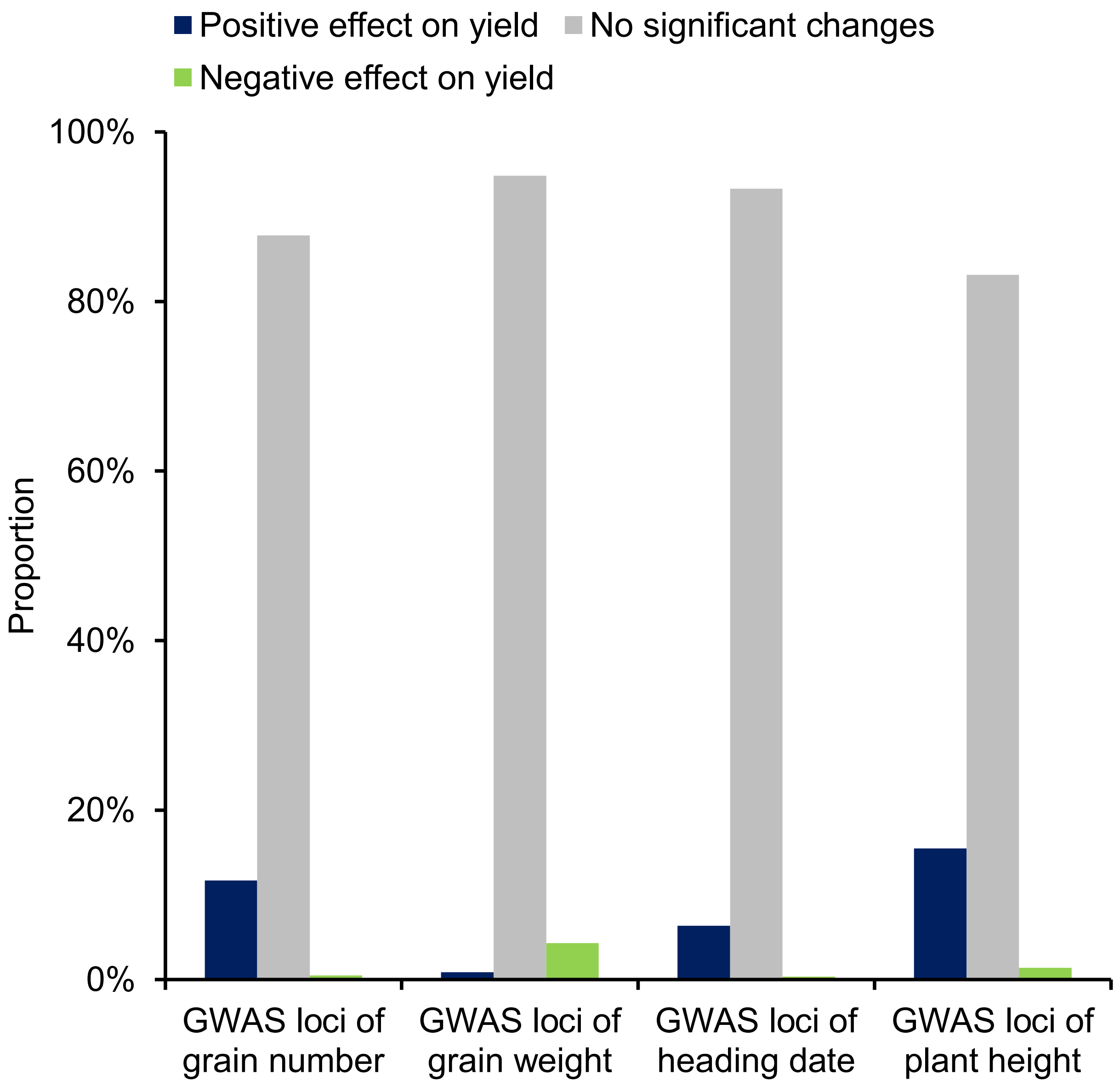

Supplementary Figure 24 Effects of GWAS loci of yield-related traits on yield per plant in Sanya. For each yield-related traits, we identified all associated loci with P<0.01 and the phenotypic effects of different alleles. For the alleles at the associated loci, their genetic effects (significant or not; positive or negative) on yield per plant were further analyzed.

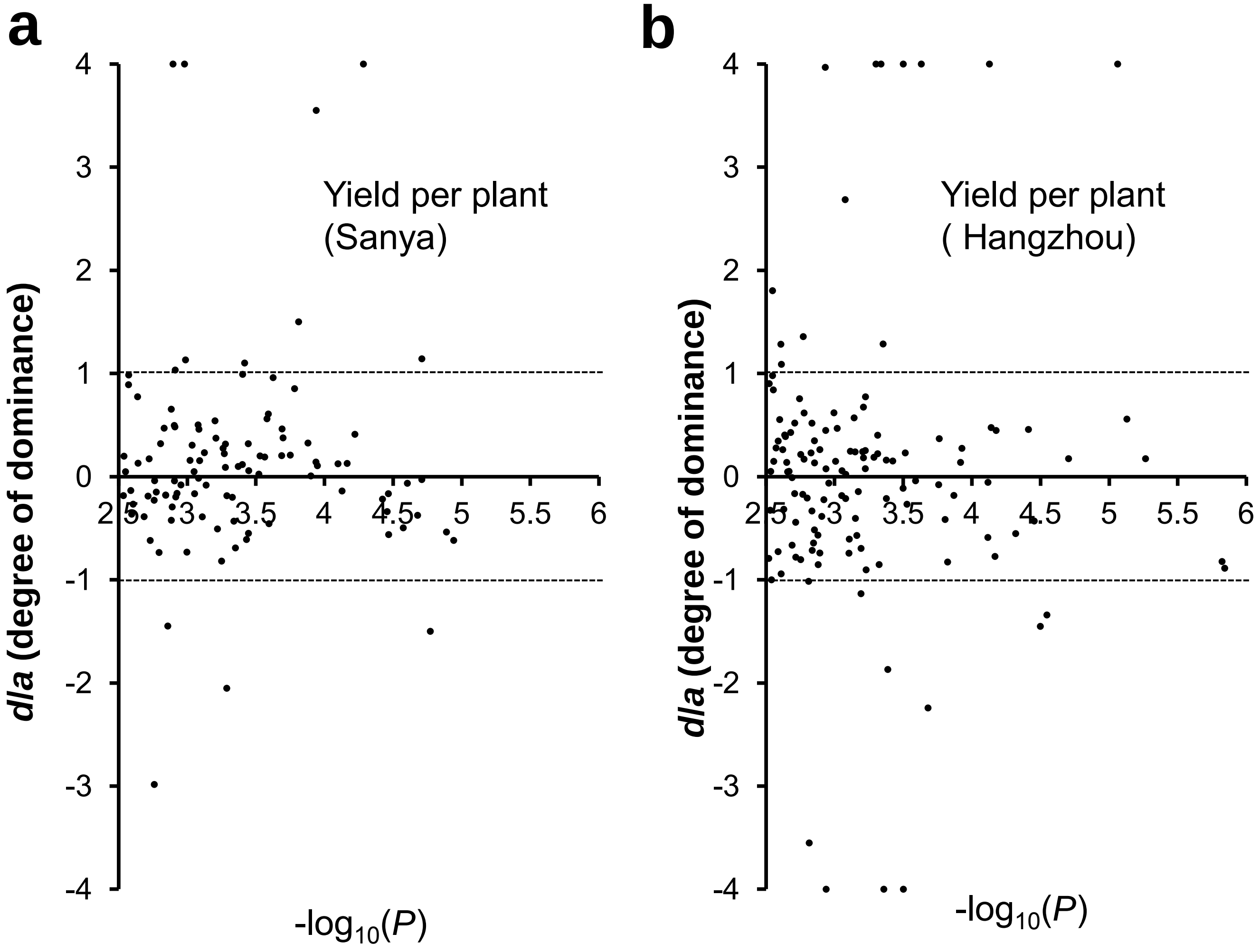

Supplementary Figure 25 The degree of dominance of the associated loci for yield per plant in Sanya and Hangzhou. For each associated locus with $-\log_{10}(P)>2.5$ (calculated from EMMAX), the genotypes at the peak SNP was used to calculated the degree of dominance (*d*/*a*, where *d* is the dominant effect and *a* is the additive effect). The dividing lines (1 and -1) between dominance and over-dominance are indicated by horizontal dash-dot lines. In this plotting, the loci with excessive dominance degree, namely *d*/*a* > 4 and *d*/*a* < -4, are set to be the score of 4 and -4,respecitvely.

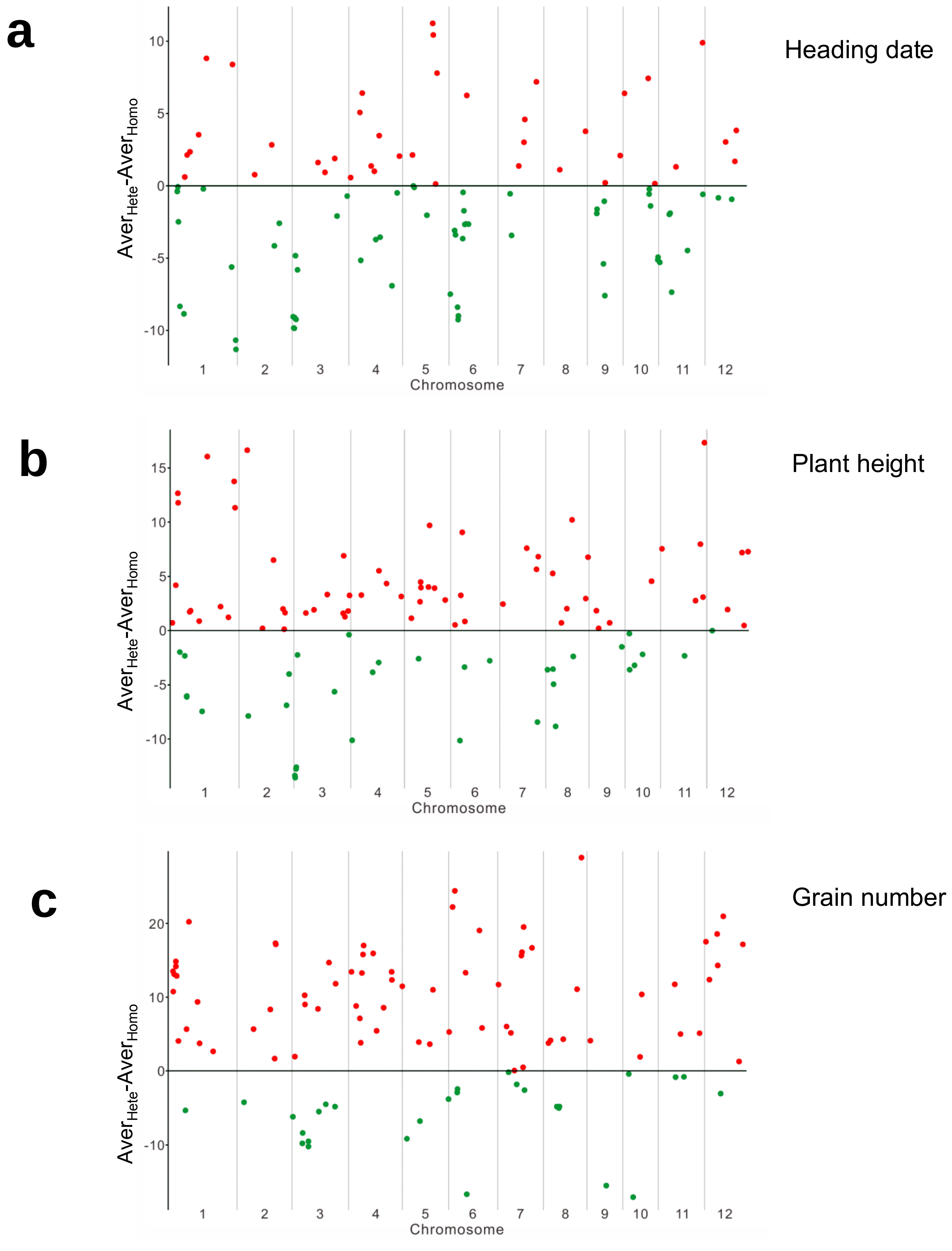

Supplementary Figure 26 The effects of heterozygous and homozygous genotypes of the associated loci for heading date, plant height and grain number in Sanya. For each of the top 100 associated loci (ranked in GWAS *P*-value from mixed linear model), the average effect of the heterozygous genotypes at the peak SNP ($Aver_{Hete}$) and that of homozygous genotypes ($Aver_{Homo}$) were calculated in the *indica* hybrids. The SNPs with positive values and negative values are colored in red and green, respectively.

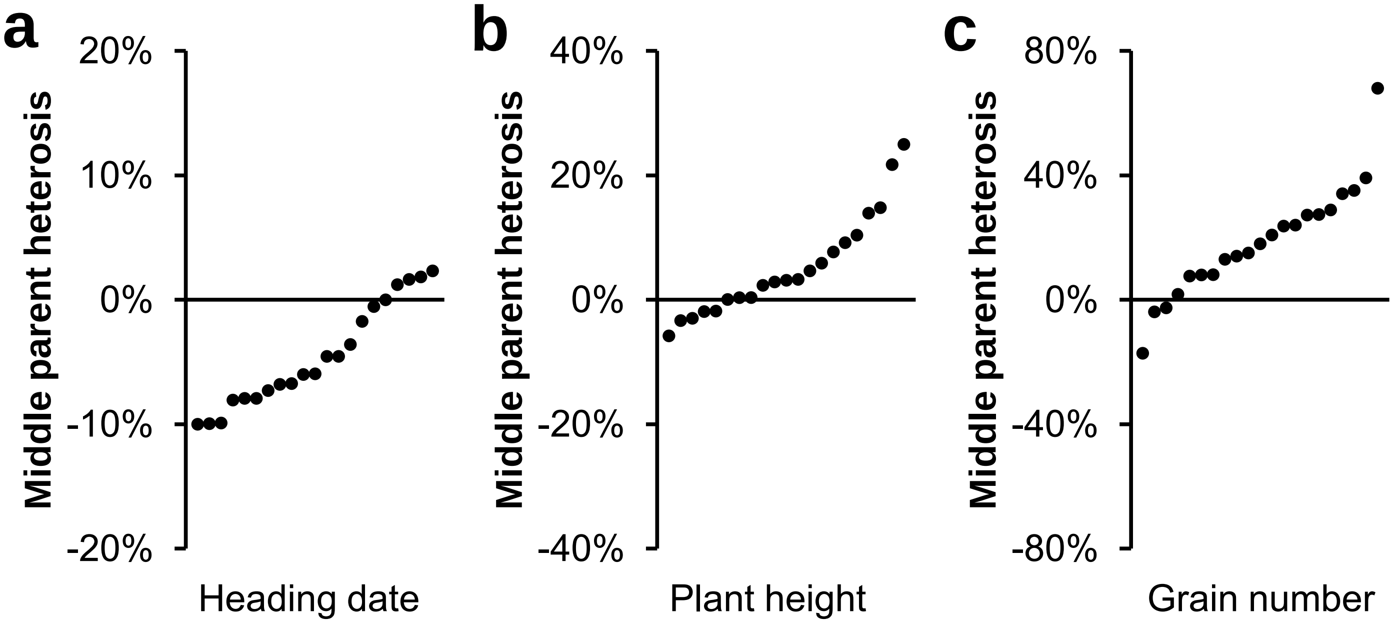

Supplementary Figure 27 Middle parent heterosis of heading date, plant height and grain number in parents-hybrid trios. For the parents-child trios, the phenotype of both parents and the hybrids were carefully evaluated in Sanya. Each parents-hybrid trio is represented by a black dot, which is ordered by its level of middle parent heterosis in the plotting. The index of middle parent heterosis was calculated as $(F_1-P_1/2-P_2/2)/(P_1/2+P_2/2)$ for whole-genome hetosis performance, where F_1, P_1 and P_2 are their corresponding phenotypic measurements of the hybrid and both parents.

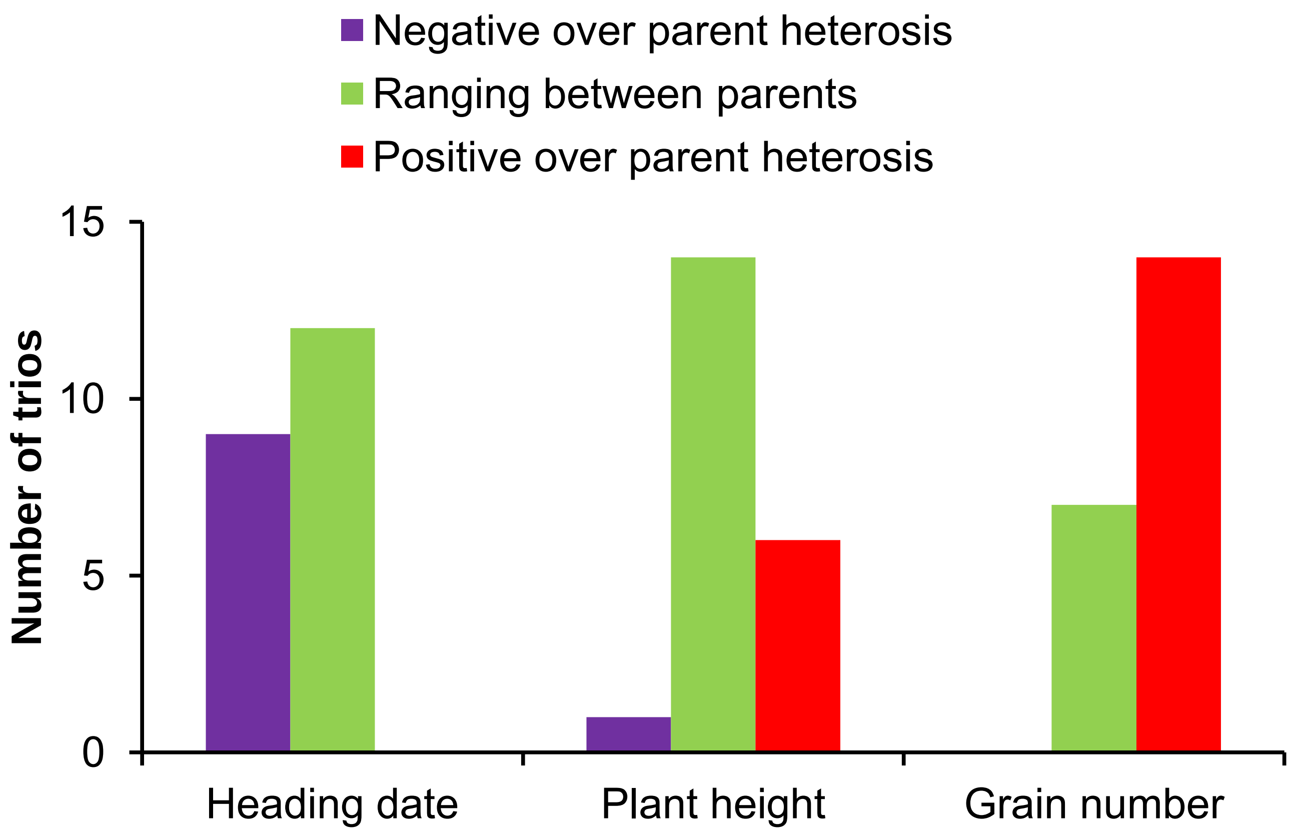

Supplementary Figure 28 Over parent heterosis of heading date, plant height and grain number in parents-hybrid trios. For the parents-child trios, the phenotype of both parents and the hybrids (F_1, P_1 and P_2) were carefully evaluated in Sanya. Positive over parent heterosis: F_1 is better than both parents. Negative over parent heterosis: F_1 is worse than both parents.

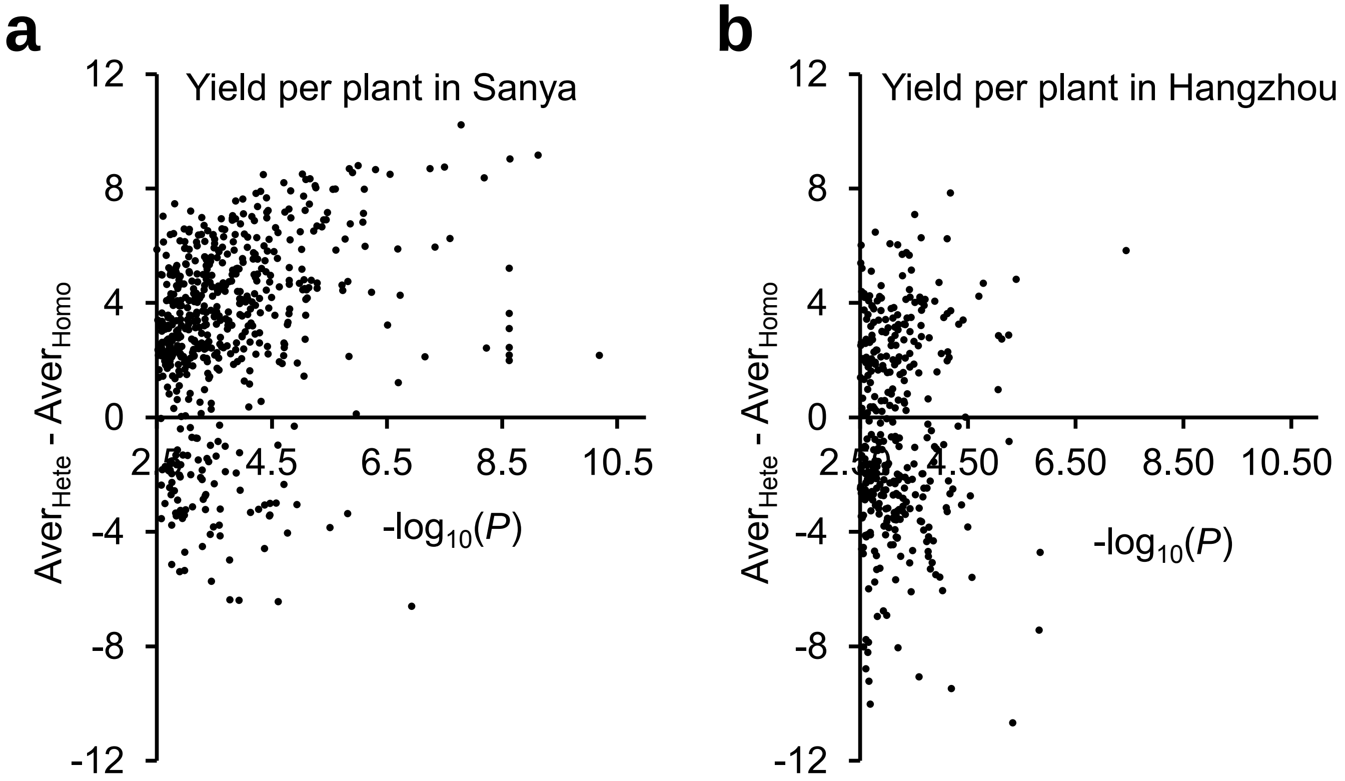

Supplementary Figure 29 The effects of heterozygous and homozygous genotypes of the associated loci for yield per plant in Sanya and Hangzhou. For each associated locus with $-\log_{10}(P)>2.5$ (calculated from mixed linear model), the average effect of the heterozygous genotypes at the peak SNP ($Aver_{Hete}$) and that of homozygous genotypes ($Aver_{Homo}$) were calculated in the *indica* hybrids.

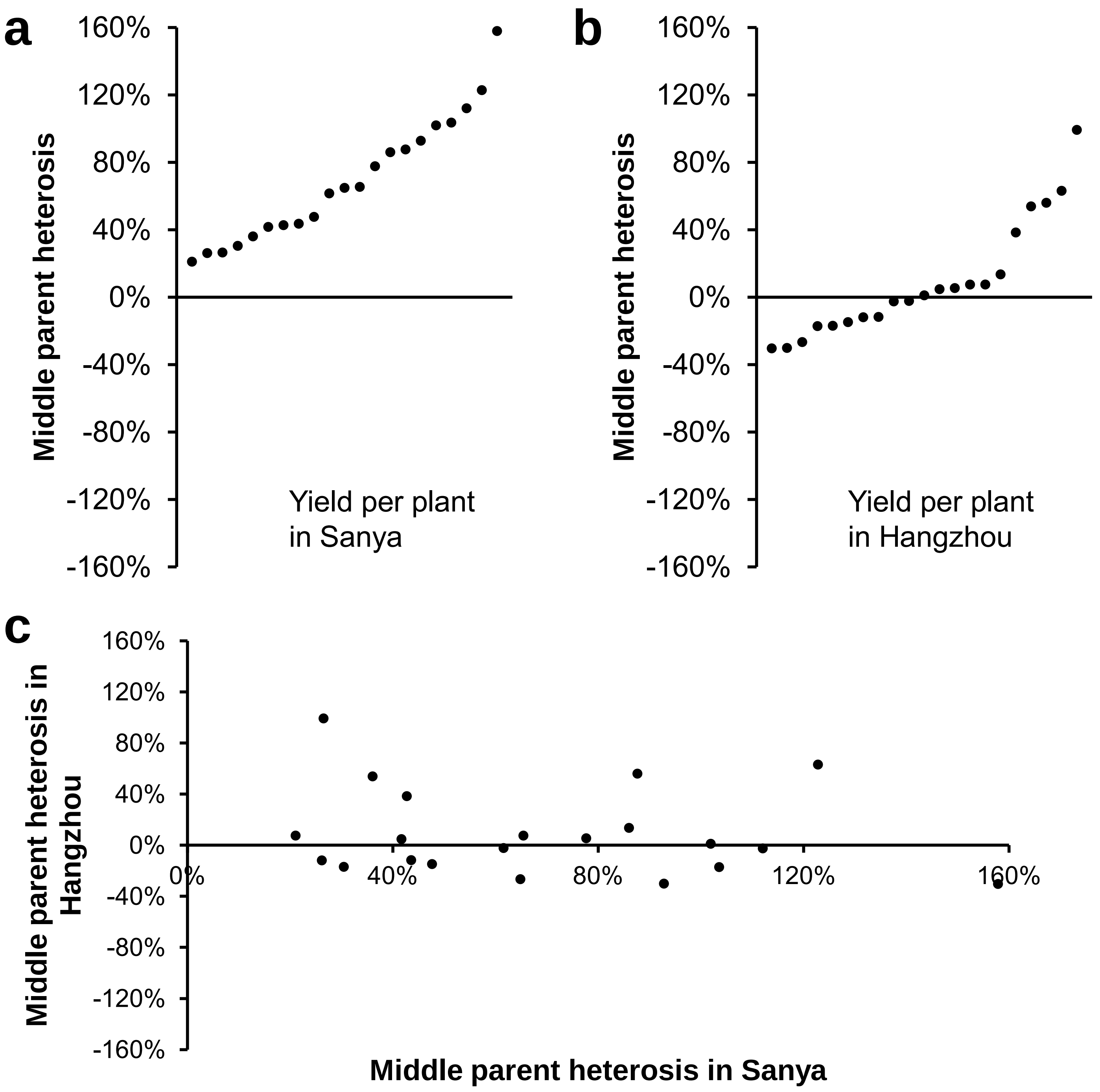

Supplementary Figure 30 Middle parent heterosis of yield per plant in parents-hybrid trios. For the parents-child trios, the phenotype of both parents and the hybrids were carefully evaluated in Sanya and Hangzhou. Each parents-hybrid trio is represented by a black dot, which is ordered by its level of middle parent heterosis in the plotting. The index of middle parent heterosis was calculated as $(F_1-P_1/2-P_2/2)/(P_1/2+P_2/2)$ for whole-genome hetosis performance, where F_1, P_1 and P_2 are their corresponding phenotypic measurements of the hybrid and both parents.

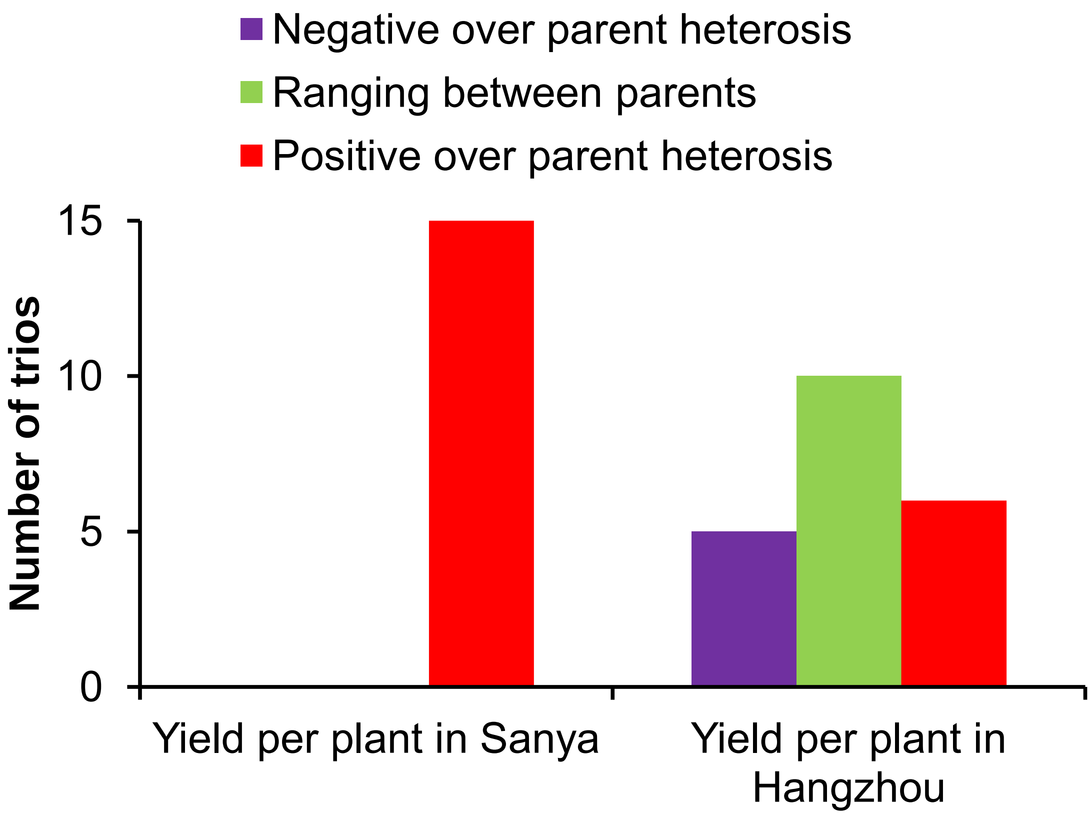

Supplementary Figure 31 Over parent heterosis of yield per plant in parents-hybrid trios. For the parents-child trios, the yield per plant of both parents and the hybrids (F_1, P_1 and P_2) were carefully evaluated in Sanya and Hangzhou. Positive over parent heterosis: F_1 is better than both parents. Negative over parent heterosis: F_1 is worse than both parents.

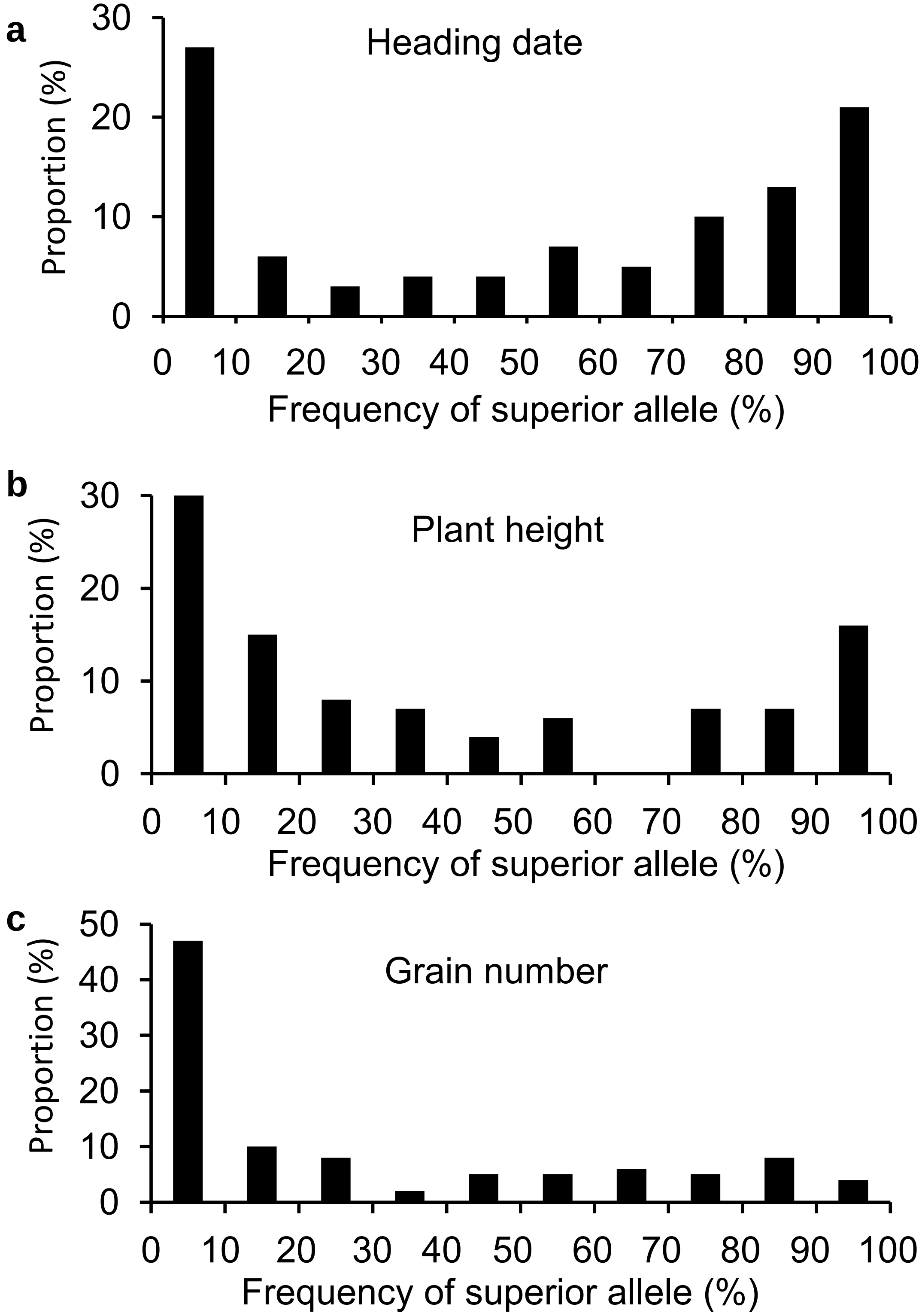

Supplementary Figure 32 Distribution of the frequencies of superior alleles. Frequency distribution of superior alleles of the associated loci for heading date (**a**), plant height (**b**) and grain number (**c**) in Sanya. For each associated locus, the frequency of superior gene alleles was calculated at the population scale (1439 *indica* hybrids). Based on the frequency, the superior alleles from the associated loci were further categorized into ten groups, from very low (0-10%) to very high (90%-100%). The proportion of each group was displayed.

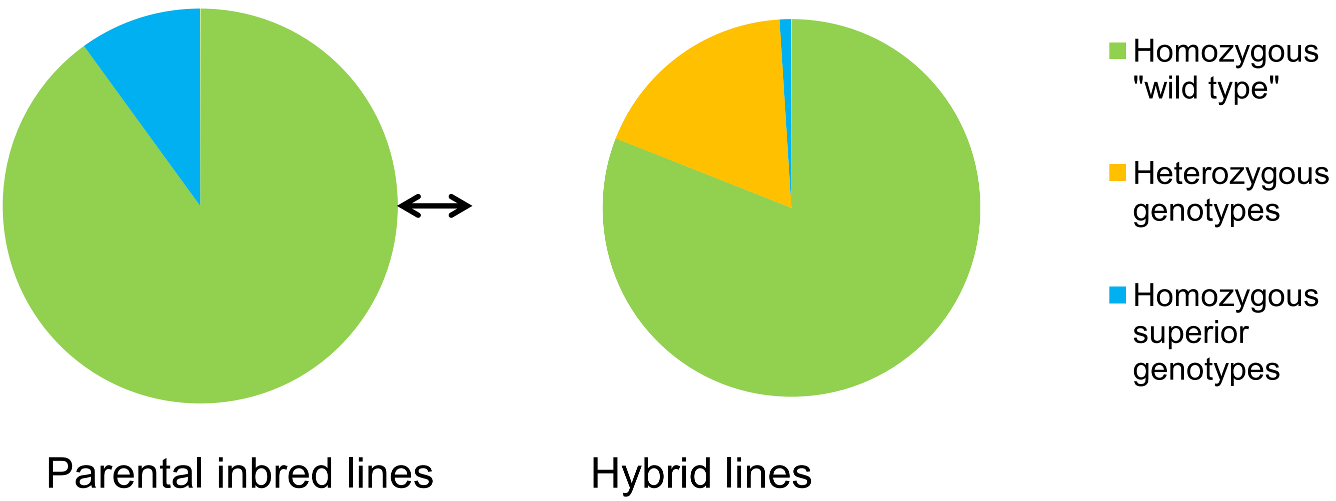

Supplementary Figure 33 Illustration of genotypes with rare superior alleles in inbred and hybrid lines. For a superior allele with 10% frequency in the population, the proportions of three genotypes (heterozygous genotype and both homozygous genotypes) were calculated in the inbred lines and hybrid lines according to the Hardy-Weinberg equilibrium.

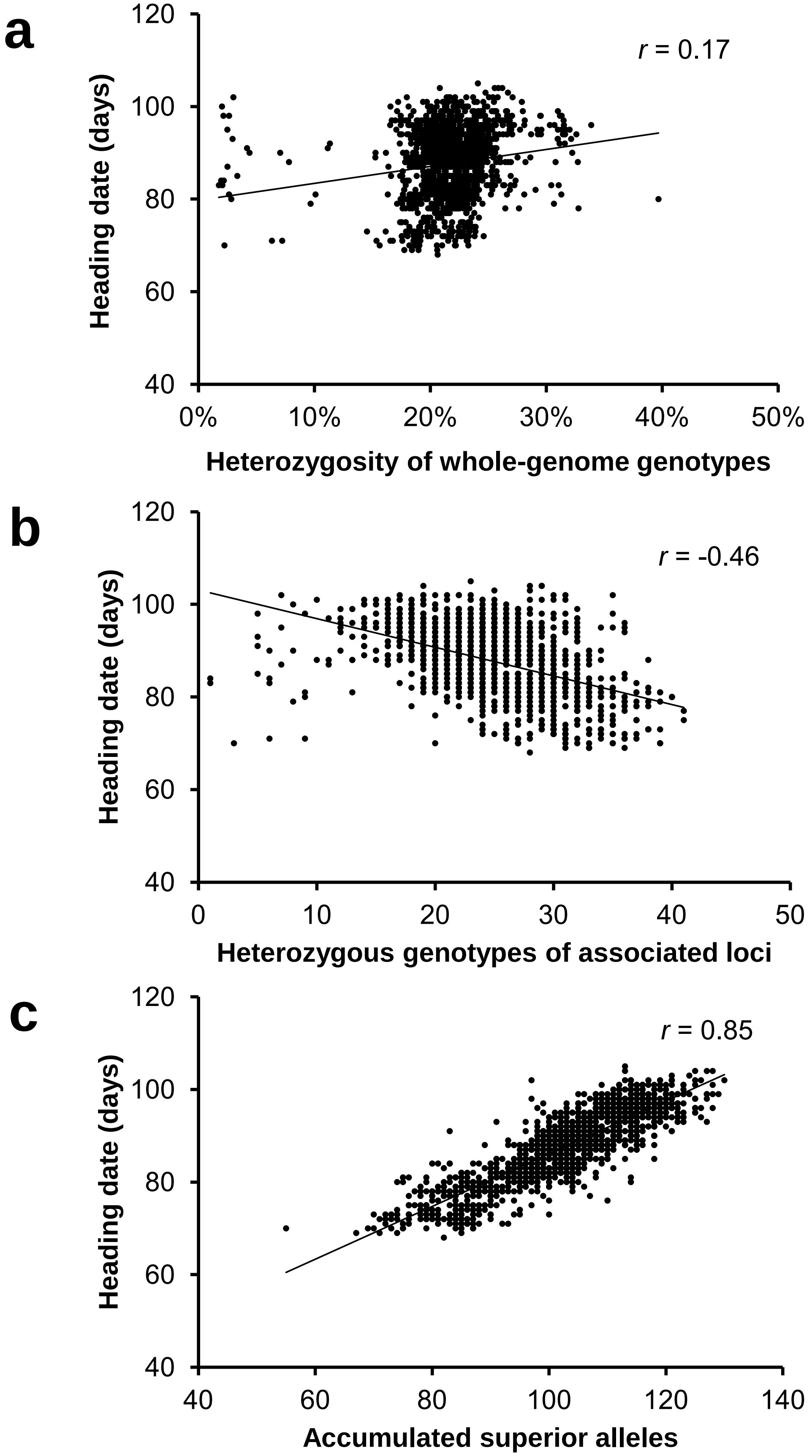

Supplementary Figure 34 The relationships between superior alleles and phenotypic variation of heading date in Sanya. We used the peak SNPs at the top 100 associated loci (ranked in GWAS *P*-value for heading date) to calculate heterozygous genotypes and accumulated superior alleles of each *indica* hybrid.

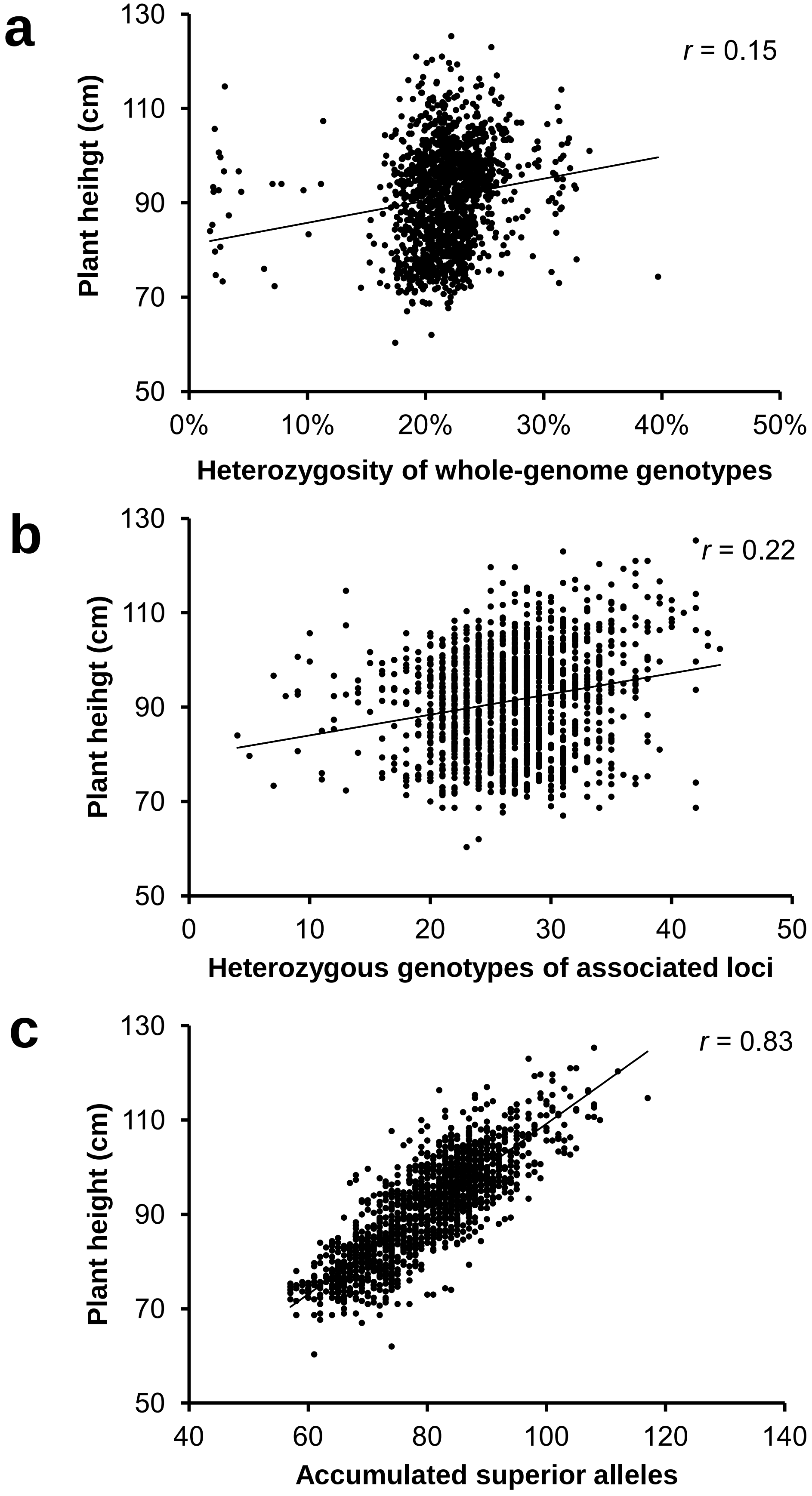

Supplementary Figure 35 The relationships between superior alleles and phenotypic variation of plant height in Sanya. We used the peak SNPs at the top 100 associated loci (ranked in GWAS *P*-value for plant height) to calculate heterozygous genotypes and accumulated superior alleles of each *indica* hybrid.

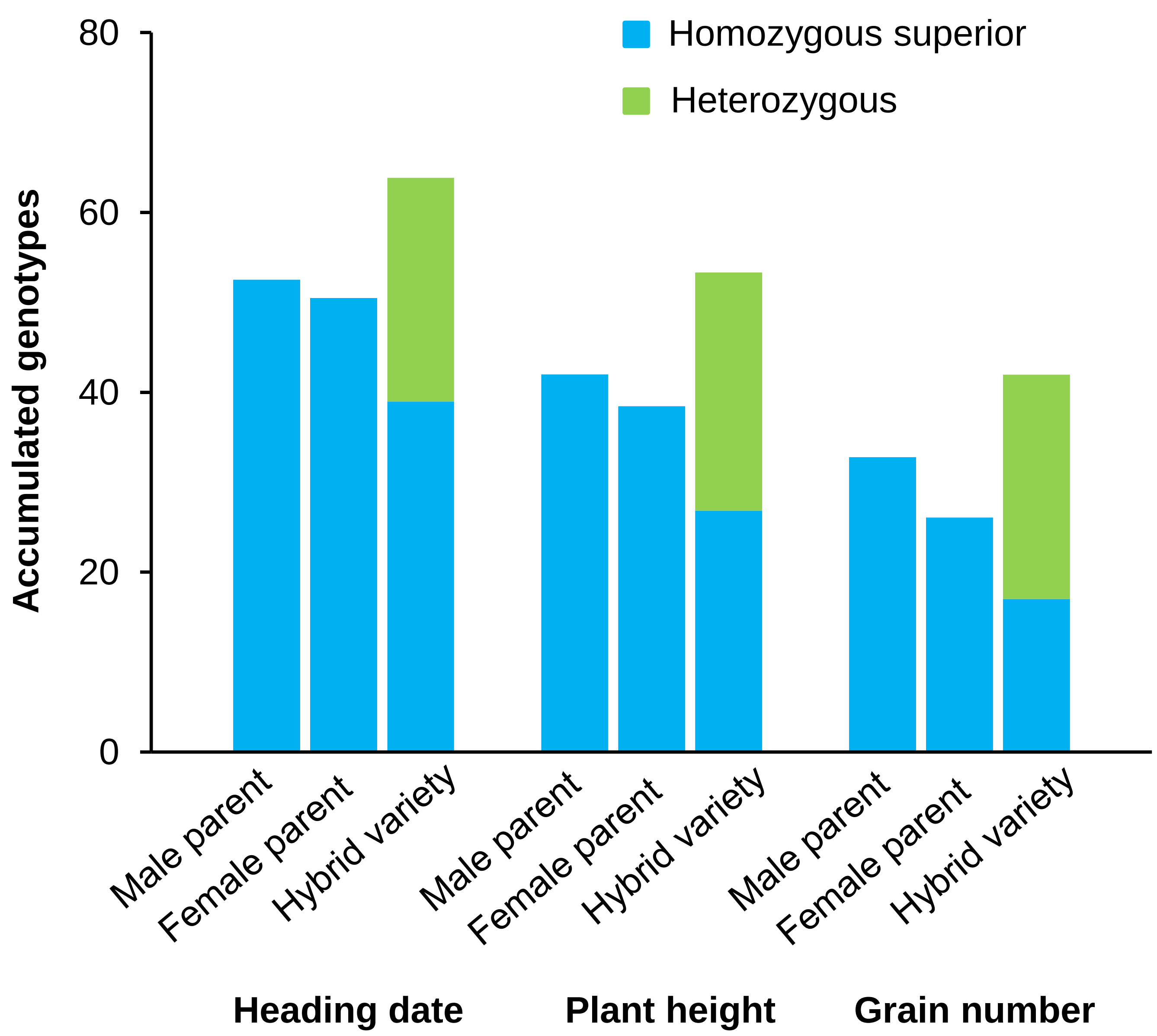

Supplementary Figure 36 The averaged number of accumulated genotypes. We used the peak SNPs at the top 100 associated loci (ranked in GWAS *P*-value) for heading date, plant height and grain number per panicle, respectively. Accumulated homozygous superior genotypes and accumulated heterozygous genotypes were counted in the *indica* hybrid lines and their inbred parental lines.

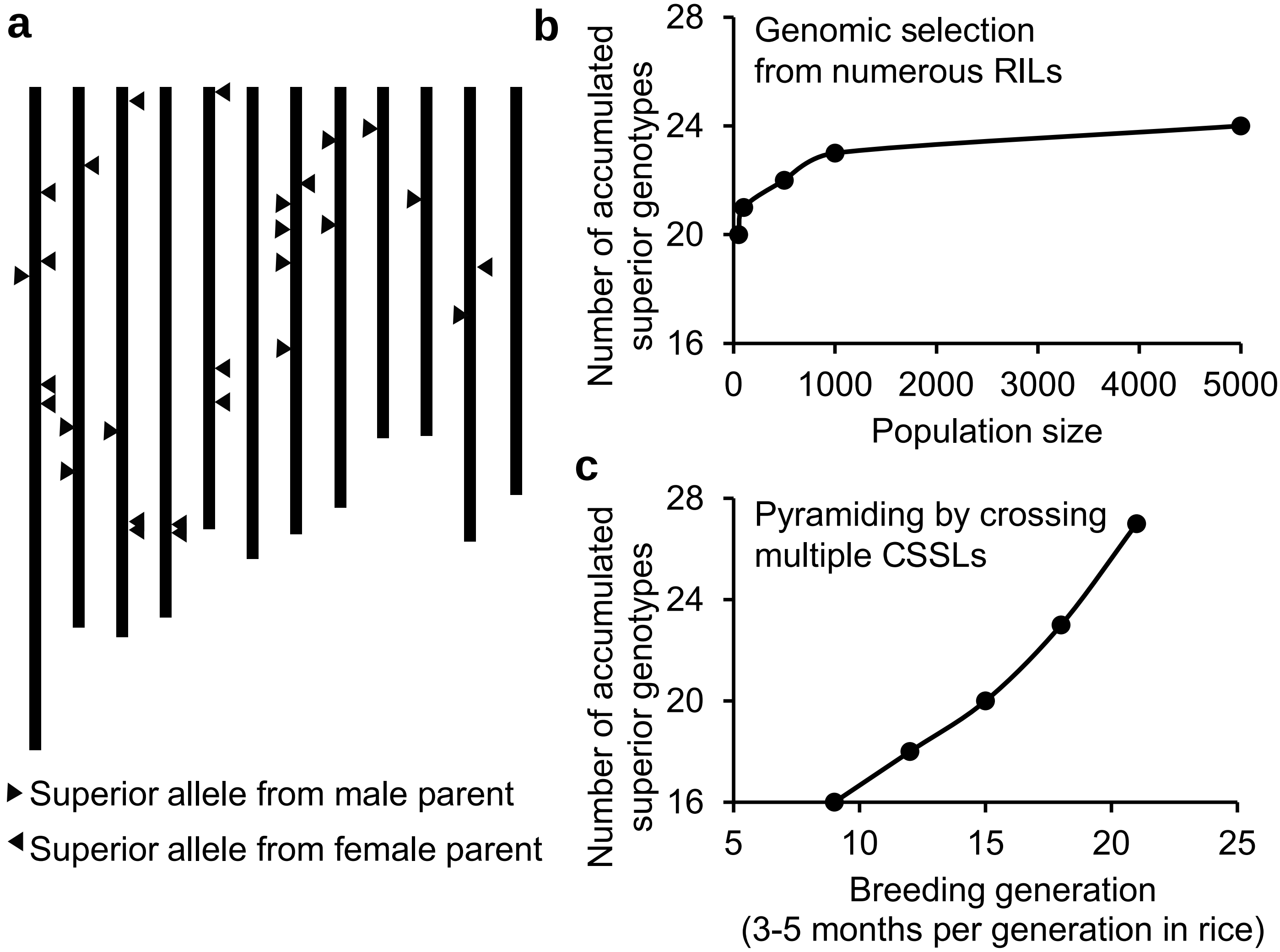

Supplementary Figure 37 Computational simulation of superior allele pyramiding. (**a**) An elite hybrid variety Yliangyou1hao is used as a template. The hybrid line is generated from a cross between male parent 9311 and female parent Y58S. Of the top 100 associated loci (ranked in GWAS *P*-value) for grain number per panicle, there are 28 segregating loci (thus heterozygous in F_1), of which the genomic locations were indicated. Among the 28 segregating loci, superior alleles of 13 loci are from 9311 genotype, and those of 15 loci are from Y58S genotype. (**b**) *In silico* genotype data of recombinant inbred line (RIL) population between both parents was generated using the sim.map and sim.cross functions in the R/Bioconductor package. The accumulated superior genotypes among the 28 loci were counted for each RIL. (**c**) *In silico* genotype data of 500 chromosome segment substitution lines (CSSLs) was generated by simulating five generations of backcross to 9311 followed with three generations of self-fertilization. The CSSLs containing superior alleles from Y58S were further intercrossed to combining more superior alleles.

Supplementary Table 1 Specificity evaluation of the genotype dataset of the rice hybrids using four deep-sequenced hybrid genomes

ID	Name	The coverage in genotyping and imputation	The coverage in quality estimation	Consistence
Z446	Tyou6135	3.44x	37.63x	98.06%
Z789	Gangyou118	1.07x	38.11x	97.72%
Z175	Teyouhang1	1.85x	39.81x	97.59%
Z341	Dyou527	1.76x	41.98x	97.81%

Supplementary Table 2 The list of 90 inbred parental lines sampled in the study.

ID	Name	Coverage	Subspecie	Type
A1	WufengA	13.49	*indica*	Male sterile line
A10	Qianjiang1A	11.61	*indica*	Male sterile line
A11	Zhejing4A	14.49	*japonica*	Male sterile line
A12	TianfengA	11.52	*indica*	Male sterile line
A13	JunA	17.40	*indica*	Male sterile line
A14	RongfengA	14.97	*indica*	Male sterile line
A15	Jin23A	12.15	*indica*	Male sterile line
A16	XianfengA	15.70	*indica*	Male sterile line
A17	ShiyouA	17.07	*indica*	Male sterile line
A18	II-32A	18.29	*indica*	Male sterile line
A19	Zhong9A	19.22	*indica*	Male sterile line
A2	Neixiang6A	17.28	*indica*	Male sterile line
A20	Chuanxiang29A	17.81	*indica*	Male sterile line
A21	GanxiangA	15.74	*indica*	Male sterile line
A22	ZhongxinA	18.07	*indica*	Male sterile line
A23	Guangkang13A	20.27	*indica*	Male sterile line
A24	10A	20.08	*indica*	Male sterile line
A25	Zhenshan97B	19.89	*indica*	Maintainer line
A3	Neixiang7A	18.02	*indica*	Male sterile line
A4	T109A	20.23	*indica*	Male sterile line
A5	85A	22.92	*indica*	Male sterile line
A6	XinrongA	16.12	*indica*	Male sterile line
A7	Hongyang1A	11.15	*indica*	Male sterile line
A8	XieqingzaoA	14.32	*indica*	Male sterile line
A9	Bing1A	18.24	*indica*	Male sterile line
S1	M6303S	15.29	*indica*	Male sterile line
S10	03S	14.71	*indica*	Male sterile line
S11	XinerS	13.98	*indica*	Male sterile line
S12	Xuan69S	11.20	*indica*	Male sterile line
S13	Guangzhan63S	7.98	*indica*	Male sterile line
S2	Y58S	13.06	*indica*	Male sterile line
S3	Zhu1S	14.15	*indica*	Male sterile line
S4	Guangzhan63-4S	17.30	*indica*	Male sterile line
S5	LongS	11.99	*indica*	Male sterile line
S6	XinanS	13.13	*indica*	Male sterile line
S7	Guangxiang24S	13.04	*indica*	Male sterile line
S8	Lu18S	13.98	*indica*	Male sterile line
S9	PA64S	11.84	*indica*	Male sterile line
T10	Yihui3551	10.68	*indica*	Restorer line
T100	Enhui80	11.38	*indica*	Restorer line
T101	R15	6.92	*indica*	Restorer line
T102	R1176	11.14	*indica*	Restorer line
T106	Xianhui207	15.01	*indica*	Restorer line
T107	R2122	10.03	*indica*	Restorer line
T113	Mianhui725	10.00	*indica*	Restorer line
T120	Jianghui151	11.35	*indica*	Restorer line
T122	Duoxi1	15.35	*indica*	Restorer line
T124	Neixianghui1	12.60	*indica*	Restorer line
T125	Luhui602	17.05	*indica*	Restorer line
T126	Chenghui448	18.66	*indica*	Restorer line
T13	Shuhui527	12.36	*indica*	Restorer line

T130	Fuhui676	18.43	*indica*	Restorer line
T136	Fuhui673	15.20	*indica*	Restorer line
T139	Minghui63	18.21	*indica*	Restorer line
T14	Zhehui7954	12.64	*indica*	Restorer line
T140	Minghui77	13.16	*indica*	Restorer line
T147	Yuexiangzhan	9.36	*indica*	Restorer line
T149	Guanghui128	10.68	*indica*	Restorer line
T15	Guanghui998	13.33	*indica*	Restorer line
T154	Gui99	15.40	*indica*	Restorer line
T158	Zhonghui8006	17.46	*indica*	Restorer line
T16	Feng986	9.30	*indica*	Restorer line
T160	Huazhan	15.95	*indica*	Restorer line
T18	Neihui94-11	17.74	*indica*	Restorer line
T19	Neihui92-4	18.34	*indica*	Restorer line
T191	R99082	16.55	*indica*	Restorer line
T2	Ronghui906	13.80	*indica*	Restorer line
T21	Neihui5550	13.68	*indica*	Restorer line
T22	Neihui99-14	19.18	*indica*	Restorer line
T23	Neihui2539	17.82	*indica*	Restorer line
T24	Neixianghui2	19.53	*indica*	Restorer line
T25	Neixianghui2156	18.31	*japonica*	Restorer line
T26	Fuhui718	16.94	*indica*	Restorer line
T27	Nanhui511	13.82	*indica*	Restorer line
T28	Lehui188	7.81	*indica*	Restorer line
T3	Yuehui360	16.31	*indica*	Restorer line
T31	R507	13.07	*indica*	Restorer line
T33	R6547	9.53	*indica*	Restorer line
T34	Zhenhui084	17.38	*indica*	Restorer line
T37	Xiangqing	17.10	*japonica*	Restorer line
T38	C418	11.90	*indica*	Restorer line
T4	Yuehui9113	12.97	*indica*	Restorer line
T40	Jinhui5	13.11	*indica*	Restorer line
T41	Fuhui305	12.44	*indica*	Restorer line
T5	9311	14.29	*indica*	Restorer line
T7	Minghui86	12.59	*indica*	Restorer line
T73	Fuhui838	11.43	*indica*	Restorer line
T8	Minghui100	13.90	*indica*	Restorer line
T9	Yihui1577	10.49	*indica*	Restorer line
D29	Zhongzao39	8.19	*indica*	Restorer line

Supplementary Table 3 Genome-wide screening of loss of heterozygosity loci in *indica* hybrids

Chr.	Position (Mb)*	Note
2	30.4 - 30.5	
6	6.0 - 6.1	Near *S5*
9	10.8 - 10.9	
10	12.6 - 12.7	
10	16.8 - 16.9	
11	2.0 - 2.1	
11	18.3 - 18.4	
12	14.5 - 14.6	

*Based on the rice reference genome IRGSP 4.0

Supplementary Table 4 Genome-wide detection of highly differentiated loci between male sterile lines and restorer lines

Chr.	Interval (Mb, IRGSP4)	F_{st}	Candidates
1	0.5-0.6	0.36	
1	1.4-1.6	0.40	
1	2.3-2.4	0.34	
1	3.5-3.7	0.37	
1	4.5-5.6	0.56	*Rf3*
1	8.9-9.3	0.43	
1	10.7-10.9	0.34	
1	21.7-22.1	0.36	
1	34.7-34.8	0.32	
1	37.3-41.5	0.48	
2	5.9-7.1	0.56	
4	26.3-26.4	0.54	
4	31.1-31.2	0.37	
4	32.6-33.0	0.38	
4	33.6-33.7	0.34	
5	24.1-24.2	0.37	
5	26.3-27.0	0.34	
6	1.0-3.1	0.41	*Hd3a*
6	4.9-5.5	0.39	*OsC1*
7	0.4-2.4	0.52	
7	7.1-7.2	0.33	
8	7.1-8.0	0.36	
9	0.7-5.6	0.34	
10	10.4-10.6	0.43	
10	15.0-15.2	0.36	*Ehd2*
10	19.3-19.4	0.32	*Rf4*
10	20.5-20.7	0.36	
10	21.8-22.6	0.43	
11	1.6-1.9	0.35	
11	2.9-3.0	0.31	
12	25.1-25.2	0.31	

[*]Threshold used in the list: $F_{st} > 0.3$

Supplementary Table 5 Genome-wide detection of selective sweep regions during modern breeding in *indica* population

Chr.	Interval (Mb, IRGSP4)	Selection signal ($\pi_{i\_landraces}/\pi_{i\_hybrid}$)*	Candidate genes
1	27.5 - 27.7	16.1	
1	40.1 - 40.3	30.7	*sd-1*
1	40.6 - 40.7	13.3	
1	43.2 - 43.3	15.1	
4	12.8 - 13.0	11.2	Domestication sweep
6	11.3 - 11.5	15.7	
6	12.5 - 12.8	13.3	
6	13.6 - 13.8	10.3	
7	6.1 - 6.2	24.2	Domestication sweep (*Rc*)
12	4.3 - 4.4	13.3	Domestication sweep

*Threshold used in the list: $\pi_{i\_landraces}/\pi_{i\_hybrid} > 10$

Supplementary Table 6 Genome-wide detection of selective sweep regions during modern breeding in *japonica* population

Chr.	Interval (Mb, IRGSP4)	Selection signal ($\pi_{j\_landraces}/\pi_{j\_hybrid}$)*	Candidate genes
1	15.3 - 15.4	35.2	
1	19.8 - 20.0	17.9	
1	33.1 - 33.4	36.1	
3	0.9 - 1.1	27.8	Domestication sweep(*OsSOC1*)
4	3.3 - 3.5	29.1	
4	20.8 - 20.9	16.6	
4	31.4 - 31.6	20.7	*NAL1*
5	4.9 - 5.2	15.1	Domestication sweep
6	0.8 - 0.9	32.6	
6	22.7-22.9	27.5	Domestication sweep

*Threshold used in the list: $\pi_{j\_landraces}/\pi_{j\_hybrid} > 15$

Supplementary Table 7 Phenotypic changes of yield and disease resistance between *indica* and *japonica* hybrids

Type	Yield per plant in Hangzhou (g)	Yield per plant in Sanya (g)	Blast resistance degree in Hangzhou*
Indica-indica cross	49.04	39.50	2.27
Japonica-japonica cross	35.53	30.59	5.17

*Degree 1 to 9 (from strong resistance to low resistance)

Supplementary Table 8 Phenotypic changes of grain quality between *indica* and *japonica* hybrids

Type	Brown rice rate	Milled rice ratio	Head rice ratio	Chalky grain rate	Chalkiness degree*	Alkali digestion value	Amylose content
Indica-indica cross	0.79	0.71	0.50	0.36	4.76	5.57	0.20
Japonica-japonica cross	0.83	0.74	0.55	0.22	2.77	6.72	0.14

*Degree 1 to 9 (from no chalkiness to high chalkiness)

Supplementary Table 9 Significant association signals ($P<10^{-6}$) of agronomic traits in Sanya

Trait	Chr.	Pos.	-log(P)*	Alleles	Heterozygosity	Known gene
Grain length	3	17,369,402	43.33	T/G	28.5%	*GS3*
Grain length	3	32,815,720	7.11	T/G	54.2%	
Grain length	5	28,177,704	7.57	T/G	51.3%	
Grain width	2	7,754,713	8.60	T/C	3.3%	*GW2*
Grain width	4	27,725,164	6.16	T/C	9.0%	
Grain width	5	5,340,811	38.14	T/C	16.1%	*qSW5*
Grain width	7	6,985,430	7.28	A/G	15.2%	
Grain width	11	7,208,216	7.27	A/T	5.7%	
Grain width	12	25,725,776	7.31	G/A	41.7%	
Grain number	3	29,257,210	8.99	T/C	7.7%	
Grain weight	2	7,754,713	6.83	T/C	3.3%	*GW2*
Grain weight	3	17,344,469	18.85	A/C	28.4%	*GS3*
Grain weight	5	5,340,811	9.20	T/C	16.1%	*qSW5*
Grain weight	7	24,424,387	6.30	C/T	44.8%	
Heading date	1	6,054,707	6.71	C/A	38.2%	
Heading date	3	1,233,319	20.52	G/A	24.1%	*OsSOC1*
Heading date	6	6,205,758	6.90	C/A	6.1%	
Heading date	6	9,538,190	6.70	T/C	60.9%	*Hd1*
Heading date	10	17,643,729	7.16	G/A	64.8%	*Ehd1*
Heading date	11	7,857,127	8.91	A/G	58.9%	
Plant height	1	5,075,247	9.60	A/T	10.3%	*Gn1 locus*
Plant height	3	1,230,055	16.80	A/G	19.6%	*OsSOC1*
Plant height	6	9,441,649	6.65	A/G	66.4%	*Hd1*
Plant height	8	6,570,736	6.06	G/A	42.6%	
Panicle length	12	20,083,173	6.27	A/G	3.6%	
Panicle length	11	4,605,171	6.29	A/C	54.1%	
Panicle number	3	22,276,018	7.19	A/G	7.6%	
Panicle number	5	14,837,373	6.21	C/T	29.1%	
Panicle number	6	1,161,081	8.73	T/C	12.2%	
Panicle number	12	1,725,907	10.39	C/T	6.9%	
Panicle number	12	18,945,793	7.93	A/G	2.8%	
Seed setting rate	5	717,289	6.69	C/T	13.9%	
Seed setting rate	11	3,245,148	6.21	A/G	5.0%	
Alkali digestion value	6	1,749,521	22.56	C/T	62.7%	*Waxy*
Alkali digestion value	6	6,929,482	58.80	T/A	44.6%	*ALK*
Alkali digestion value	7	642,766	7.14	C/T	13.0%	
Alkali digestion value	12	21,634,289	7.58	A/G	3.9%	
Amylose content	1	44,479,094	9.69	G/A	49.9%	
Amylose content	2	33,700,482	17.45	G/A	71.8%	
Amylose content	3	2,952,695	11.67	C/T	78.0%	
Amylose content	6	1,750,840	124.48	C/A	62.5%	*Waxy*
Amylose content	7	10,814,968	32.62	C/T	7.2%	
Amylose content	12	7,741,523	8.41	G/T	5.4%	
Brown rice rate	3	1,233,319	6.60	A/G	24.0%	*OsSOC1*
Brown rice rate	4	25,603,426	6.64	T/C	16.0%	
Brown rice rate	6	1,778,292	8.06	A/G	62.8%	*Waxy*
Chalkiness degree	5	5,340,811	24.16	T/C	16.2%	*qSW5*
Chalkiness degree	6	1,768,142	20.62	A/G	62.3%	*Waxy*
Chalky grain rate	2	33,701,236	7.33	A/G	65.1%	
Chalky grain rate	4	967,871	6.69	T/C	3.3%	
Chalky grain rate	5	5,340,811	17.80	T/C	16.2%	*qSW5*
Chalky grain rate	6	1,768,142	23.51	A/G	62.3%	*Waxy*
Chalky grain rate	11	5,374,791	7.70	T/C	9.7%	
Gel consistency	6	1,787,034	11.39	G/A	6.5%	*Waxy*

Gel consistency	6	6,746,299	13.05	G/A	47.0%	*ALK*
Head rice ratio	11	26,721,782	6.56	C/T	44.4%	
Milled rice ratio	3	1,266,260	7.67	G/A	25.1%	*OsSOC1*
Milled rice ratio	9	14,281,306	9.01	T/C	29.1%	
Transparence	5	5,341,170	6.72	A/G	16.3%	*qSW5*
Transparence	6	6,787,163	7.90	C/T	46.7%	*ALK*

*Showing the strongest associations among the inheritance models (additive, dominant and recessive)

Supplementary Table 10 Significant association signals (P <10^{-6}) of agronomic traits in Hangzhou

Trait	Chr.	Pos.	-log(P)*	Alleles	Heterozygosity	Known gene
Blast resistance	2	36,071,989	6.51	T/C	34.2%	*Pib*
Blast resistance	6	11,224,844	6.33	T/C	5.4%	*Pi2/Pi9 cluster*
Blast resistance	8	1,720,854	6.08	T/C	13.6%	
Blast resistance	8	6,161,524	11.93	C/A	64.8%	*Pi33*
Blight resistance	1	7,070,511	6.41	G/A	14.7%	
Blight resistance	6	29,194,860	7.08	A/G	5.1%	
Blight resistance	7	9,214,990	10.32	C/T	9.8%	
Blight resistance	11	29,096,478	6.76	A/G	46.5%	*Xa26*
Blight resistance	2	33,329,404	6.13	A/G	3.5%	
Blight resistance	7	16,920,754	9.20	G/A	64.4%	
Flag leaf length	1	6,562,276	8.83	G/A	37.7%	
Flag leaf length	6	2,915,690	6.39	T/C	60.6%	*Hd3a*
Flag leaf width	11	26,060,483	28.42	A/G	4.4%	
Flag leaf width	12	7,775,821	46.77	T/C	4.0%	
Flag leaf width	1	29,573,383	26.96	A/G	9.5%	
Flag leaf width	2	17,805,515	27.68	A/G	25.3%	
Flag leaf width	3	15,221,877	32.47	G/A	26.0%	
Flag leaf width	9	9,560,928	41.39	A/G	4.9%	
Grain length	3	1,064,741	6.26	G/A	25.9%	
Grain length	3	17,369,402	55.87	T/G	28.4%	*GS3*
Grain length	3	32,808,963	11.35	T/C	54.4%	
Grain number	4	31,493,318	8.05	A/T	10.2%	*NAL1*
Grain weight	2	7,726,595	7.85	A/G	3.6%	*GW2*
Grain weight	3	1,233,319	8.27	G/A	24.1%	
Grain weight	3	6,280,944	6.18	A/G	5.5%	
Grain weight	3	17,357,305	16.44	A/T	28.1%	*GS3*
Grain weight	3	32,809,424	8.57	A/G	57.3%	
Grain weight	5	5,341,575	9.27	G/A	16.2%	*qSW5*
Grain weight	11	5,453,912	7.07	G/A	3.4%	
Grain weight	4	13,312,631	6.19	A/G	15.0%	
Grain weight	7	28,067,364	6.52	G/T	49.3%	
Grain width	1	2,821,152	6.43	T/C	29.8%	
Grain width	2	7,726,595	9.95	A/G	3.6%	*GW2*
Grain width	3	17,392,585	8.24	T/C	25.6%	*GS3*
Grain width	4	7,928,259	8.54	A/G	12.1%	
Grain width	5	5,341,575	37.99	G/A	16.2%	*qSW5*
Grain width	11	7,181,824	6.59	C/T	5.8%	
Heading date	1	6,577,922	7.68	G/A	39.6%	
Heading date	3	1,120,422	19.28	G/C	25.2%	*OsSOC1*
Heading date	6	2,920,323	9.00	G/A	63.8%	*Hd3a*
Heading date	7	9,187,372	11.52	T/C	8.4%	*Ghd7*
Heading date	7	16,977,630	11.07	G/A	65.0%	
Heading date	8	4,313,191	14.69	T/C	8.3%	*Ghd8*
Heading date	9	21,807,130	6.15	T/C	22.9%	
Heading date	10	483,088	6.11	C/T	13.2%	
Heading date	2	28,730,271	6.71	T/C	20.5%	
Plant height	3	22,840,112	7.88	T/G	6.5%	
Plant height	3	29,948,289	6.55	C/T	36.7%	
Plant height	3	32,219,340	6.59	T/C	18.8%	
Plant height	6	2,918,710	7.68	T/C	61.2%	*Hd3a*
Plant height	8	6,100,823	9.47	C/T	3.8%	
Plant height	10	21,616,482	6.38	A/G	3.5%	
Plant height	3	789,687	6.08	A/G	27.4%	
Plant height	8	17,529,288	12.45	T/A	33.1%	
Plant height	11	19,270,506	11.53	A/G	18.3%	
Plant height	3	17,457,389	6.56	G/A	25.3%	

Plant height	2	21,487,525	6.05	T/C	34.9%	
Plant height	6	31,725,361	6.32	T/C	31.1%	
Leaf sheath color	6	5,016,982	59.88	A/G	60.7%	*OsC1*
Leaf sheath color	1	24,773,511	11.19	C/T	5.8%	
Panicle length	3	22,799,760	6.78	G/A	6.6%	
Panicle number	4	31,493,318	6.42	T/A	10.2%	*NAL1*

*Showing the strongest associations among the inheritance models (additive, dominant and recessive)

Supplementary Table 11 Significant association signals ($P < 10^{-6}$) in regional trials

Trait	Chr.	Pos.	-log(P)*	Alleles	Heterozygosity	Known gene
Plant height	3	777,489	10.61	A/G	24.9%	*OsSOC1*
Plant height	7	9,164,732	9.09	G/A	12.5%	*Ghd7*
Plant height	8	4,322,929	8.70	G/C	9.0%	*Ghd8*
Grain number	2	29,604,839	6.01	G/T	64.0%	
Grain number	7	9,083,208	6.36	C/T	69.9%	*Ghd7*
Grain number	8	4,309,956	8.07	A/G	9.6%	*Ghd8*
Panicle number	7	9,083,208	6.34	T/C	70.3%	*Ghd7*
Panicle number	3	790,016	10.06	C/T	18.7%	*OsSOC1*

*Showing the strongest associations among the inheritance models (additive, dominant and recessive)

Supplementary Table 12 Significant association signals ($P<10^{-6}$) for four yield-related traits using new-coded SNP[*]

Trait	Chr.	Pos.	-log(*P*)	Alleles	Heterozygosity
Heading date in Hangzhou	3	1,237,109	13.67	G/A	25.4%
Heading date in Hangzhou	8	4,314,336	12.34	C/T	8.3%
Plant height in Hangzhou	3	17,457,389	7.01	G/A	25.3%
Plant height in Hangzhou	3	22,840,112	6.46	T/G	6.5%
Plant height in Hangzhou	8	6,094,389	8.23	C/A	3.8%
Panicle number in Hangzhou	4	31,493,318	6.42	T/A	10.1%
Grain number in Hangzhou	1	6,346,698	6.30	T/C	38.9%
Grain number in Hangzhou	4	31,493,318	8.05	T/A	10.1%
Heading date in Sanya	3	1,234,050	14.53	T/A	19.7%
Plant height in Sanya	1	5,075,247	9.10	T/A	10.4%
Plant height in Sanya	3	1,234,050	13.28	T/A	19.7%
Grain number in Sanya	3	29,257,222	9.55	A/G	7.8%
Grain nubmer in Sanya	10	15,062,957	6.07	T/C	17.9%

[*]Both homozygous states were coded as one type and heterozygous genotype was coded as the other type

Supplementary Table 13 Modest significant association signals ($P < 10^{-4}$) of yield traits

Trait	Chr.	Pos.	-log(*P*)	Alleles	Heterozygosity	Known gene
Grain number in Sanya	1	3,121,627	4.65	A/G	7.4%	
	1	4,996,413	4.80	T/A	7.6%	*Gn1 locus*
	1	6,697,922	4.70	G/A	47.5%	
	3	450,122	5.30	C/T	41.6%	
	3	6,829,734	5.92	T/A	33.2%	
	3	17,999,452	4.40	T/C	35.9%	
	3	29,257,210	8.99	T/C	7.7%	
	4	8,908,005	5.12	A/G	8.8%	
	4	29,006,529	4.31	G/A	7.3%	
	5	55,003	5.84	T/C	9.3%	
	6	6,044,582	4.93	T/G	40.9%	
	7	9,080,855	4.40	A/T	70.2%	*Ghd7*
	7	15,952,928	5.49	C/A	25.4%	
	7	23,085,747	5.27	A/G	6.9%	
	8	25,265,573	4.67	A/G	3.5%	*OsSPL14*
	10	7,047,303	4.30	G/T	95.4%	
	10	12,826,404	5.47	T/C	5.6%	
	11	11,041,420	4.07	G/A	5.5%	
	11	14,854,910	4.37	G/A	56.0%	
	12	928,155	4.50	T/C	19.2%	
Panicle number in Sanya	2	7,211,215	10.26	C/A	3.4%	
	2	22,701,738	5.04	C/A	14.1%	
	2	32,978,192	4.98	T/G	44.5%	
	3	22,276,018	7.19	A/G	7.6%	
	5	14,837,373	6.21	C/T	29.1%	
	6	1,161,081	8.73	T/C	12.2%	
	7	13,298,310	4.81	A/T	4.0%	
	12	1,725,907	10.39	C/T	6.9%	
	12	11,439,227	4.57	T/C	7.0%	
	12	18,945,793	7.93	A/G	2.8%	
Yield per plant in Sanya	2	22,709,260	4.94	T/C	24.2%	
	3	503,913	4.47	G/A	26.8%	*OsSOC1*
	3	4,768,883	4.71	T/G	34.6%	
	3	13,440,516	4.28	A/G	14.0%	
	3	22,276,018	7.59	A/G	7.6%	
	6	6,858,284	4.60	G/C	50.1%	*ALK*
Grain number in Hangzhou	1	3,603,556	4.02	G/A	67.0%	
	1	5,745,311	5.15	A/G	45.7%	
	2	8,536,324	4.61	T/C	37.0%	
	3	32,281,901	4.12	T/C	34.0%	*Hd6*
	4	20,728,354	4.16	A/G	6.1%	
	4	28,857,502	4.24	G/A	29.4%	
	4	31,493,318	8.05	A/T	10.2%	*NAL1*
	6	14,816,452	4.60	C/A	35.5%	
	11	24,825,898	4.48	A/G	36.7%	
Panicle number in Hangzhou	2	391,166	4.69	T/G	3.6%	
	2	15,168,380	4.61	C/T	14.2%	
	2	22,560,376	4.65	A/G	32.8%	
	3	29,133,480	4.21	T/C	7.1%	
	4	31,493,318	6.42	T/A	10.2%	*NAL1*
	6	1,762,092	5.27	G/A	63.1%	*Waxy*

	8	28,295,345	5.10	A/G	14.4%	
	9	21,407,262	5.84	T/C	56.6%	*TAC1*
	10	3,047,094	4.42	T/A	51.0%	
Yield per plant in Hangzhou	1	7,259,550	5.33	C/A	4.3%	
	3	777,516	5.82	T/G	24.7%	*OsSOC1*
	3	9,316,169	5.06	G/C	44.0%	
	4	1,634,703	4.18	C/G	2.4%	
	4	20,975,549	5.26	C/T	56.6%	
	6	1,969,527	4.45	A/C	63.9%	*Waxy*
	6	2,919,919	4.13	T/C	60.0%	
Panicle number in regional trials	2	6,240,269	4.23	A/C	5.3%	
	2	17,451,510	4.88	G/T	32.1%	
	2	32,483,090	4.09	C/T	53.3%	
	3	790,016	10.06	C/T	18.7%	*OsSOC1*
	3	34,246,261	4.05	A/T	4.7%	
	7	17,246,061	4.44	T/C	56.6%	
	8	25,250,452	4.29	T/C	9.7%	*OsSPL14*
	8	27,009,147	5.82	C/T	2.8%	
	9	11,866,028	4.87	T/C	10.2%	
	10	469,461	4.05	A/C	19.0%	
	10	16,976,375	5.90	A/G	3.5%	*Ehd1*
	11	19,792,541	5.55	C/T	46.2%	
	12	26,082,136	4.93	C/T	7.2%	
Grain number in regional trials	1	6,413,066	4.36	C/T	32.2%	
	2	29,604,839	6.01	G/T	64.0%	
	6	20,290,659	4.57	G/A	4.9%	
	6	24,667,669	4.38	T/G	17.9%	
	6	25,989,952	4.76	C/T	13.0%	
	7	8,756,609	4.04	G/A	65.1%	*Ghd7*
	7	17,428,905	4.26	G/T	63.7%	
	8	4,309,956	8.07	A/G	9.6%	*Ghd8*
	8	25,194,048	6.37	C/A	4.1%	*OsSPL14*
	9	20,540,281	5.54	A/G	38.0%	
	10	13,321,201	5.25	C/T	7.6%	
	10	17,374,431	4.06	G/A	11.6%	*Ehd1*
	11	2,393,350	4.99	A/C	68.3%	*RCN1*
	11	11,358,305	4.24	C/G	28.0%	
	11	26,468,657	4.29	C/T	4.4%	
	11	28,171,133	4.84	G/A	32.6%	
	12	5,773,951	4.74	G/T	4.5%	
Yield per unit in regional trials	3	1,630,897	6.15	A/T	20.0%	*OsSOC1*
	3	10,475,304	4.35	A/G	25.0%	
	4	22,297,941	5.02	G/A	17.1%	
	8	4,309,956	5.10	A/G	8.8%	*Ghd8*
	8	26,232,199	4.21	T/C	60.3%	*OsSPL16*
	9	7,162,499	5.79	C/T	54.1%	
	10	18,857,886	4.97	C/A	33.9%	

Supplementary Table 14 The associated loci with extremely high heterozygosity

Trait	Chr.	Pos.	Known gene	-log(*P*)	Heterozygosity
Leaf sheath color	6	5016982	*OsC1*	59.88	0.61
Height	6	2918710	*Hd3a*	7.68	0.61
Height	6	9441649	*Hd1*	6.65	0.66
Heading date	6	9538190	*Hd1*	6.7	0.61
Heading date	10	17643729	*Ehd1*	7.16	0.65

Supplementary Table 15 Effects of grain quality associations on yield per plant in Sanya

			Chalky grain rate			Yield per plant			
Chr.	Pos. (IRGSP4)	-log(*P*)	Homo Geno1	Hete Geno	Homo Geno2	Homo Geno1	Hete Geno	Homo Geno2	Known gene
2	33701236	7.33	NA	0.43	0.25	NA	40.0	38.5	
4	967871	6.69	0.37	0.23	NA	39.5	38.2	NA	
5	5340811	17.80	0.68	0.65	0.30	43.9	42.0	38.9	*qSW5*
6	1768142	23.51	0.48	0.41	0.17	42.1	39.8	37.2	*Waxy*
11	5374791	7.70	NA	0.63	0.34	NA	41.3	39.3	

Supplementary Table 16 Phenotypic changes of agronomic traits between Hangzhou and Sanya

Trait	Average in Hangzhou	Average in Sanya	Pearson correlation *r*	*P*-value
Yield per plant /g	49.04	39.50	0.0601	2.3E-02
Panicle number	14.03	11.69	0.1537	5.5E-09
Grain number	180.24	142.81	0.3463	2.3E-41
Seed setting rate	0.76	0.87	0.0913	5.7E-04
Grain weight /g	28.01	29.67	0.8526	0
Heading date /days	82.29	87.71	0.3443	3.5E-41
Height/cm	117.13	91.27	0.6057	3.3E-144
Panicle length /cm	26.59	22.18	0.4287	7.2E-65
Grain length /mm	9.51	8.95	0.9140	0
Grain width /mm	2.67	2.67	0.8876	0

Supplementary Table 17 Genomic loci with strong over-dominance effects in rice genome

Trait	Chr.	Pos.	-log(*P*)	Superior allele	Alternative allele	*d/a*	MPH
Grain number in Sanya	11	14854910	4.37	G	A	10.5	0.04
Grain number in Hangzhou	1	3603556	4.02	G	A	-1.75	-0.04
Panicle number in Sanya	12	11439227	4.57	T	C	4.09	0.11
Panicle number in Sanya	7	13298310	4.81	A	T	13.28	0.17
Panicle number in Hangzhou	10	3047094	4.42	T	A	-34	-0.03
Height in Sanya	8	6531960	4.34	A	C	-1.85	-0.07
Height in Sanya	7	2275142	4.66	G	A	2.08	0.02
Height in Hangzhou	8	8342836	4.18	T	A	1.79	0.05
Height in Hangzhou	12	14696875	5.41	G	A	2.28	0.02
Height in Hangzhou	1	16119234	5.74	C	T	2.99	0.01
Yield per plant in Sanya	3	13440516	4.28	A	G	6.08	0.06
Yield per plant in Hangzhou	3	9316169	5.06	G	C	4.05	0.02
Yield per plant in Hangzhou	6	2919919	4.13	T	C	5.85	0.04
